# Associations between changes in the gut microbiota and liver cirrhosis: a systematic review and meta-analysis

**DOI:** 10.1186/s12876-025-03589-5

**Published:** 2025-01-13

**Authors:** Ye Liu, Ziwei Chen, Chang Li, Tianhan Sun, Xuanmei Luo, Boyue Jiang, Meilan Liu, Qing Wang, Tong Li, Jianfu Cao, Yayu Li, Yuan Chen, Lu Kuai, Fei Xiao, Hongtao Xu, Hongyuan Cui

**Affiliations:** 1 Beijing Hospital, Peking University Fifth School of Clinical Medicine, National Center of Gerontology, Beijing, China; 2 Clinical Biobank, Beijing Hospital, National Center of Gerontology, Institute of Geriatric Medicine, Chinese Academy of Medical Sciences, Beijing, China; 3 The Key Laboratory of Geriatrics, Beijing Institute of Geriatrics, Institute of Geriatric Medicine, Chinese Academy of Medical Sciences, Beijing Hospital, National Center of Gerontology of National Health Commission, Beijing, China; 4 Department of General Surgery, Department of Hepato-Bilio-Pancreatic Surgery, Beijing Hospital, National Center of Gerontology, Institute of Geriatric Medicine , Chinese Academy of Medical Sciences & Peking Union Medical College, Beijing, China; 5 Department of Laboratory Medicine, Beijing Hospital, National Center of Gerontology, Institute of Geriatric Medicine, Chinese Academy of Medical Sciences, Beijing, China; 6 Department of General Surgery, Beijing Hospital, No. 1 Dahua Road, Dong Dan, Beijing, 100730 China; 7 Department of Laboratory Medicine, Beijing Hospital, No. 1 Dahua Road, Dong Dan, Beijing, 100730 China; 8 Clinical Biobank, Beijing Hospital, No. 1 Dahua Road, Dong Dan, Beijing, 100730 China

**Keywords:** Liver cirrhosis, Gut microbiota, Intestinal microbiota, Meta-analysis

## Abstract

**Objective:**

Summaries of the relationships between the microbiota and liver cirrhosis and their conclusions are not consistent. This study describes microbial differences in patients with liver cirrhosis by performing a meta-analysis.

**Methods:**

We searched PubMed, Embase, Web of Science, and the Cochrane Library and collected related articles published before March 10, 2024. Ratio of autochthonous to non-autochthonous taxa was calculated as the cirrhosis dysbiosis ratio (CDR). Using a random-effects model, the standard mean deviation (SMD) and 95% confidence interval (CI) were calculated. We subsequently performed subgroup, sensitivity, and publication bias analyses. cirrhosis dysbiosis ratio.

**Results:**

A total of 53 eligible papers including 5076 participants were included. The pooled estimates revealed a moderately significant reduction in gut microbiome richness in patients with liver cirrhosis compared with controls, including the Shannon, Chao1, observed species, ACE, and PD indices, but no significant difference was observed for the Simpson index. Over 80% of the studies reported significant differences in β diversity. Families Enterobacteriaceae and Pasteurellaceae, belonging to the phylum Proteobacteria, along with the family Streptococcaceae and the genera *Haemophilus*, *Streptococcus*, and *Veillonella*, were significantly associated with liver cirrhosis compared to the control group. In contrast, the healthy group exhibited a higher abundance of the class Clostridia, particularly the families Lachnospiraceae and Ruminococcaceae, which are known for their diversity and role as common gut commensals. Furthermore, the class Bacilli, predominantly represented by the genus *Streptococcus*, was markedly enriched in the cirrhosis group.

**Conclusions:**

The microbiota richness of liver cirrhosis patients was lower than that of healthy controls. Alterations in gut microbiota linked to liver cirrhosis were characterized by a decrease in Lachnospiraceae, Ruminococcaceae, and Clostridia and an enrichment of Enterobacteriaceae, Pasteurellaceae, Streptococcaceae, Bacilli, and *Streptococcus*.

**Supplementary Information:**

The online version contains supplementary material available at 10.1186/s12876-025-03589-5.

## Introduction

Over the past decade, more research has revealed that the human gut microbiome plays important roles in human health and disease. Trillions of bacteria reside within the gastrointestinal tract. Numerous diseases, including gastroenterological, neurological, respiratory, metabolic, hepatic, and cardiovascular disorders, have been linked to the gut microbiota [1]. Alterations in the gut microbiota and an impaired intestinal barrier are associated with cirrhosis [2]. Changes in the gut‒liver axis disrupt the intestinal barrier, increasing the portal influx of bacteria or their products to the liver and causing or worsening a variety of hepatic diseases [2, 3]. In recent years, knowledge of alterations in the gut microbiota in patients with cirrhosis has improved with the use of 16S RNA and metagenomic sequencing [4, 5]. A microbial imbalance has been shown in numerous studies to contribute to the occurrence, development, and deterioration of liver cirrhosis [6, 7]. The gut microbiota is increasingly implicated in different liver diseases.

However, few studies have focused on the relationship between the microbiota and liver cirrhosis, and their conclusions are not consistent. A previous study revealed that *Streptococcus salivarius*, which contains gut urease, was significantly abundant in cirrhotic patients. *S. salivarius* is expected to be a potential biomarker of the efficacy of ammonia-lowering therapies in cirrhotic patients with minimal hepatic encephalopathy (HE) [8]. Caussy et al. [9] reported the enrichment of *Megasphaera*, *Gallibacterium*, and *Streptococcus* in the cirrhotic group, which was consistent with the findings of a study performed by Ponziani and colleagues in an Italian cohort [10]. Other taxa, including *Veillonella*, *Dialister*,* Megasphaera*, *Atopobium*, and *Prevotella*, were more common in patients with cirrhosis than in healthy individuals [11]. The study of microbe‒host interactions and the identification of specific microbes or microbial products as biomarkers have also garnered increasing attention [9, 12, 13]. Identifying cirrhosis-specific microbiota that are consistent with other studies would have major clinical implications. However, the relationship between the microbiota and liver cirrhosis could not be adequately examined due to the small sample sizes included in the published studies. Moreover, the study of the microbiota is a rapidly evolving field, and many other articles on cirrhosis have been published in recent years. Previous studies have not demonstrated a clear consistent association or provided sufficient evidence.

In a meta-analysis, the available data are synthesized to form a larger sample size and to provide more precise estimates. As part of our effort to comprehensively and systematically describe the relationship between the microbiota level and liver cirrhosis and to evaluate the relationship between gut microbiota dysbiosis and health, we conducted a comprehensive literature search and meta-analysis.

## Methods

We referred to the Preferred Reporting Items for Systematic Reviews and Meta-Analysis (PRISMA) statement to perform this research [14]. This study was registered at the National Institute for Health Research PROSPERO, International Prospective Register of Systematic Reviews (registration number: CRD42022311876).

### Search strategy

Two authors independently performed the literature search in PubMed, Web of Science, Embase, and the Cochrane Library. The literature was limited to English studies published before March 10, 2024. The following key terms were used for the search: “gut microbiome”, “microbiota”, “microbiome”, “bacteria”, “microbial”, “flora”, “microbe”, “cirrhosis”, “cirrhotic liver”, and “hepatic cirrhosis”. The detailed search strategies used are available in Supplementary Table S1. In addition, we excluded case reports, comments, and review articles. Articles in the references were also searched to add relevant studies. We also tried to contact corresponding authors of potentially relevant articles that lacked the necessary data for meta-analysis.

### Inclusion and exclusion criteria

We included cross-sectional, longitudinal, and case‒control studies on the relationship between the gut microbiota and liver cirrhosis. Articles were included in the final analysis if they met the following criteria: (1) a diagnosis of liver cirrhosis in the patient group; (2) liver biopsy, laboratory test, radiographic, or FibroScan findings were available; (3) the control group was healthy individuals; (4) the microbiota was detected in both groups; (5) the sample size in each group was provided; and (6) the language was English. Studies were excluded if they (1) included patients with gastrointestinal diseases or other related diseases affecting the gut microbiota; (2) included individuals who received antibiotics, probiotics, or prebiotics within 1 month before sampling; (3) had insufficient data; (4) were review articles, comments, meeting abstracts, case reports or animal or cell articles; (5) were duplicate articles; or (6) were articles not related to the microbiota, microbiome, or bacteria.

### Data extraction and quality assessment

The following data were extracted by two authors from the final studies: first author, publication year, publication type, country, patient characteristics, age of patients, body mass index (BMI), aetiology of cirrhosis, Child‒Pugh score, numbers of patients and controls, microbiota detection methods, and quality score. In addition, we extracted the α diversity indices (means (M) and SDs/medians and quartiles) to evaluate the richness and evenness of the microbial community, β diversity and taxonomic findings at the phylum, family, and genus levels (reported abundance). The cirrhosis dysbiosis ratio (Ruminococaceae + Lachnospiraceae + Veillonellaceae + Clostridiales Cluster XIV / Bacteroidaceae + Enterobacteriaceae) was also calculated and compared between groups.

For measures of α diversity and the abundance of microbial taxa, if a study provided only the medians and interquartile ranges for the original data, we used a web-based tool (https://www.math.hkbu.edu.hk/~tongt/papers/median2mean.html) to convert the data to means and SDs. If necessary, WebPlot digitizer (V.4.5) [15] was employed to extract digital data from the picture.

We used the Newcastle‒Ottawa Scale (NOS) to assess the quality of the included papers [16]. The total NOS score ranges from 0 to 9, which indicates lower to better quality.

### Summary Outcome measures and statistical analysis

The microbiota of each group was measured based on the mean and standard deviation (SD) values. The combined effect was measured using the standard mean deviation (SMD) and 95% confidence interval (CI). The subgroup analysis focused on the aetiology, diagnosis, sequencing method, region, size, and stage. We also assessed heterogeneity using *I*^*2*^ statistics. The pooled effects with 95% CIs were calculated using random effects models based on the DerSimonian and Laird method. We qualitatively summarized the results for β diversity in the included studies by plotting a heatmap. Although we extracted data from the pictures, original data could not be obtained for most taxa. Therefore, we summarized the taxon results and compared the differences reported in the literature, described as increased, decreased, or “not consistent”. See Supplementary Tables S9–11 for details.

We performed a sensitivity analysis by omitting one study at a time to evaluate the influence of each study. Potential publication bias was assessed using Begg’s and Egger’s tests. Statistical significance was set to *P* < 0.05. R 4.4.0 was used for the meta-analysis.

## Results

### Characteristics and quality of the included studies

**Fig. 1:**
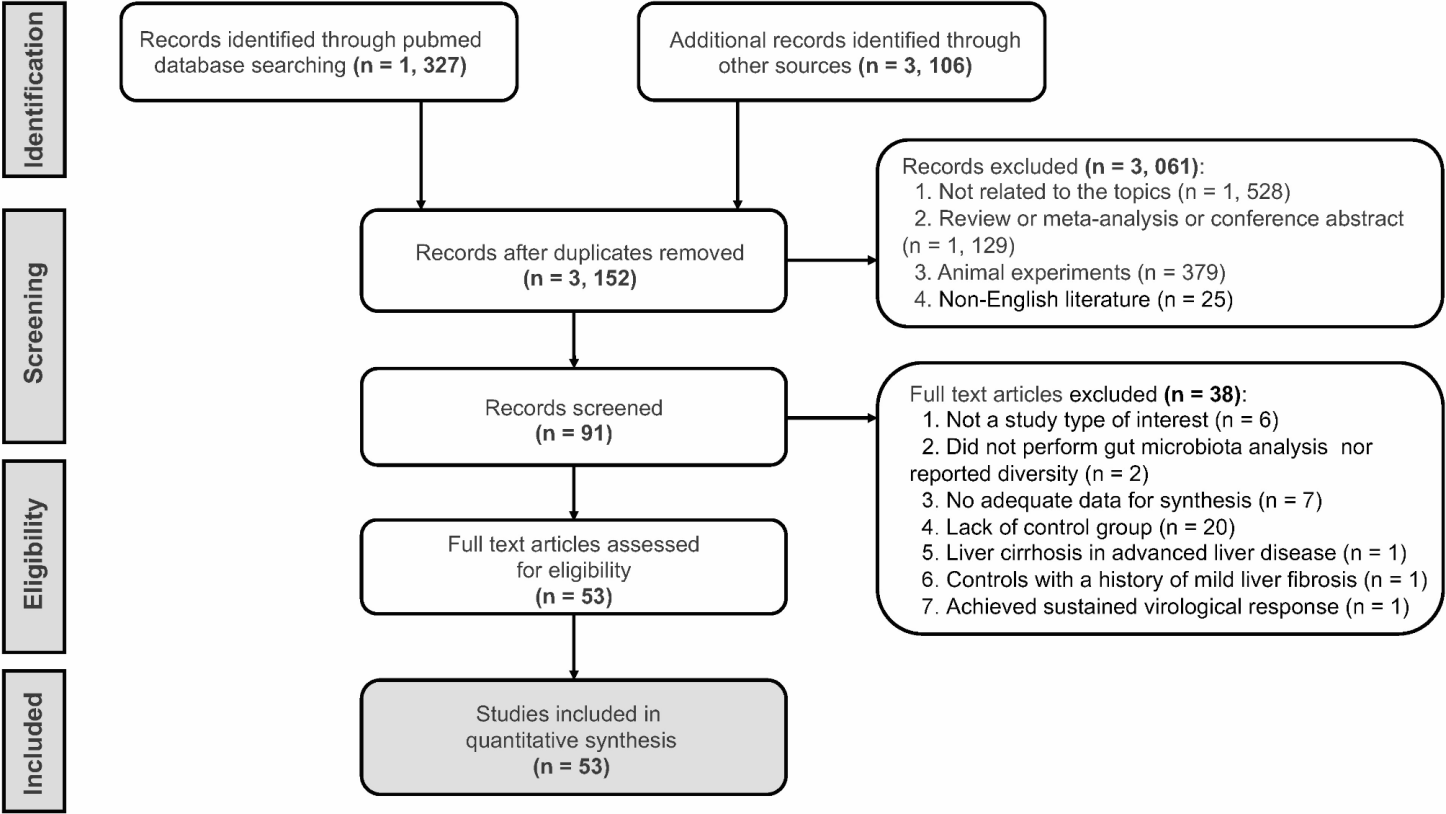
Flow diagram of the literature search

The online databases were first searched for 4,433 articles. After deduplication, 3,152 articles remained. Then, we selected abstracts and checked the data. Ultimately, 53 articles were analysed in this study. A detailed explanation of the selection procedure can be found in Fig. 1. The details of the excluded articles are listed in Supplementary Table S4.

Table 1 and Supplementary Tables S5–6 summarize the features of the included articles. A NOS quality score of 5 to 9 was assigned to each article (detailed in Supplementary Tables S2–3). The gut microbiota were obtained from faecal samples. The majority of studies were case–control studies (30/53), one was a cohort study, and twenty-two were cross-sectional studies. With respect to the individual studies, the total number of included participants varied from 13 to 385 (median of 60). Over half of the studies (31 [58.5%]) were conducted in Eastern countries, and 22 (41.5%) were conducted in Western countries. The ages were 42–73 (median 55.6) years for the patients and 23–72.5 (median 48.6) years for the controls. The diagnosis of liver cirrhosis was based on ultrasonography (*n* = 4), liver biopsy (*n* = 5), and a clinical examination (*n* = 1), and the remaining patients were diagnosed with a mix of different methods (*n* = 39) or the methods were not reported (*n* = 4). The most commonly used method of stool processing was 16S ribosomal RNA (rRNA) sequencing (*n* = 41), followed by quantitative polymerase chain reaction (qPCR) or real-time quantitative polymerase chain reaction (*n* = 2), shotgun metagenomics (*n* = 6), pyrosequencing (*n* = 2), and sequencing of the fungal-specific internal transcribed spacer 1 (ITS1) or 18S rRNA (*n* = 2). The aetiologies of cirrhosis included hepatitis B virus (HBV) infection, hepatitis C virus (HCV) infection, alcoholic cirrhosis, nonalcoholic steatohepatitis (NASH), nonalcoholic fatty liver disease (NAFLD), and a mix of different aetiologies.

**Table 1:** Characteristics of the included studies and subjects

Study	Country	Study design	Diagnosis of LC	*N* (cases)	*N* (controls)	Males, *n* (cases, %)	Males, *n* (controls, %)	Age (cases, yrs)	Age (controls, yrs)	BMI (cases)	BMI (controls)
Chen et al., 2011 [4]	China	Case‒control study	Liver biopsy, clinical tests and radiology	36	24	25 (69)	14 (58)	49 ± 11	46 ± 8	20.3 ± 1.1	19.8 ± 1.2
Lu et al., 2011 [41]	China	Case‒control study	Ultrasonography and liver biopsy	31	32	9 (29)	10 (31)	49.0 ± 4.8	43.1 ± 5.2	21.7 ± 1.3	21.6 ± 1.1
Zhang et al., 2013 [8]	China	Case‒control study	Ultrasonography and liver biopsy	25	26	16 (64)	8 (31)	59 ± 11	43 ± 13	22.6 ± 3.4	22.3 ± 1.5
Wei et al., 2013 [42]	China	Case‒control study	Liver biopsy	120	120	73 (61)	53 (44)	47.3	48 ± 6	21.5	23.2 ± 1.1
Bajaj et al., 2014 [31]	USA	Cross-sectional	Liver biopsy	219	25	56 (26)	8 (32)	57.0	55.7 ± 8.5	29.6	29.5 ± 5.6
Qin et al., 2014 [6]	China	Case‒control study	Ultrasonography and liver biopsy	98	83	NA	NA	50 ± 11	42 ± 9	NA	NA
Tuomisto et al., 2014 [43]	Finland	Case‒control study	Liver biopsy	12	7	12 (100)	7 (100)	58 (39–73)	45 (26–57)	28.1 (19.7–39.2)	27.1 (20.8–37.2)
Ahluwalia et al., 2016 [44]	USA	Case‒control study	Liver biopsy, clinical tests and radiology	147	40	62 (42)	23 (58)	55.4	55.1 ± 13.4	NA	NA
Bajaj et al., 2016 [29]	USA	Cross-sectional	Liver biopsy, clinical tests and radiology	105	45	80 (76)	36 (80)	58.8	55.5 ± 7.9	29.4	28.5 ± 5.8
Chen et al., 2016 [11]	China	Case‒control study	Liver biopsy, clinical tests, and ultrasonography	30	28	23 (77)	22 (79)	49 ± 8	52 ± 9	22.8 ± 3.3	22.9 ± 2.8
Wei et al., 2016 [45]	China	Case‒control study	Liver biopsy	10	3	8 (80)	1 (33)	50.3	46.7	22.7	21.0
Lee et al., 2023 [46]	China	Case‒control study	Liver biopsy, clinical tests, and ultrasonography	21	16	17 (81.0)	13 (81.3)	58.8 (50.9–63.6)	58.4 (49.6–64.5)	27.5 (25.1–29.4)	23.3 (22.3–24.7)
Heidrich et al., 2018 [12]	Germany	Cross-sectional	Ultrasonography	38	50	20 (53)	25 (50)	58.2 ± 10.9	39.5 ± 16.5	26.7 ± 7.0	25.4 ± 5.0
Bajaj et al., 2018 [47]	USA	Case‒control study	Liver biopsy, clinical tests, and radiology	202	94	150 (74)	50 (53)	52.5	60.6	NA	NA
Bajaj et al., 2017 [48]	USA	Cross-sectional	Liver biopsy, clinical tests, and radiology	143	26	104 (73)	17 (65)	55.6	52.8 ± 8.4	NA	NA
Bajaj et al., 2018 [49]	USA	Cross-sectional	Liver biopsy, clinical tests, and radiology	154	26	115 (75)	NA	56.4	NA	NA	NA
Inoue et al., 2018 [50]	Japan	Cross-sectional	Ultrasonography	40	23	19 (48)	14 (61)	69.2 ± 9.8	60.5 ± 8.1	NA	NA
Liu et al., 2018 [51]	China	Cross-sectional	Liver biopsy, clinical tests, and ultrasonography	36	20	17 (47)	12 (60)	46	47 (32–60)	22.0	22.4 (18.5–25.4)
Ponziani et al., 2018 [52]	Italy	Case‒control study	Liver biopsy, clinical tests, and radiology	12	12	5 (42)	4 (33)	73 (62.75-78)	71 (63.42-75.2)	24 (21–25.25)	23 (22.37–24.15)
Shao et al., 2018 [53]	China	Case‒control study	Ultrasonography and liver biopsy	95	47	64 (67)	31 (66)	49.5 ± 10	45.3 ± 8	22.8 ± 2.89	21.98 ± 1.81
Sun et al., 2018 [54]	China	Case‒control study	Clinical tests and ultrasonography	20	37	13 (65)	27 (73)	47.6 ± 7.2	48.1 ± 12.2	24.45 ± 2.54	23.07 ± 2.27
Caussy et al., 2019 [9]	USA	Cross-sectional	Ultrasonography and liver biopsy	26	54	7 (27)	15 (28)	65.1 ± 9.8	45.9 ± 19.9	31.3 ± 6.1	26.2 ± 6.8
Deng et al., 2019 [55]	China	Case‒control study	NA	80	20	55 (69)	NA	42.30 ± 13.15	NA	NA	NA
Jin et al., 2019 [56]	Canada	Case‒control study	Liver biopsy, clinical tests, and ultrasonography	17	17	8 (47)	NA	58 (51-60)	NA	NA	NA
Zheng et al., 2020 [57]	China	Case‒control study	Liver biopsy, clinical tests, and ultrasonography	24	20	16 (67)	13 (65)	58.08 ± 6.93	56.70 ± 8.47	22.90 ± 1.59	23.22 ± 1.94
Sung et al., 2019 [58]	China	Case‒control study	Clinical tests and ultrasonography	35	13	24 (69)	7 (54)	59.8	47.2 ± 10.2	NA	NA
Astbury et al., 2020 [59]	UK	Cross-sectional	Liver biopsy	25	76	10 (40)	9 (12)	64.0 ± 2.0	66.3 ± 1.06	34.0 ± 1.1	26.5 ± 0.5
Bajaj et al., 2020 [30]	USA	Case‒control study	Liver biopsy and ultrasonography	194	81	75 (39)	26 (32)	59.6	57.9	NA	NA
Chen et al., 2020 [60]	China	Cohort	Ultrasonography	25	21	20 (80)	16 (76)	51.24 ± 6.91	45.14 ± 9.03	21.87 ± 2.61	21.97 ± 2.91
Cox et al., 2020 [61]	UK	Case‒control study	Liver biopsy, ultrasonography, clinical tests, and radiology	202	94	150 (74)	50 (53)	60.6	52.5	27.4	25.9
Lapidot et al., 2020 [25]	Israel	Case‒control study	Liver biopsy, ultrasonography, and clinical tests	68	27	46 (68)	21 (77)	65.7	61.6	27.8	25.7
Oh et al., 2020 [62]	USA	Case‒control study	Liver biopsy, ultrasonography, and clinical tests	27	54	5 (19)	15 (28)	64.74 ± 9.80	45.85 ± 19.86	32.85 ± 10.06	26.07 ± 6.83
Sydor et al., 2020 [63]	Germany	Case‒control study	Liver biopsy and clinical tests	30	20	NA	NA	65.5	23.3 ± 2.8	31.4	23.3 ± 2.5
Yang et al., 2020 [64]	China	Cross-sectional	Liver biopsy, ultrasonography, and clinical tests	54	31	NA	NA	50.5 (33–85)	33 (23–40)	NA	NA
Zeng et al., 2020 [65]	China	Cross-sectional	Liver biopsy, ultrasonography, and clinical tests	25	15	19 (76)	10 (67)	48.40 ± 13.16	30.80 ± 6.21	22.52 ± 2.95	21.56 ± 1.90
Huan et al., 2021 [66]	China	Cross-sectional	Ultrasonography	36	19	29 (81)	12 (63)	50.3	48.5	22.9	23.6
Ponziani et al., 2021 [67]	Italy	Cross-sectional	NA	50	36	35 (70)	21 (58)	67.5	72.5 (58.25–75.25)	27.9	26.2 (24.39–28.68)
Ren et al., 2021 [68]	China	Cross-sectional	Liver biopsy, ultrasonography, and clinical tests	60	30	36 (60)	17 (57)	43.9	44.17 ± 11.17	23.41	NA
Alvares-da-Silva et al., 2022 [69]	Brazil	Case‒control study	Liver biopsy and ultrasonography	280	105	177 (63)	43 (41)	60.7	57.2	NA	NA
Baltazar-Díaz et al., 2022 [70]	Mexico	Cross-sectional	Liver biopsy, ultrasonography, and clinical tests	18	18	18 (100)	18 (100)	49.89 ± 11.49	48.72 ± 8.63	24.66 ± 3.82	25.85 ± 2.86
Hua et al., 2022 [71]	China	Cross-sectional	NA	20	10	15 (75)	7 (70)	60.65 ± 12.42	59.21 ± 11.09	NA	NA
Maslennikov et al., 2022 [72]	Russia	Cross-sectional	Liver biopsy, ultrasonography, and clinical tests	46	14	18 (39)	3 (21)	55 (43–61)	51 (41–63)	27.0 (23.6–30.1)	21.1 (19.7–26.0)
Shu et al., 2022 [73]	China	Cross-sectional	Liver biopsy, ultrasonography, and clinical tests	50	30	40 (80)	19 (63)	54.5	45.83 ± 23.99	NA	NA
Sun et al., 2022 [74]	China	Case‒control study	Ultrasonography and clinical tests	30	20	25 (83)	14 (70)	54.53 ± 8.15	51.20 ± 8.43	24.10 ± 2.43	23.07 ± 3.20
Ullah et al., 2022 [75]	China	Case‒control study	Clinical tests	8	9	7 (88)	4 (44)	59.0 ± 3.1	41.7 ± 4.0	NA	NA
Zhou et al., 2022 [76]	China	Cross-sectional	Liver biopsy, ultrasonography, clinical tests and radiology	60	30	46 (77)	20 (67)	60.5	57 (55, 60.3)	NA	NA
Chen et al., 2023 [77]	China	Case‒control study	Liver biopsy, ultrasonography, and clinical tests	21	20	14 (67)	10 (50)	56.52 ± 7.61	54 ± 8.21	24.14 ± 3.12	24.30 ± 2.93
Lai et al., 2023 [78]	China	Case‒control study	Liver biopsy, ultrasonography, and clinical tests	18	17	12 (67)	7 (41)	65.2 ± 11.4	56.7 ± 9.8	26.2 ± 3.6	23.1 ± 1.51
Wang et al., 2023 [28]	China	Case‒control study	Liver biopsy, ultrasonography, and clinical tests	30	20	20 (67)	12 (60)	52.23 ± 9.00	44.45 ± 8.46	22.21 ± 3.24	22.37 ± 2.07
Wu et al., 2023 [79]	China	Cross-sectional	Liver biopsy, ultrasonography, and clinical tests	28	16	17 (61)	9 (56)	54.79 ± 12.68	54.25 ± 10.14	NA	NA
Yan et al., 2023 [80]	China	Cross-sectional	Liver biopsy, ultrasonography, and clinical tests	30	30	25 (83)	NA	51.33 ± 9.79	NA	NA	NA
Zhang et al., 2023 [81]	China	Case‒control study	NA	25	15	19 (76)	10 (67)	48.4 ± 13.16	30.8 ± 6.21	22.52 ± 2.95	21.55 ± 1.9
Efremova et al., 2024 [82]	Russia	Cross-sectional	Liver biopsy and clinical tests	47	27	18 (38)	NA	49 (44-56)	NA	26.1 (24.5-30.0)	NA

Study	Aetiology of cirrhosis	Child-Pugh class (*n*)					Complications	Detection method	Diversity assessments	Quality scores	
		A		B		C					
Chen et al., 2011 [4]	HBV (*n* = 24) Alcohol (*n* = 12)	NA		NA		NA	None	16S rRNA V3	α: Chao1, Shannon β: UniFrac	7	
Lu et al., 2011 [41]	HBV	NA		NA		NA	NA	qPCR	α: Shannon β: NA	7	
Zhang et al., 2013 [8]	AIH (*n* = 2) HBV (*n* = 12) PBC (*n* = 3) PSC (*n* = 1) Alcohol (*n* = 7)	12		11		2	Ascites	Pyrosequencing	α: Chao1, Faith’s PD, Observed species β: UniFrac	7	
Wei et al., 2013 [42]	HBV	40		40		40	None	Metagenomic sequencing	α: NA β: PCA	8	
Bajaj et al., 2014 [31]	HCV (*n* = 87) Alcohol (*n* = 43) HCV + alcohol (*n* = 32) NASH (*n* = 32) Other (*n* = 25)	NA		NA		NA	Infection	Pyrosequencing	α: NA β: PCA	9	
Qin et al., 2014 [6]	HBV Alcohol	NA		NA		NA	NA	Metagenomics	α: NA β: NA	8	
Tuomisto et al., 2014 [43]	Alcohol	NA		NA		NA	Ascites	qRT‒PCR	α: NA β: NA	7	
Ahluwalia et al., 2016 [44]	HCV (*n* = 48) Alcohol (*n* = 31) HCV + alcohol (*n* = 23) NASH (*n* = 27) Other (*n* = 18)	NA		NA		NA	HE	16S rRNA	α: NA β: UniFrac	8	
Bajaj et al., 2016 [29]	HCV	76		27		2	Ascites/HE	16S rRNA	α: NA β: UniFrac	9	
Chen et al., 2016 [11]	HBV (*n* = 24) PBC (*n* = 6)	27		2		1	NA	16S rRNA V1-V3	α: Observed species, Chao1, Shannon, Simpson β: UniFrac, PLS-DA	7	
Wei et al., 2016 [45]	NA	5		5		-	NA	16S rRNA V1-V3	α: Chao1, Faith’s PD β: UniFrac	7	
Lee et al., 2023 [46]	HBV (*n* = 13) HCV (*n* = 4) Alcohol (*n* = 5)	14		7		0	Ascites	16S rRNA V3-V4	α: Shannon, Faith’s PD β: UniFrac	7	
Heidrich et al., 2018 [12]	HCV	NA		NA		NA	NA	16S rRNA V1-V2	α: Shannon, Simpson, Pielou β: Bray‒Curtis	7	
Bajaj et al., 2018 [47]	HCV (*n* = 65) Alcohol (*n* = 34) HCV + alcohol (*n* = 17) NASH (*n* = 46) Other (*n* = 40)	NA		NA		NA	Ascites/HE/variceal bleeding	16S rRNA V1-V2	α: Shannon β: UniFrac	9	
Bajaj et al., 2017 [48]	HCV (*n* = 49) Alcohol (*n* = 26) HCV + alcohol (*n* = 23) NASH (*n* = 29) Other (*n* = 16)	NA		NA		NA	HE	16S rRNA V1-V2 ITS1	α: Shannon β: NA	7	
Bajaj et al., 2018 [49]	HCV (*n* = 46) Alcohol (*n* = 39) HCV + alcohol (*n* = 18) NASH (*n* = 28) Other (*n* = 16)	NA		NA		NA	HE	16S rRNA	α: NA β: NA	6	
Inoue et al., 2018 [50]	HCV	NA		NA		NA	None	16S rRNA V1-V2	α: Shannon β: Bray‒Curtis, UniFrac	8	
Liu et al., 2018 [51]	NA	31		5		0	NA	16S rRNA V3-V4	α: Shannon, ACE β: Bray‒Curtis	6	
Ponziani et al., 2018 [52]	HCV	12		0		0	Varices	16S rRNA V3-V4	α: Chao1; β: UniFrac	7	
Shao et al., 2018 [53]	HBV (*n* = 76) Alcohol (*n* = 28) Other (*n* = 16)	NA		NA		NA	Ascites/HE	Metagenomics	α: Shannon, Simpson; β: Bray‒Curtis	7	
Sun et al., 2018 [54]	HBV	16		4		0	NA	16S rRNA V3-V5	α: Shannon, Chao1, ICEs; β: UniFrac	6	
Caussy et al., 2019 [9]	NAFLD	NA		NA		NA	NA	16S rRNA V4	α: Faith’s PD β: UniFrac	6	
Deng et al., 2019 [55]	HBV	30		31		19	NA	16S rRNA V4	α: Shannon, Chao1, Simpson β: Bray‒Curtis	6	
Jin et al., 2019 [56]	HCV (*n* = 2) Alcohol (*n* = 10) NAFLD (*n* = 1) Others (*n* = 4)	6		9		2	Ascites/HE/variceal bleeding	16S rRNA V5-V6	α: Observed species, Shannon β: UniFrac, Bray‒Curtis	9	
Zheng et al., 2020 [57]	NA	24		0		0	NA	16S rRNA V4	α: Observed species, Shannon, Simpson, ACE, Chao1, Fisher β: UniFrac, NMDS	9	
Sung et al., 2019 [58]	HBV (*n* = 21) HCV (*n* = 8) Alcohol (*n* = 11)	18		10		7	HE	16S rRNA V3-V4 metagenomics	α: Shannon, Chao1 β: Bray‒Curtis, NMDS	6	
Astbury et al., 2020 [59]	NASH	NA	NA		NA		NA	16S rRNA V4	α: Shannon, Observed species; β: UniFrac	6	
Bajaj et al., 2020 [30]	HCV (*n* = 46) Alcohol (*n* = 39) HCV + alcohol (*n* = 18) NASH (*n* = 28) Other (*n* = 16)	NA	NA		NA		Ascites/HE	16S rRNA V1-V2	α: Shannon β: NA	8	
Chen et al., 2020 [60]	HBV	NA	NA		NA		NA	16S rRNA V3-V4	α: Shannon, Simpson, Chao1, Pielou β: Bray‒Curtis	7	
Cox et al., 2020 [61]	HCV (*n* = 65) Alcohol (*n* = 34) HCV + alcohol (*n* = 17) NASH (*n* = 46) HBV (*n* = 27) Other (*n* = 13)	NA	NA		NA		Ascites/HE/varices/variceal bleeding	16S rRNA	α: Shannon; β: NA	9	
Lapidot et al., 2020 [25]	HCV (*n* = 35) NAFLD (33)	NA	NA		NA		NA	16S rRNA V4	α: Shannon, Faith’s PD, Observed species, Pielou β: UniFrac	8	
Oh et al., 2020 [62]	NAFLD	NA	NA		NA		NA	Metagenomics	α: Inverse Simpson; β: UniFrac	9	
Sydor et al., 2020 [63]	NASH	NA	NA		NA		HCC	16S rRNA	α: Shannon, Simpson, Observed species; β: NA	7	
Yang et al., 2020 [64]	HBV	NA	NA		NA		NA	16S rRNA V4	α: Chao1, Shannon, Simpson, Observed species β: UniFrac	6	
Zeng et al., 2020 [65]	HBV	NA	NA		NA		NA	16S rRNA V3-V4	α: ACE, Chao1, Shannon, Simpson β: UniFrac	6	
Huan et al., 2021 [66]	NA	NA	NA		NA		NA	16S rRNA V3-V4	α: Observed species, Chao1, Shannon, Simpson β: UniFrac	6	
Ponziani et al., 2021 [67]	HCV (*n* = 20) Alcohol (*n* = 8) HBV (*n* = 8) NAFLD (*n* = 14)	48	2		0		NA	16S rRNA V3-V4	α: Chao1 β: UniFrac	8	
Ren et al., 2021 [68]	HCV (*n* = 19) Alcohol (*n* = 9) HBV (*n* = 27) Other (*n* = 5)	NA	NA		NA		Ascites	Metagenomics	α: Shannon β: NA	7	
Alvares-da-Silva et al., 2022 [69]	HCV (*n* = 80) Alcohol (*n* = 80) NAFLD (*n* = 97) Others (*n* = 23)	NA	NA		NA		Ascites/HE/variceal bleeding	16S rRNA V1-V2	α: Shannon β: Bray‒Curtis	9	
Baltazar-Díaz et al., 2022 [70]	Alcohol	NA	NA		NA		Ascites/HE/UGIB	16S rRNA V3-V4	α: Shannon, Chao1, Observed species β: UniFrac	8	
Hua et al., 2022 [71]	HBV	NA	NA		NA		HE	16S rRNA V4	α: NA β: NA	6	
Maslennikov et al., 2022 [72]	HCV (*n* = 5) PBC (*n* = 4) PSC (*n* = 2) Alcohol (*n* = 15)	14	21		11		Ascites/HE/oesophageal varices	16S rRNA V3-V4	α: Shannon, Chao1, ACE, Simpson β: NA	7	
Shu et al., 2022 [73]	HBV	18	32				NA	16S rRNA V3-V4	α: Shannon, Chao1, ACE, Simpson β: UniFrac		
Sun et al., 2022 [74]	HBV	8	19		3		Ascites	16S rRNA V3-V4	α: Chao1, ACE β: Jaccard	8	
Ullah et al., 2022 [75]	HCV	NA	NA		NA		NA	16S rRNA V3-V4	α: Shannon, Chao1, Simpson β: Bray‒Curtis	7	
Zhou et al., 2022 [76]	NA	NA	NA		NA		Ascites	16S rRNA V3-V4	α: Chao1, Simpson, Shannon, Observed species β: UniFrac	8	
Chen et al., 2023 [77]	NA	NA	NA		NA		None	16S rRNA V3-V4	α: Observed species, ACE, Chao1, Shannon, Simpson, Shannoneven, Simpsoneven, and Faith’s PD β: UniFrac	7	
Lai et al., 2023 [78]	HCV (*n* = 4) HBV (*n* = 11) Others (*n* = 3)	NA	NA		NA		NA	16S rRNA V3-V4	α: ACE, Chao1, Shannon, Simpson β: UniFrac, Bray‒Curtis	7	
Wang et al., 2023 [28]	HBV (*n* = 14) HCV (*n* = 1) Alcohol (*n* = 8) HBV + alcohol (*n* = 3) HCV + alcohol (*n* = 1) Other (*n* = 3)	9	7		14		Ascites/HE/variceal bleeding	16S rRNA V4	α: Chao1, Shannon, Simpson, Pielou β: UniFrac, Bray‒Curtis	8	
Wu et al., 2023 [79]	NA	20	6		2		NA	16S rRNA V3-V4	α: Chao1, Shannon, Simpson, ACE β: UniFrac	8	
Yan et al., 2023 [80]	HBV	8	17		5		NA	16S rRNA 18S rRNA ITS	α: Chao1, Shannon, Simpson, ACE, PD whole tree, Observed species β: UniFrac	7	
Zhang et al., 2023 [81]	HBV	NA	NA		NA		NA	16S rRNA V3-V4	α: Chao1, Shannon, Simpson, ACE β: Bray‒Curtis	5	
Efremova et al., 2024 [82]	HBV (*n* = 3) HCV (*n* = 8) Alcohol (*n* = 23) Metabolic-associated fatty liver disease (*n* = 3) Mixed (*n* = 7) Cryptogenic (*n* = 3)	NA	NA		NA		Ascites/HE/oesophageal varices	16S rRNA	α: Chao1, Shannon, ACE; β: NA	5	

Abbreviations: LC, liver cirrhosis; HC, healthy control; HBV, hepatitis B virus; HCV, hepatitis C virus; NOS, Newcastle‒Ottawa Scale; 16 S rRNA, 16 S ribosomal RNA; PCR, polymerase chain reaction; AIH, autoimmune hepatitis; PBC, primary biliary cirrhosis; PSC, primary sclerosing cholangitis; HE, hepatic encephalopathy; UGIB, upper gastrointestinal bleeding; NASH, nonalcoholic steatohepatitis; NAFLD, nonalcoholic fatty liver disease

### Bacterial diversity

A total of 38 studies provided data on α diversity (Table 1), which was then assessed using six indices, namely, observed species (*n* = 19,423 patients and 468 controls), Shannon (*n* = 56,2092 patients and 1811 controls), Chao1 (*n* = 29,723 patients and 634 controls), Simpson (*n* = 20,541 patients and 480 controls), faith phylogenetic diversity (PD, *n* = 5,123 patients and 142 controls), and ACE (*n* = 13,320 patients and 273 controls) indices.

**Fig. 2:**
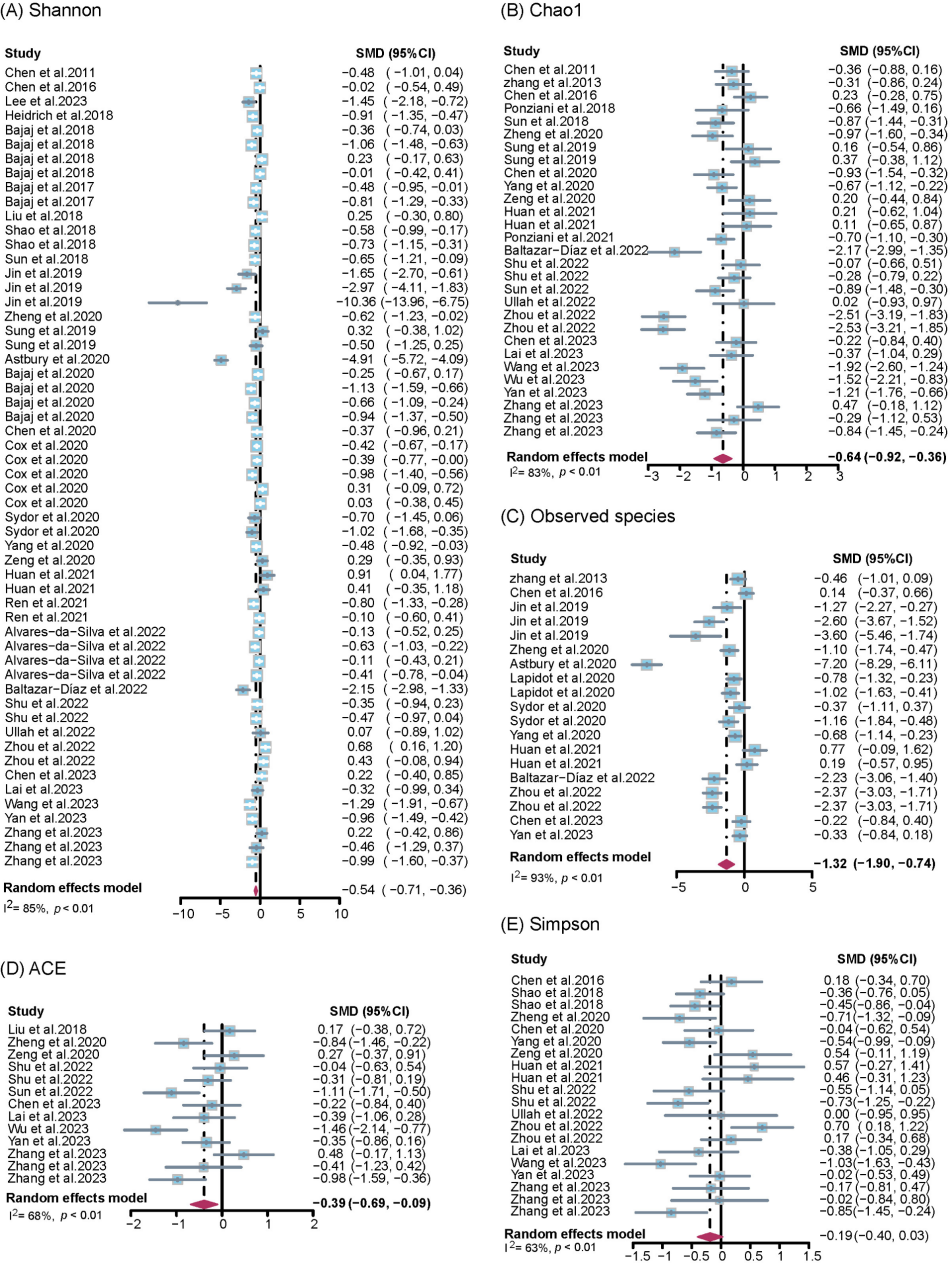
Forest plots comparing alpha diversity between patients with liver cirrhosis and healthy controls. This figure shows the overall analysis. For each study, the estimate of differences in the mean alpha diversity index and its 95% confidence interval (95% CI) was plotted with a diamond. SMD, standard mean difference; *I*^*2*^, I-square heterogeneity statistic

The pooled estimates revealed a moderately significant reduction in gut microbiome richness in patients with liver cirrhosis compared with controls, particularly for the Shannon (SMD = − 0.54, 95% CI = − 0.71 to − 0.36, *P* < 0.001), Chao1 (SMD = − 0.64, 95% CI = − 0.92 to − 0.36, *P* < 0.001), observed species (SMD = − 1.32, 95% CI = − 1.90 to − 0.74, *P* < 0.001), ACE (SMD = − 0.39, 95% CI = − 0.69 to − 0.09, *P* = 0.010), and PD (SMD = − 0.59, 95% CI = − 0.86 to − 0.32, *P* < 0.001) indices, but no significant difference was observed for the Simpson index (SMD = − 0.19, 95% CI = − 0.40 to 0.03, *P* = 0.088) (Table 2; Fig. 2, and Supplementary Figure S7). The between-study heterogeneity was significant except for PD, with *I*^2^ values ranging from 63% (Simpson) to 93% (observed species).

**Table 2:** Total analysis, stratified analysis, and sensitivity analysis of alpha diversity

Summary	*N*	Patients/controls	SMD (95% CI)	I^2^, %	*P* for Z test
Shannon					
Total	56	2092/1811	−0.54 (− 0.71, − 0.36)	85.0	< 0.001
Aetiology					< 0.001
HBV	12	313/294	−0.47 (− 0.73, − 0.21)	57.9	0.000
HCV	2	46/59	−0.52 (− 1.45, 0.42)	69.8	0.280
Alcohol	1	18/18	−2.15 (− 2.98, − 1.33)	−	0.000
HBV + HCV	1	18/17	−0.32 (− 0.99, 0.34)	−	0.343
HCV + alcohol	3	17/51	−4.28 (− 7.14, − 1.42)	90.6	0.003
HBV + alcohol	3	130/118	−0.61 (− 0.87, − 0.35)	0.0	0.000
NAFLD/NASH	3	55/116	−2.20 (− 4.72, 0.32)	97.1	0.087
Mix	24	1319/1000	−0.43 (− 0.60, − 0.26)	73.8	0.000
Unknown	7	176/138	0.31 (− 0.04, 0.65)	54.7	0.084
Diagnosis					< 0.001
Mix	44	1848/1517	−0.50 (− 0.67, − 0.34)	80.2	0.000
Ultrasonography (Clinical tests)	8	161/162	−0.15 (− 0.59, 0.28)	70.2	0.493
Liver biopsy alone	1	25/76	−4.91 (− 5.72, − 4.09)	−	0.000
Unknown	3	58/56	−0.41 (− 1.16, 0.34)	71.8	0.283
Region					0.002
East	29	770/682	−0.29 (− 0.49, − 0.09)	70.1	0.005
West	27	1322/1129	−0.83 (− 1.11, − 0.55)	90.1	0.000
Size of sample					0.256
< 100	49	1517/1383	−0.49 (− 0.66, − 0.31)	80.2	0.000
≥ 100	7	575/428	−0.87 (− 1.49, − 0.24)	95.1	0.007
Method of sequencing					0.577
16S rRNA	51	1881/1631	−0.53 (− 0.73, − 0.34)	86.0	0.000
Shotgun metagenomic sequencing	4	154/154	−0.56 (− 0.85, − 0.27)	35.5	0.000
ITS1	1	57/26	−0.81 (− 1.29, − 0.33)	−	0.000
Stage					0.004
Compensated	11	576/464	−0.19 (− 0.38, − 0.00)	55.9	0.048
Decompensated	11	481/464	−0.62 (− 0.86, − 0.38)	69.8	0.000
Unknown	34	1035/883	−0.67 (− 0.97, − 0.38)	88.4	0.000
Sensitivity analysis					
Remove low-quality studies	50	2017/1704	−0.47 (− 0.65, − 0.30)	83.9	< 0.001
Remove a single study					
Minimum	55	2090/1794	−0.51 (− 0.68, − 0.34)	83.9	< 0.001
Maximum	55	2077/1802	−0.56 (− 0.73, − 0.38)	84.8	< 0.001
Trim and fill	60	−	−0.39 (− 0.60, − 0.18)	−	< 0.001
Chao1					
Total	29	723/634	−0.64 (− 0.92, − 0.36)	83.0	< 0.001
Aetiology					0.007
HBV	12	322/298	−0.44 (− 0.75, − 0.13)	70.3	0.005
HCV	2	20/21	−0.37 (− 1.03, 0.30)	11.7	0.278
Alcohol	1	18/18	−2.17 (− 2.99, − 1.35)	−	0.000
HBV + alcohol	1	36/24	−0.36 (− 0.88, 0.16)	−	0.174
HBV + HCV	1	18/17	−0.37 (− 1.04, 0.29)	−	0.272
Mix	5	141/122	−0.49 (− 1.18, 0.20)	85.1	0.161
Unknown	7	168/134	−1.07 (− 1.91, − 0.23)	90.4	0.013
Diagnosis					0.100
Mix	17	462/396	−0.88 (− 1.29, − 0.48)	87.0	0.000
Ultrasonography (Clinical tests)	8	153/132	−0.28 (− 0.69, 0.13)	63.6	0.184
Unknown	4	108/106	−0.37 (− 0.94, 0.20)	72.6	0.206
Region					0.243
East	26	643/554	−0.58 (− 0.88, − 0.29)	83.1	0.000
West	3	80/80	−1.14 (− 2.02, − 0.26)	80.7	0.011
Sensitivity analysis					
Remove low-quality studies	26	665/578	−0.69 (− 0.98, − 0.39)	83.3	< 0.001
Remove a single study					
Minimum	28	693/604	−0.57 (− 0.83, − 0.32)	79.3	< 0.001
Maximum	28	698/619	−0.68 (− 0.96, − 0.40)	82.0	< 0.001
Trim and fill	29	−	−0.64 (− 0.92, − 0.36)	−	< 0.001
Simpson					
Total	20	541/480	−0.19 (− 0.40, 0.03)	63.0	0.088
Aetiology					0.054
HBV	10	272/241	−0.24 (− 0.51, 0.03)	54.2	0.082
HCV	1	8/9	0.00 (− 0.95, 0.952)	−	1.000
HBV + alcohol	2	94/94	−0.40 (− 0.69, − 0.11)	0.0	0.006
HBV + HCV	1	18/17	−0.38 (− 1.05, 0.29)	−	0.264
Mix	1	30/20	−1.03 (− 1.63, − 0.43)	−	0.001
Unknown	5	119/99	0.23 (− 0.29, 0.74)	69.6	0.388
Diagnosis					0.092
Mix	13	415/375	−0.24 (− 0.51, − 0.02)	67.2	0.072
Ultrasonography (Clinical tests)	4	68/49	0.20 (− 0.17, 0.58)	0.0	0.282
Unknown	3	58/56	−0.39 (− 0.90, 0.12)	40.9	0.135
Method of sequencing					0.198
16S rRNA	18	447/386	−0.15 (− 0.40, 0.09)	65.9	0.220
Shotgun metagenomic sequencing	2	94/94	−0.40 (− 0.69, − 0.11)	0.0	0.006
Sensitivity analysis					
Remove low-quality studies	13	370/315	−0.26 (− 0.50, − 0.02)	56.5	0.032
Remove a single study					
Minimum	19	511/460	−0.14 (− 0.35, 0.06)	59.6	0.174
Maximum	19	511/450	−0.24 (− 0.44, − 0.05)	54.2	0.015
Trim and fill	23	−	−0.32 (− 0.55, − 0.08)	−	0.008
Observed species					
Total	19	423/468	−1.32 (− 1.90, − 0.74)	93.0	< 0.001
Aetiology					0.002
HBV	2	84/61	−0.53 (− 0.87, − 0.19)	1.6	0.002
Alcohol	1	18/18	−2.23 (− 3.06, − 1.40)	−	0.000
NAFLD/NASH	4	64/133	−2.81 (− 5.46, − 0.16)	97.3	0.038
HCV + alcohol	2	8/34	−2.30 (− 4.56, − 0.04)	78.5	0.046
Mix	4	109/104	−0.52 (− 1.02, -0.01)	69.4	0.044
Unknown	6	140/118	−0.87 (− 1.88, 0.15)	92.3	0.094
Diagnosis					< 0.001
Mix	16	363/373	−1.16 (− 1.58, − 0.73)	84.6	0.000
Liver biopsy alone	1	25/76	−7.20 (− 8.29, − 6.11)	−	0.000
Ultrasonography (Clinical tests)	2	35/19	0.44 (− 0.12, 1.01)	0.0	0.126
Region					0.018
East	10	280/233	−0.65(− 1.23, − 0.08)	89.0	0.026
West	9	143/235	−2.18 (− 3.30, − 1.06)	94.1	0.000
Sensitivity analysis					
Remove low-quality studies	16	406/417	−1.16 (− 1.78, − 0.54)	93.5	< 0.001
Remove a single study					
Minimum	18	398/392	−0.99 (− 1.41, − 0.57)	85.8	< 0.001
Maximum	18	408/459	−1.44 (− 2.02, − 0.85)	92.7	< 0.001
Trim and fill	21	−	−0.91 (− 1.59, − 0.22)	−	0.009
ACE					
Total	13	320/273	−0.39 (− 0.69, − 0.09)	67.5	0.010
Aetiology					0.806
HBV	8	193/181	−0.31 (− 0.68, 0.06)	66.4	0.101
HBV + HCV	1	18/17	−0.39 (− 1.06, 0.28)	−	0.249
Unknown	4	109/75	−0.57 (− 1.26, 0.12)	80.2	0.107
Diagnosis					0.080
Mix	9	232/197	−0.34 (− 0.65, − 0.02)	61.2	0.037
Ultrasonography (Clinical tests)	1	30/20	−1.11 (− 1.71, − 0.50)	−	0.000
Unknown	3	58/56	−0.30 (− 1.20, 0.60)	80.4	0.511
Sensitivity analysis					
Remove low-quality studies	10	262/217	−0.41 (− 0.74, − 0.09)	66.1	0.012
Remove a single study					
Minimum	12	292/257	−0.31 (− 0.58, − 0.04)	58.9	0.026
Maximum	12	295/258	−0.46 (− 0.75, − 0.17)	63.1	0.002
Trim and fill	13	−	−0.39 (− 0.69, − 0.09)	−	0.010

Abbreviations: LC, liver cirrhosis; HBV, hepatitis B virus; HCV, hepatitis C virus; NASH, nonalcoholic steatohepatitis; NAFLD, nonalcoholic fatty liver disease

We further conducted subgroup analyses according to the methods used for diagnosis, aetiology of liver cirrhosis, region where the study was conducted, sample size, sequencing method, and disease stage (Table 2). No significant interactions were detected across the different subgroups with the Simpson and ACE indices (*P* > 0.05), whereas significant interactions were detected across the subgroups in terms of aetiology, diagnosis, region, and stage with the Shannon index. Significant interactions were observed between aetiology and the Chao1 (*P* = 0.007) and observed species (*P* = 0.002) indices. The factors of diagnosis and region were also significantly associated with the observed species index (*P* < 0.05).

**Fig. 3:**
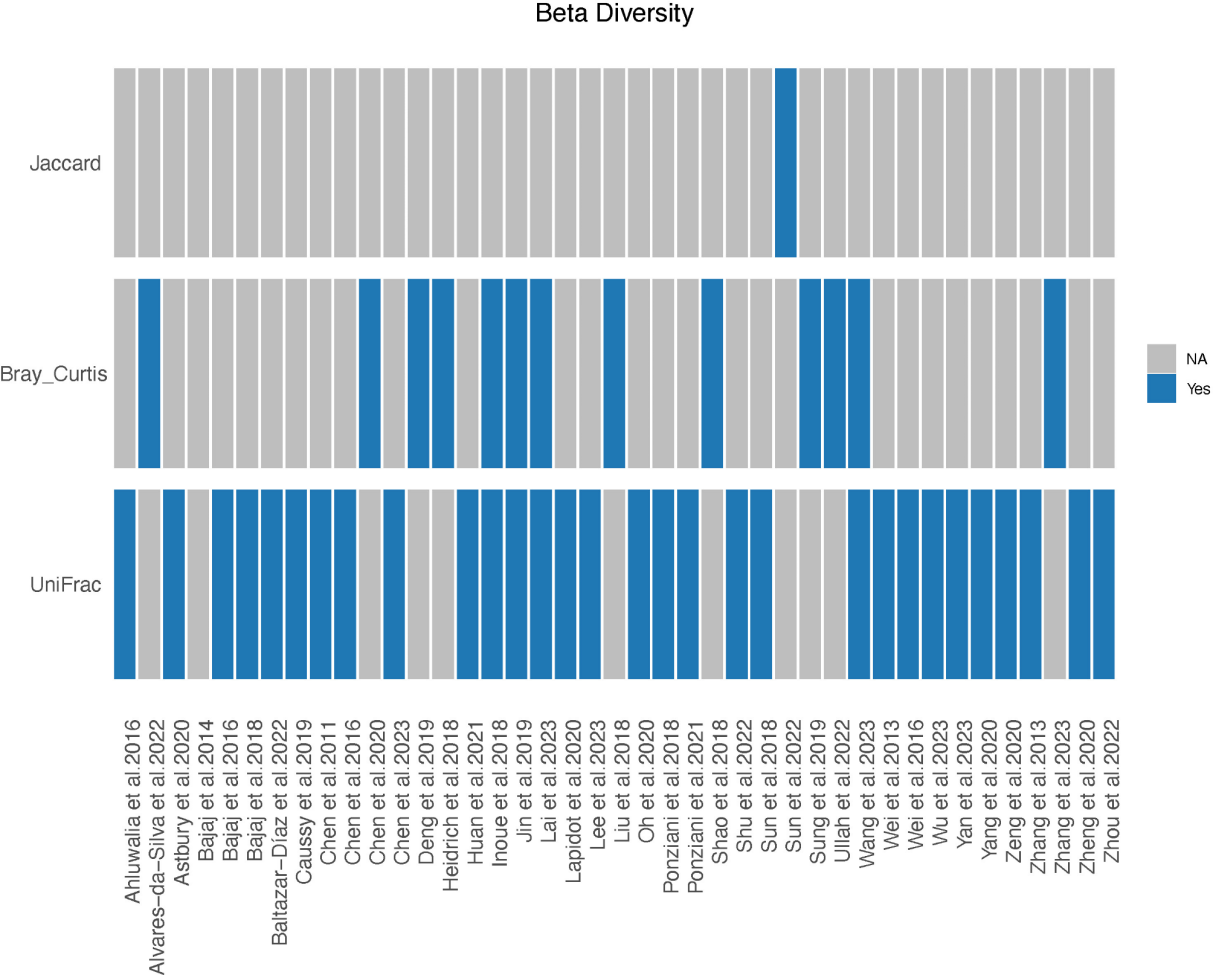
Comparison of beta diversity between patients with liver cirrhosis and healthy controls

A total of 41 studies provided data on β diversity (Table 1). Among these, 26 studies employed either weighted or unweighted UniFrac methods for the distance calculation, 9 studies used the Bray–Curtis method, and 4 studies utilized both (Fig. 3 and Supplementary Table S6). Over 75% (36 of 41) of the studies reported significant differences in β diversity, whereas the remaining 5 studies reported no significant differences.

### Microbiota changes

A total of 46 studies examined the relative abundance of gut microbes in patients with liver cirrhosis compared with controls at the phylum, class, family, or genus level. In Supplementary Figures S9–S12, we summarize the microbes reported in at least two studies. The changes in 10 phyla, 8 classes, 36 families and 78 genera are summarized in Supplementary Figures S14–17. A high degree of inconsistency at the phylum, family, and genus levels was noted across the studies, indicating limited evidence for disease-specific changes in the relative abundance of gut microbes. At the family level, we observed some consistency: depletion of Clostridiales XIV (4/4) and enrichment of Enterobacteriaceae (17/19) and Fusobacteriaceae (3/3). At the genus level, three microbes (*Bilophila* (5/5), *Subdoligranulum* (4/5), and *Alistipes* (4/4)) were depleted; the relative abundances of *Veillonella* (13/15), *Streptococcus* (12/13), and *Haemophilus* (8/10)) were enriched; and *Oscillospira* (3/3) was not significantly different.

The CDR for patients with liver cirrhosis was lower compared to controls, with no significant difference observed (SMD = − 0.36, 95% CI = − 0.77 to 0.05, *P* = 0.087) (Supplementary Figures S18). We also performed a meta-analysis of the reported microbial taxa at the phylum, class, family, and genus levels (Supplementary Figures S19–22). At the phylum level, a significant enrichment of Proteobacteria (SMD = 1.02, 95% CI = 0.57 to 1.47, *P* < 0.001) was observed, with a significant depletion of Firmicutes (SMD = − 1.07, 95% CI = − 1.97 to − 0.17, *P* = 0.019), whereas no significant difference was observed in the abundance of Bacteroidetes (SMD = 0.48, 95% CI = − 0.28 to 1.24, *P* = 0.218). At the class level, a significant enrichment of Bacillli (SMD = 1.64, 95% CI = 0.76 to 2.52, *P* < 0.001) was observed, with a significant depletion of Clostridia (SMD = − 2.02, 95% CI = − 3.08 to − 0.97, *P* < 0.001). At the family level, significant enrichment was detected for Enterobacteriaceae (SMD = 1.35, 95% CI = 0.03 to 2.67, *P* = 0.044), Streptococcaceae (SMD = 1.00, 95% CI = 0.51 to 1.48, *P* < 0.001), and Pasteurellaceae (SMD = 0.49, 95% CI = 0.09 to 0.89, *P* = 0.016); a significant depletion was detected for Porphyromonadaceae (SMD = − 0.56, 95% CI = − 0.98 to − 0.13, *P* = 0.109), Clostridiales XIV (SMD = − 1.29, 95% CI = − 1.63 to − 0.94, *P* < 0.001), Lachnospiraceae (SMD = − 1.73, 95% CI = − 2.42 to − 1.04, *P* < 0.001), and Ruminococcaceae (SMD = − 1.34, 95% CI = − 1.73 to − 0.95, *P* < 0.001); and nonsignificant changes in the abundances of Bacteroidaceae, Veillonellaceae, and Prevotellaceae (all *P* values > 0.05). At the genus level, *Ruminococcus*, *Turicibacter*, and *Lachnoclostridium* showed nonsignificant changes. *Dorea* (SMD = − 1.43, 95% CI = − 2.29 to − 0.56, *P* = 0.001), *Faecalibacterium* (SMD = − 1.24, 95% CI = − 2.34 to − 0.14, *P* = 0.027), and *Bacteroides* (SMD = − 1.45, 95% CI = − 2.65 to − 0.26, *P* = 0.017) were depleted, whereas *Haemophilus* (SMD = 0.49, 95% CI = 0.10 to 0.89, *P* = 0.015), *Streptococcus* (SMD = 1.09, 95% CI = 0.84 to 1.34, *P* < 0.001) and *Veillonella* (SMD = 0.99, 95% CI = 0.74 to 1.24, *P* < 0.001) were enriched.

### Sensitivity analysis and analysis of publication bias

Sensitivity analyses were performed by removing low-quality studies (score < 6) (Table 2), and all α diversity indices remained significantly lower in patients with liver cirrhosis than in controls. Egger’s test revealed no significant publication bias, and the *P* values were 0.775, 0.290, 0.569, and 0.568 for the Chao1, Simpson, Faith’s PD, and ACE indices, respectively. However, publication bias was observed for the Shannon index (*P* = 0.019) and observed species index (*P* = 0.022) (Supplementary Table S8). The funnel plots of the Shannon and observed species indices indicated possible publication bias (Supplementary Figure S13). After imputing missing studies for the Shannon and observing species indices using the trim and fill method, the recalculated pooled SMDs were not substantially different from the initial estimates.

## Discussion

Through its hepatic portal and bile secretion system, the liver interacts directly with the gut. An imbalance in the intestinal flora is believed to play a key role in the progression of liver cirrhosis, especially the translocation of bacteria through the gut epithelial barrier [17–19]. Previous studies have limitations that can reduce the validity of their findings, including the small sample size of the articles and the lack of use of new sequencing technologies. Thus, different studies cannot draw consistent conclusions about microbiota biomarkers. Therefore, we conducted this study to provide reliable evidence-based results regarding the association between the microbiota abundance and liver cirrhosis. A total of 53 eligible papers including 5076 participants were included in the present study. Our findings indicated that liver cirrhosis patients had a lower α diversity of the gut microbiota than healthy individuals did. Most studies reported notable differences in β diversity in a qualitative manner. The differentially abundant bacteria varied significantly among the included studies. However, they still presented consistent characteristics: depletion of beneficial microbes (i.e., *Alistipes* and *Subdoligranulum*) and enrichment of potentially harmful microbes (i.e., *Veillonella*, *Haemophilus*, and *Streptococcus*). As a result, these findings highlight the characteristics of dysbiotic gut microbiota in patients with liver cirrhosis.

Only one systematic review has summarized the alterations in the gut microbiome in patients with liver cirrhosis. The results from the study by Huang et al. indicated that, compared with healthy controls, patients with liver cirrhosis presented increased abundances of *Enterobacter* and *Enterococcus*, but decreased abundances of *Lactobacillus* and *Bifidobacterium* [20]. In the present study, we included nearly three times as many articles and utilized a greater amount of available data to achieve more reliable and comprehensive conclusions.

Alpha diversity and β diversity are widely used to reflect microbial communities. Our results revealed a moderate reduction in some α diversity indices and changes in β diversity, which were consistent with the findings of most of the included studies. Earlier research provided substantial evidence of the mechanisms linking dysbiosis to liver cirrhosis [21]. First, the integrity of the gut barrier is compromised because of the reduction in tight junction proteins and damage to the intestinal mucosa. This increased gut permeability facilitates the translocation of gut bacteria and their products (e.g., endotoxins) into the portal system, exacerbating liver inflammation and damage [22]. Second, changes in the microbiome result in reduced levels of short-chain fatty acids (SCFAs), particularly butyrate, which play crucial roles in maintaining gut epithelial health and modulating systemic metabolism [23]. Additionally, the shift in the microbial composition affects bile acid metabolism or increases ammonia production, further burdening the liver [24].

Our study revealed that gut microbes were associated with the liver cirrhosis risk, which is consistent with findings from previous studies [6, 25, 26]. The alterations in the gut microbiota of patients with liver cirrhosis were characterized by an overgrowth of potentially pathogenic, potent endotoxin-producing bacteria (i.e., *Haemophilus*, *Streptococcus*, and *Veillonella*) and a decrease in the levels of potentially beneficial bacteria, specifically butyrate-producing bacteria (i.e., *Alistipes* and *Subdoligranulum*). In liver cirrhosis, harmful facultative anaerobes such as Proteobacteria, along with the family Streptococcaceae and the class Bacilli (including the genus *Streptococcus*), increased. Their ability to survive in oxygenated environments made them more likely to undergo bacterial translocation, leading to liver and systemic inflammation. In contrast, beneficial anaerobes such as Clostridia, particularly the families Lachnospiraceae and Ruminococcaceae, were less involved in bacterial translocation, allowing them to maintain gut health and immune homeostasis. These shifts may result in a decrease in the levels of anti-inflammatory short-chain fatty acids (SCFAs) and may enhance leaky gut and gut dysbiosis. Moreover, a reduction in SCFA-producing bacteria has been linked to liver inflammation and steatosis in patients with liver cirrhosis [27, 28]. Most studies found an increase in dysbiosis, with a lower CDR reflecting a stronger negative impact—characterized by a reduction in beneficial bacteria, an increase in potential pathogens, and a close association with inflammation, elevated endotoxin levels, and disease progression. Our findings are consistent with this, but the lack of statistical significance is likely due to the limited number of extractable data points from the studies [29–31]. Enterobacteriaceae and *Streptococcus* are the most common pathogens responsible for bacterial infections in individuals with cirrhosis. *Veillonella* is a genus of gram-negative, anaerobic bacteria commonly found in the oral and gastrointestinal tracts. Among the studies included here, Jin et al. reported that *Veillonella parvula*, when administered to mice with liver cirrhosis, reversed the ameliorative effects of splenectomy by increasing the levels of gut-derived endotoxins in the liver, which primarily target the Tlr4/Nlrp3 pathway [32]. Further animal research is needed to elucidate the specific mechanisms involved in the relationship between bacterial strains and liver cirrhosis.

As cirrhosis progresses, complications such as HE, hepatocellular carcinoma (HCC), and cirrhotic portal hypertension (CPH) significantly impact patient survival, with CPH being a major driving force behind gastroesophageal variceal bleeding. Studies highlighted gut microbiota dysbiosis as a key factor in cirrhosis progression and its complications, suggesting potential microbiota-based biomarkers [31, 33–35]. Understanding these associations could reveal mechanisms and guide new therapeutic strategies to improve outcomes. A recent study by Sharma et al. found that monitoring specific gut microbes (e.g., E*nterococcus faecium* and *Staphylococcus aureus*) is critical for HE prognosis, while increases in *Alistipes putredinis*, *Bacteroides eggerthii*, and *Prevotella_uc* may predict HCC [33]. Another study found that Clostridium cluster IV levels are significantly lower in patients with CPH [36]. This cluster includes many butyric acid-producing strains, such as Ruminococcaceae, which has been reported to mitigate liver cirrhosis, and *F. prausnitzii*, known for its anti-inflammatory properties. Notably, butyric acid-producing bacteria and other SCFA-producing microbes have been shown to reduce the progression of liver cirrhosis and NASH [37, 38]. In experimental models, administering mixed strains of Clostridium cluster IV to mice significantly increased colonic regulatory T cells, suggesting an immunomodulatory mechanism underlying their protective effects [39, 40]. These insights emphasize the potential of noninvasive microbial biomarkers for the early detection of decompensation in patients with cirrhosis.

16S rRNA gene sequencing has been the predominant method for examining the composition of the gut microbiota. However, this technique is limited in its ability to identify major taxa and explore microbial diversity and cannot pinpoint specific microbial species and strains. Therefore, more metagenomic studies are needed to provide a deeper, more detailed understanding of the gut microbiota. We performed a comprehensive search, and most of the studies lacked detailed raw data on bacterial abundance. Better designed studies and detailed data are needed to fully understand how the microbiota plays a role in cirrhosis pathogenesis. Some limitations, however, must be considered. First, different ethnicities, foods and drinks, cigarette smoking, medication use, and places of residence may influence the gut microbiota, which may explain the inconsistent results among different studies. Second, larger studies with larger sample sizes are needed to verify these results, which should explore liver damage according to the severity of each cause (HBV, HCV, etc.). Most pooled taxon analyses included only a small to moderate number of included studies, suggesting that some of our analyses may not have sufficient statistical power. Due to the limited number of included studies, we could not conduct a pooled analysis according to the stages of liver cirrhosis and treatment strategies. A cautious approach must therefore be taken when attempting to determine a correlation between the gut microbiota and clinical therapy. Third, our analysis included only the bacterial microbiota, but viruses and fungi are also key components of the gut microbial community. Fourth, most included studies were based on a case–control study design. More cohort studies are needed to establish the causal relationship between gut dysbiosis and liver cirrhosis risk. Finally, since the detection of the microbiota was not repeated, we were unable to determine the changes in the microbial community during the development of liver cirrhosis. Multicentre studies of multiple ethnic groups must be conducted to ensure that the sample size is large enough, that confounders are controlled and that the results are reliable.

In conclusion, this meta-analysis indicated that patients with liver cirrhosis had lower microbiota richness than healthy controls did. Specifically, Enterobacteriaceae, Pasteurellaceae, Streptococcaceae, Bacilli, and *Streptococcus* were enriched, whereas Lachnospiraceae, Ruminococcaceae, and Clostridia were depleted.

## Electronic supplementary material

Below is the link to the electronic supplementary material.

Supplementary Material 1

## Data Availability

All data generated or analyzed during this study are included in this published article and its supplementary information files.
