# Supplementary material for: Associations between changes in the gut microbiota and liver cirrhosis: a systematic review and meta-analysis
Source: BMC Gastroenterol. 2025 Jan 13;25:16. doi: 10.1186/s12876-025-03589-5 (PMC11727502; doi:10.1186/s12876-025-03589-5)
Supplement: Supplementary file 1 — Supplementary Material 1 [file 12876_2025_3589_MOESM1_ESM.pdf]

Supplementary Materials

Supplementary Table S1. Systematic search details in PubMed, Cochrane Library, Embase, and Web of Science.

Supplementary Table S2. Quality assessment of the included case-control studies using the Newcastle- Ottawa Scale.

Supplementary Table S3. Quality assessment of the included cohort studies using the Newcastle-Ottawa Scale.

Supplementary Table S4. List of excluded experimental studies with reasons for exclusion.

Supplementary Table S5. Methodology of stool processing, sequencing and other details of the included studies.

Supplementary Table S6. Methodology and findings of the included studies assessing beta diversity for the patient vs. control group comparison.

Supplementary Figure S7. Forest plots for alpha diversity about Faith's PD.

Supplementary Table S8. Publication bias tests (Egger's test) for alpha diversity.

Supplementary Table S9. Summary of findings for each taxon reported at phylum level.

Supplementary Table S10. Summary of findings for each taxon reported at class level.

Supplementary Table S11. Summary of findings for each taxon reported at family level.

Supplementary Table S12. Summary of findings for each taxon reported at genus level.

Supplementary Figure S13. Funnel plots for assessing publication bias.

Supplementary Figure S14. Changes of relative abundance of 10 phyla included in analysis.

Supplementary Figure S15. Changes of relative abundance of 8 classes included in analysis.

Supplementary Figure S16. Changes of relative abundance of 36 families included in analysis.

Supplementary Figure S17. Changes of relative abundance of 78 genera included in analysis.

Supplementary Figure S18. A meta-analysis of the cirrhosis dysbiosis ratio.

Supplementary Figure S19. A meta-analysis of the reported microbial taxa at phylum level.

Supplementary Figure S20. A meta-analysis of the reported microbial taxa at class level.

Supplementary Figure S21. A meta-analysis of the reported microbial taxa at family level.

Supplementary Figure S22. A meta-analysis of the reported microbial taxa at genus level.

Supplementary Table S1. Literature search strategy.

1.Pubmed

Search number	Query	Results
#1	"Gastrointestinal Microbiome"[Mesh]	41,996
#2	((((((((((((((((((((((((((((((((((((((Gastrointestinal Microbiome[Title/Abstract]) OR (Gastrointestinal Microbiomes[Title/Abstract])) OR (Microbiome, Gastrointestinal[Title/Abstract])) OR (Gut Microbiome[Title/Abstract])) OR (Gut Microbiomes[Title/Abstract])) OR (Microbiome, Gut[Title/Abstract])) OR (Gut Microflora[Title/Abstract])) OR (Microflora, Gut[Title/Abstract])) OR (Gut Microbiota[Title/Abstract])) OR (Gut Microbiotas[Title/Abstract])) OR (Microbiota, Gut[Title/Abstract])) OR (Gastrointestinal Flora[Title/Abstract])) OR (Flora, Gastrointestinal[Title/Abstract])) OR (Gut Flora[Title/Abstract])) OR (Flora, Gut[Title/Abstract])) OR (Gastrointestinal Microbiota[Title/Abstract])) OR (Gastrointestinal Microbiotas[Title/Abstract])) OR (Microbiota, Gastrointestinal[Title/Abstract])) OR (Gastrointestinal Microbial Community[Title/Abstract])) OR (Gastrointestinal Microbial Communities[Title/Abstract])) OR (Microbial Community, Gastrointestinal[Title/Abstract])) OR (Gastrointestinal Microflora[Title/Abstract])) OR (Microflora, Gastrointestinal[Title/Abstract])) OR (Gastric Microbiome[Title/Abstract])) OR (Gastric Microbiomes[Title/Abstract])) OR (Microbiome, Gastric[Title/Abstract])) OR (Intestinal Microbiome[Title/Abstract])) OR (Intestinal Microbiomes[Title/Abstract])) OR (Microbiome, Intestinal[Title/Abstract])) OR (Intestinal Microbiota[Title/Abstract])) OR (Intestinal Microbiotas[Title/Abstract])) OR (Microbiota, Intestinal[Title/Abstract])) OR (Intestinal Microflora[Title/Abstract])) OR (Microflora, Intestinal[Title/Abstract])) OR (Intestinal Flora[Title/Abstract])) OR (Flora, Intestinal[Title/Abstract])) OR (Enteric Bacteria[Title/Abstract])) OR (Bacteria, Enteric[Title/Abstract])) OR ("Gastrointestinal Microbiome"[Mesh])	95,074
#3	"Liver Cirrhosis"[Mesh]	104,610
#4	(((((((Liver Cirrhosis[Title/Abstract]) OR (Hepatic Cirrhosis[Title/Abstract])) OR (Cirrhosis, Hepatic[Title/Abstract])) OR (Cirrhosis, Liver[Title/Abstract])) OR (Fibrosis, Liver[Title/Abstract])) OR (Liver Fibrosis[Title/Abstract])) OR (Cirrhosis[Title/Abstract])) OR ("Liver Cirrhosis"[Mesh])	161,006
#5	("2010/01/01"[Date - Create]: "2024/03/10"[Date - Create])	17,550,302
#6	#2 AND #4 AND #5	1327

2.Cochrane

Search number	Query	Results
#1	MeSH descriptor: [Gastrointestinal Microbiome] explode all trees	2,611

#2	(Gastrointestinal Microbiome):ti,ab,kw OR (Gastrointestinal Microbiomes):ti,ab,kw OR (Microbiome, Gastrointestinal):ti,ab,kw OR (Gut Microbiome):ti,ab,kw OR (Gut Microbiomes):ti,ab,kw	3,868
#3	(Microbiome, Gut):ti,ab,kw OR (Gut Microflora):ti,ab,kw OR (Microflora, Gut):ti,ab,kw OR (Gut Microbiota):ti,ab,kw OR (Gut Microbiotas):ti,ab,kw	6,298
#4	(Microbiota, Gut):ti,ab,kw OR (Gastrointestinal Flora):ti,ab,kw OR (Flora, Gastrointestinal):ti,ab,kw OR (Gut Flora):ti,ab,kw OR (Flora, Gut):ti,ab,kw	5,856
#5	(Gastrointestinal Microbiota):ti,ab,kw OR (Gastrointestinal Microbiotas):ti,ab,kw OR (Microbiota, Gastrointestinal):ti,ab,kw OR (Gastrointestinal Microbial Community):ti,ab,kw OR (Gastrointestinal Microbial Communities):ti,ab,kw	2,703
#6	(Microbial Community, Gastrointestinal):ti,ab,kw OR (Gastrointestinal Microflora):ti,ab,kw OR (Microflora, Gastrointestinal):ti,ab,kw OR (Gastric Microbiome):ti,ab,kw OR (Gastric Microbiomes):ti,ab,kw	1,001
#7	(Microbiome, Gastric):ti,ab,kw OR (Intestinal Microbiome):ti,ab,kw OR (Intestinal Microbiomes):ti,ab,kw OR (Microbiome, Intestinal):ti,ab,kw OR (Intestinal Microbiota):ti,ab,kw	3,065
#8	(Intestinal Microbiotas):ti,ab,kw OR (Microbiota, Intestinal):ti,ab,kw OR (Intestinal Microflora):ti,ab,kw OR (Microflora, Intestinal):ti,ab,kw OR (Intestinal Flora):ti,ab,kw	4,155
#9	(Flora, Intestinal):ti,ab,kw OR (Enteric Bacteria):ti,ab,kw OR (Bacteria, Enteric):ti,ab,kw	2,582
#10	#1 OR #2 OR #3 OR #4 OR #5 OR #6 OR #7 OR #8 OR #9	10,288
#11	MeSH descriptor: [Liver Cirrhosis] explode all trees	11,076
#12	(Liver Cirrhosis):ti,ab,kw OR (Hepatic Cirrhosis):ti,ab,kw OR (Cirrhosis, Hepatic):ti,ab,kw OR (Cirrhosis, Liver):ti,ab,kw OR (Fibrosis, Liver):ti,ab,kw	12,729
#13	(Liver Fibrosis):ti,ab,kw OR (Cirrhosis):ti,ab,kw	14,054
#14	#11 OR #12 OR #13	14,318
#15	#10 AND #14<br>with Publication Year from 2010 to 2024	272

3.Embase

Search number	Query	Results
#1	'intestine flora'/exp	107,366
#2	'intestine flora':ti,ab,kw OR 'alimentary canal flora':ti,ab,kw OR 'alimentary tract flora':ti,ab,kw OR 'bowel flora':ti,ab,kw OR 'bowel microbiota':ti,ab,kw OR 'digestive canal flora':ti,ab,kw OR 'digestive tract flora':ti,ab,kw OR 'enteric flora':ti,ab,kw OR 'enteric microbiota':ti,ab,kw OR 'flora, intestine':ti,ab,kw OR 'gastro intestinal flora':ti,ab,kw OR 'gastrointestinal canal flora':ti,ab,kw OR 'gastrointestinal flora':ti,ab,kw OR 'gastrointestinal microbiome':ti,ab,kw OR 'gastrointestinal microbiota':ti,ab,kw OR 'gastrointestinal tract flora':ti,ab,kw OR 'gastrointestine flora':ti,ab,kw OR 'gastrointestine tract flora':ti,ab,kw OR 'gut bacteria':ti,ab,kw OR 'gut microbiota':ti,ab,kw OR 'intestinal bacteria':ti,ab,kw OR 'intestinal bacterial flora':ti,ab,kw OR 'intestinal bacterium':ti,ab,kw OR 'intestinal canal flora':ti,ab,kw OR 'intestinal flora':ti,ab,kw OR 'intestinal microbe':ti,ab,kw OR 'intestinal microbes':ti,ab,kw OR 'intestinal microbiota':ti,ab,kw OR 'intestinal microflora':ti,ab,kw OR 'intestinal microorganism':ti,ab,kw OR 'intestinal tract	87,145

	flora':ti,ab,kw OR 'intestine bacteria':ti,ab,kw OR 'intestine bacteria change':ti,ab,kw OR 'intestine bacterial flora':ti,ab,kw OR 'intestine bacterium':ti,ab,kw OR 'intestine microbial flora':ti,ab,kw OR 'intestine microflora':ti,ab,kw	
#3	#1 OR #2	126,963
#4	'liver cirrhosis'/exp	212,742
#5	'liver cirrhosis':ti,ab,kw OR 'hepatic cirrhosis':ti,ab,kw OR 'cirrhosis, hepatic':ti,ab,kw OR 'cirrhosis, liver':ti,ab,kw OR 'fibrosis, liver':ti,ab,kw OR 'liver fibrosis':ti,ab,kw OR cirrhosis:ti,ab,kw	209,422
#6	#4 OR #5	274,194
#7	[2010-2024]/py	22,407,565
#8	#3 AND #6 AND #7	2661

4.Web of science

Search number	Query	Results
#1	Gastrointestinal Microbiome (Topic) or Gastrointestinal Microbiomes (Topic) or Microbiome, Gastrointestinal (Topic) or Gut Microbiome (Topic) or Gut Microbiomes (Topic) or Microbiome, Gut (Topic) or Gut Microflora (Topic) or Microflora, Gut (Topic) or Gut Microbiota (Topic) or Gut Microbiotas (Topic) or Microbiota, Gut (Topic) or Gastrointestinal Flora (Topic) or Flora, Gastrointestinal (Topic) or Gut Flora (Topic) or Flora, Gut (Topic) or Gastrointestinal Microbiota (Topic) or Gastrointestinal Microbiotas (Topic) or Microbiota, Gastrointestinal (Topic) or Gastrointestinal Microbial Community (Topic) or Gastrointestinal Microbial Communities (Topic) or Microbial Community, Gastrointestinal (Topic) or Gastrointestinal Microflora (Topic) or Microflora, Gastrointestinal (Topic) or Gastric Microbiome (Topic) or Gastric Microbiomes (Topic) or Microbiome, Gastric (Topic) or Intestinal Microbiome (Topic) or Intestinal Microbiomes (Topic) or Microbiome, Intestinal (Topic) or Intestinal Microbiota (Topic) or Intestinal Microbiotas (Topic) or Microbiota, Intestinal (Topic) or Intestinal Microflora (Topic) or Microflora, Intestinal (Topic) or Intestinal Flora (Topic) or Flora, Intestinal (Topic) or Enteric Bacteria (Topic) or Bacteria, Enteric (Topic)	35,863
#2	Liver Cirrhosis (Topic) or Hepatic Cirrhosis (Topic) or Cirrhosis, Hepatic (Topic) or Cirrhosis, Liver (Topic) or Fibrosis, Liver (Topic) or Liver Fibrosis (Topic) or Cirrhosis (Topic)	278,739
#3	2010-01-01/2024-03-10 (Publication Date)	95,788,662
#4	#1 AND #2 AND #3	173

Supplementary Table S2. Quality assessment of the included cross-sectional and case-control studies using the Newcastle-Ottawa Scale.

Case-control studies	Selection			Comparability		Exposure			Total
	Case definition	Representativeness of the case	Selection of controls	Definition of controls	Based on design and analysis	Assessment of exposure	Same measurement for case and controls	Non-respondents	0-9
Chen et al.2011 [1]	1	1	1	1	1	0	1	1	7
Lu et al.2011 [2]	1	1	1	1	1	0	1	1	7
Zhang et al.2013 [3]	1	1	1	1	1	0	1	1	7
Wei et al.2013 [4]	1	1	1	1	1	1	1	1	8
Bajaj et al.2014 [5]	1	1	1	1	2	1	1	1	9
Qin et al.2014 [6]	1	1	1	1	1	1	1	1	8
Tuomisto et al.2014 [7]	1	1	1	1	1	0	1	1	7
Ahluwalia et al.2016 [8]	1	1	1	1	1	1	1	1	8
Bajaj et al.2016 [9]	1	1	1	1	2	1	1	1	9
Chen et al.2016 [10]	1	1	1	0	1	1	1	1	7
Wei et al.2016 [11]	1	1	1	1	1	1	1	0	7
Lee et al.2023 [12]	1	1	1	0	1	1	1	1	7
Heidrich et al.2018 [13]	1	1	1	1	0	1	1	1	7
Bajaj et al.2018 [14]	1	1	1	1	2	1	1	1	9
Bajaj et al.2017 [15]	1	1	1	1	1	1	1	0	7
Bajaj et al.2018 [16]	1	0	1	0	2	1	1	0	6
Inoue et al.2018 [17]	1	1	1	1	1	1	1	1	8
Liu et al.2018 [18]	1	1	1	0	1	1	1	0	6
Ponziani et al.2018 [19]	1	1	1	0	1	1	1	1	7
Shao et al.2018 [20]	1	1	1	0	1	1	1	1	7
Sun et al.2018 [21]	1	1	1	0	1	1	1	0	6
Caussy et al.2019 [22]	1	1	1	0	1	1	1	0	6
Deng et al.2019 [23]	0	1	1	0	1	1	1	1	6
Jin et al.2019 [24]	1	1	1	1	2	1	1	1	9
Zheng et al.2020 [25]	1	1	1	1	2	1	1	1	9
Sung et al.2019 [26]	1	1	1	0	1	1	1	0	6
Astbury et al.2020 [27]	1	1	1	0	1	1	1	0	6
Bajaj et al.2020 [28]	0	1	1	1	2	1	1	1	8
Cox et al.2020 [29]	1	1	1	1	2	1	1	1	9
Lapidot et al.2020 [30]	1	1	1	1	1	1	1	1	8
Oh et al.2020 [31]	1	1	1	1	2	1	1	1	9
Sydor et al.2020 [32]	0	1	1	0	2	1	1	1	7
Yang et al.2020 [33]	1	1	1	0	1	1	1	0	6

Zeng et al.2020 [34]	**1**	**1**	**1**	**0**	**1**	**1**	**1**	**0**	**6**
Huan et al.2021 [35]	**0**	**1**	**1**	**0**	**2**	**1**	**1**	**0**	**6**
Ponziani et al.2021 [36]	**1**	**1**	**1**	**0**	**2**	**1**	**1**	**1**	**8**
Ren et al.2021 [37]	**1**	**1**	**1**	**1**	**1**	**1**	**1**	**0**	**7**
Alvares-da-Silva et al.2022 [38]	**1**	**1**	**1**	**1**	**2**	**1**	**1**	**1**	**9**
Baltazar-Díaz et al.2022 [39]	**1**	**1**	**1**	**1**	**1**	**1**	**1**	**1**	**8**
Hua et al.2022 [40]	**1**	**1**	**1**	**0**	**1**	**1**	**1**	**0**	**6**
Maslennikov et al.2022 [41]	**1**	**1**	**1**	**0**	**2**	**1**	**1**	**0**	**7**
Shu et al.2022 [42]	**1**	**1**	**1**	**0**	**1**	**1**	**1**	**0**	**6**
Sun et al.2022 [43]	**1**	**1**	**1**	**1**	**1**	**1**	**1**	**1**	**8**
Ullah et al.2022 [44]	**1**	**1**	**1**	**1**	**1**	**1**	**1**	**0**	**7**
Zhou et al.2022 [45]	**1**	**1**	**1**	**0**	**2**	**1**	**1**	**1**	**8**
Chen et al.2023 [46]	**1**	**1**	**1**	**0**	**1**	**1**	**1**	**1**	**7**
Lai et al.2023 [47]	**1**	**1**	**1**	**0**	**1**	**1**	**1**	**1**	**7**
Wang et al.2023 [48]	**1**	**1**	**1**	**1**	**1**	**1**	**1**	**1**	**8**
Wu et al.2023 [49]	**1**	**1**	**1**	**0**	**2**	**1**	**1**	**1**	**8**
Yan et al.2023 [50]	**1**	**1**	**1**	**1**	**0**	**1**	**1**	**1**	**7**
Zhang et al.2023 [51]	**1**	**1**	**1**	**0**	**0**	**1**	**1**	**0**	**5**
Efremova et al.2024 [52]	**1**	**1**	**1**	**0**	**0**	**1**	**1**	**0**	**5**

Supplementary Table S3. Quality assessment of the included cohort studies using the Newcastle-Ottawa Scale.

Cohort study	Selection				Comparability	Outcome			Total
	Representativeness of the exposed cohort	Selection of the non-exposed cohort	Ascertainment of exposure	Demonstration that outcome of interest was not present at start of study	Comparability of cohorts on the basis of the design or analysis	Assessment of outcome	Was follow-up long enough for outcomes to occur	Adequacy of follow up of cohorts	0-9
Chen et al.2020 [53]	1	1	1	1	1	1	0	1	7

Supplementary Table S4. List of excluded experimental studies with reasons for exclusion.

Study	Reasons for exclusion
Li et al.2010 [54]	Not a study type of interest
Bajaj et al.2012 [55]	Not a study type of interest
Bajaj et al.2015 [56]	Not a study type of interest
Bajaj et al.2015 [57]	Not a study type of interest
Flass et al.2015 [58]	Not a study type of interest
Ponziani et al.2021 [59]	Not a study type of interest
Cao et al.2023 [60]	Did not perform gut microbiota analysis nor reported diversity
Höppner et al.2023 [61]	Did not perform gut microbiota analysis nor reported diversity
Bajaj et al.2012 [62]	No adequate data for synthesis
Liu et al.2012 [63]	No adequate data for synthesis
Mou et al.2018 [64]	No adequate data for synthesis
Cox et al.2022 [65]	No adequate data for synthesis
Chen et al.2011 [66]	No adequate data for synthesis
Aliwa et al.2022 [67]	No adequate data for synthesis
Ciocan et al.2018 [68]	No adequate data for synthesis
Usami et al.2013 [69]	Lack of control group
Grat et al.2016 [70]	Lack of control group
Jacobs et al.2018 [71]	Lack of control group
Bajaj et al.2019 [72]	Lack of control group
Bajaj et al.2019 [73]	Lack of control group
Bajaj et al.2022 [74]	Lack of control group
Bajaj et al.2022 [75]	Lack of control group
Bajaj et al.2022 [76]	Lack of control group
Haraguchi et al.2019 [77]	Lack of control group
Lang et al.2020 [78]	Lack of control group
Bajaj et al.2021 [79]	Lack of control group
Shen et al.2021 [80]	Lack of control group
Wellhoner et al.2021	Lack of control group
Lin et al.2023 [81]	Liver cirrhosis in advanced liver disease
Naseri et al.2021 [82]	Lack of control group
Luo et al.2022 [83]	Lack of control group
Maslennikov et al.2022 [84]	Lack of control group
Naseri et al.2022 [85]	Lack of control group
Bajaj et al.2023 [86]	Lack of control group

Effenberger et al.2023 [87]	Lack of control group
Huang et al.2023 [88]	Lack of control group
Jinato et al.2023 [89]	Lack of control group
Chang et al.2019 [90]	Controls with a history of mild liver fibrosis
Wellhoner et al.2021 [91]	Achieved sustained virological response

Supplementary Table S5. Methodology and findings of the included studies assessing beta diversity for the patient vs. control group comparison.

Study	Selection criteria	Definition of LC	Sequencing	Storage	DNA extraction method
Chen et al.2011 [1]	**Included criteria:** Cirrhosis was diagnosed histologically in 21 of 36 (58%) patients, and clinically and radiologically in the remaining 15 (42%) patients, in whom biopsy was contraindicated due to uncontrolled coagulapathy and/or uncontrolled ascites. None of these patients had comorbid diseases. All healthy subjects had normal liver biochemistry tests without evidence of hepatic or other diseases.<br>**Exclusion criteria:** a history of antibiotic or probiotic treatment within the previous 8 weeks of fecal sample collection.	Presence of LC confirmed by liver histology (58%). Clinically and radiologically in the remaining 15 (42%) patients, in whom biopsy was contraindicated.	Pyrosequencing 16S rRNA V3 (Genome Sequencer FLX System (Roche))	-70°C	QIAamp DNA Stool Mini Kit (Qiagen, Valencia, CA)
Lu et al.2011 [2]	**Included criteria:** None of them had undergone any GI surgery or received any antibiotics, prokinetic drugs or lactulose for 3 weeks prior to the study. Patients with decompensated HBV cirrhosis: HBsAg positive, HBeAg positive, and HBV DNA detectable with cirrhosis, based on liver biopsy or obvious findings on ultrasound imaging, but without GI bleeding. Healthy controls: without any GI or liver disease.<br>**Exclusion criteria:** Patients were excluded if they did not satisfy the following conditions: (1) stool frequency once daily, with well-	Presence of LC confirmed by liver histology and ultrasonography.	qPCR for 16S rRNA	-75°C	Qiagen Stool Kit (Qiagen, Hilden, Germany)

	formed stools weighing 350–450 g; (2) normal fecal calprotectin and lactoferrin levels (<50 μg/g feces); and (3) all volunteers lived in Hangzhou and maintained their habitual diet, typical of the Hangzhou region (China) in the month before the trial.				
Wei et al.2013 [4]	**Included criteria:** Cirrhosis was diagnosed histologically in all patients. None of the patients had comorbid diseases. All healthy individuals had normal liver biochemistry test results with no evidence of hepatic or other diseases. **Exclusion criteria:** the subjects had received antibiotics, probiotics, steroids or other hormones (including oral, intramuscular or intravenous injection) for at least 3 months before sampling.	Cirrhosis was diagnosed histologically in all patients.	Metagenomic sequencing (using Illumina/Solexa System)	-80°C	NA
Zhang et al.2013 [3]	**Included criteria:** Liver cirrhosis was diagnosed on the basis of medical history, clinical and radiological observations, and results of laboratory tests. Alcohol-related cirrhosis patients who had been abstaining from alcohol for at least 2 months were included. All healthy subjects were found to have normal liver biochemistry without evidence of hepatic or other diseases. Otherwise, the health status of the healthy volunteers was self-reported. No volunteers indicated that they had suffered any diseases of the gastrointestinal tract or any other	Liver cirrhosis was diagnosed on the basis of medical history, clinical and radiological observations, and results of laboratory tests. .	Pyrosequencing for 16S rRNA V2 (454 Life Sciences Genome Sequencer FLX instrument (Roche))	-70°C	QIAamp DNA Stool Mini Kit (Qiagen)

	metabolic diseases such as obesity, diabetes, and cardiovascular disease. None had been subjected to surgical procedures for several years before this study. None had taken antibiotics or probiotics within the previous 3 months of fecal sample collection. **Exclusion criteria:** the presence or history of overt HE; recent history within the previous 3 months of factors that may have influenced gut flora and circulating endotoxin and ammonia levels (e.g., infection, treatment with antibiotics or immunosuppressants or beta-blocker proton-pump inhibitors , sodium, lactulose or sedative intake, and gastrointestinal bleeding); recent history of reversible hepatic functional decompensation, such as drug-related hepatotoxicity and choledocholithiasis (< 3 months); history of renal impairment, electrolyte imbalance, spontaneous bacterial peritonitis, systemic infl ammatory response syndrome, and hepatocellular carcinoma; history of transjugular intra hepatic portosystemic shunt or shunt surgery for portal hypertension; neurological or psychiatric disorders, such as depression, Alzheimer 's disease, or Parkinson 's disease; serious diseases such as diabetes, hypo glycemia, heart failure, chronic obstructive pulmonary disease, or renal failure; and inability to complete psychometric tests because of poor vision or illiteracy.				
Bajaj et al.2014	**Included criteria:** enrolled patients	Cirrhosis was diagnosed	Multi-tagged	-80°C	NA

[5]	with cirrhosis (diagnosed histologically, endoscopic/radiological evidence or signs of decompensation) after informed consent. All cirrhotic patients underwent blood draw for MELD score and endotoxin (using published techniques). Subsequently enrolled age-matched healthy controls that were free of liver disease and were not on any medications apart from non-steroidal analgesics or antihypertensives.<br>**Exclusion criteria:** patients with an unclear cirrhosis diagnosis, other end-organ disease prior to admission, hospitalized for >48 h before enrollment, or transferred from another hospital.	histologically, endoscopic/radiological evidence or signs of decompensation in all patients.	pyrosequencing		
Qin et al.2014 [6]	**Included criteria:** patients who met the diagnostic criteria of LC. The liver imaging and liver biochemistry results of all healthy controls were in the normal range. Physical examination, routine examination of blood, urine and stools, preoperative serological tests (including the detection of hepatitis B surface antigen, hepatitis C virus antibody, Treponema pallidum antibody, human immunodeficiency virus antibody), liver function, renal function, electrolyte, liver ultrasound, electrocardiogram and chest X-ray results were checked in the healthy controls to exclude any abnormal samples.<br>**Exclusion criteria:** for the control	Liver cirrhosis was diagnosed according to the international guidelines by comprehensive consideration of liver biopsy, imaging examination, clinical symptoms, physical signs, laboratory tests, medical history, progress notes and cirrhosis-associated complications.	Metagenomic sequencing (using Illumina System)	-80°C	Phenol trichloromethane DNA extraction

	group included hypertension, diabetes, obesity,metabolic syndrome, IBD, nonalcoholic fatty liver disease, coeliac disease and cancer. Individuals who received antibiotics and/or probiotics within 8 weeks before enrolment were also excluded. To confirm diagnoses, solicited outside expert opinions for each case. Borderline or otherwise inconclusive cases were excluded from the study. After discharge of the patient from the hospital, their case history was further reviewed for medication history. Cases that progressed to hepatic carcinoma or those found to suffer from other diseases such as hypertension and diabetes were excluded.				
Tuomisto et al.2014 [7]	**Included criteria:** out-of-hospital death, male sex, age over 18 years, time elapsed post mortem 5 days or less, time interval between death and storage of the body in the mortuary less than 24 hours, intact middle torso and bowel, no signs of bacterial infections or visible wounds or necrosis and no signs or reports of drug addiction. None of the study subjects were reported to have taken antibiotics for 2 weeks prior to sampling. The hospital records of the autopsy cases were available, and were scrutinized for mentions of alcohol use. The criterion for the definition of alcoholism or heavy alcohol consumption was a comment in the hospital records/police reports or alcoholism-related microscopic	Cirrhosis was diagnosed histologically and through imaging examination in all patients.	qRT-PCR	-80°C	Zymo Fecal DNA Kit (Zymo Research Corporation, Irvine, California, USA)

	findings such as presence of alcoholic liver disease or cerebellar atrophy, along with positive post-mortem alcohol test.				
Ahluwalia et al.2016 [8]	**Included criteria:** Cirrhosis was diagnosed using biopsy, history of frank decompensation (ascites, HE, variceal bleeding, jaundice) or varices in a patient with chronic liver disease or those with radiological features of cirrhosis. only included those with prior type C HE who were controlled on lactulose or rifaximin for at least 3 months with good adherence per clinic notes and interview before enrollment. Healthy controls were subjects without chronic diseases who were not receiving any regular medications.<br>**Exclusion criteria:** subjects who were unable to provide informed consent, those with a mini-mental status exam result < 25, those who had an uncertain diagnosis of cirrhosis, those with recent (< 6 months history) of alcohol or illicit drug misuse and those on absorbable antibiotics within the last 6 weeks.	Cirrhosis was diagnosed using biopsy, history of frank decompensation (ascites, HE, variceal bleeding, jaundice) or varices in a patient with chronic liver disease or those with radiological features of cirrhosis.	16S rRNA (GS-FLX pyrosequencer (Roche))	-80°C	NA
Bajaj et al.2016 [9]	**Included criteria:** diagnosed cirrhosis, healthy controls without chronic diseases were also enrolled using community advertisements and word-of-mouth. Similar dietary recall analyses were performed.<br>**Exclusion criteria:** subjects who were unable to give consent, those with concomitant aetiologies of cirrhosis (significant alcohol history, NAFLD	Cirrhosis was diagnosed using either biopsy, endoscopic features of varices in a patient with chronic liver disease or radiological evidence of cirrhosis and portal hypertension.	Multitag pyrosequencing	-80°C	NA

	on liver biopsy, hepatitis B, cholestatic disease or autoimmune markers), hepatocellular cancer, HIV infection, those on any antibiotics (including rifaximin) or probiotics and those who were actively drinking within the last 3 months. Patients who were currently on or had stopped HCV therapy within the last 12 months were excluded as were those with an sustained virological response (SVR) less than a year prior to enrolment.				
Chen et al.2016 [10]	**Included criteria:** All individuals underwent complete evaluation including biochemical tests of liver functions and ultrasongraphy. Cirrhosis was diagnosed on the basis of clinical and laboratory data supported by liver biopsy or ultrasongraphy. None of the patients had a history of hepatic encephalopathy. All the cirrhotic patients were detected with esophageal and gastric varices. No other antacid medicine, rifaximin, or lactulose, was used in the cirrhotic patients.<br>**Exclusion criteria:** they were on or had received antibiotics in the last 2 months.	Cirrhosis was diagnosed on the basis of clinical and laboratory data supported by liver biopsy or ultrasongraphy.	16S rRNA V1-V3 (454 GS FLX Titanium sequencer (Roche))	-80°C	QIAamp DNA isolation kit (Qiagen, Valencia, CA, USA)
Wei et al.2016 [11]	**Included criteria:** Cirrhosis was diagnosed by liver biopsy in all patients. None of these patients had comorbid diseases. All healthy individuals had normal liver biochemistry tests without evidence of hepatic or other diseases.<br>**Exclusion criteria:** a history of	Cirrhosis was diagnosed by liver biopsy.	16S rRNA V1-V3 (MiSeq platform (Illumina Inc., San Diego, CA, USA))	-80°C	QIAamp DNA Stool Mini Kit (Cat No: 51504)

	antibiotics, probiotics, steroids, or other hormones ingestion (including oral, intramuscular or intravenous) within the previous 3 months of stool sampling.				
Lee et al.2023 [12]	**Inclusion criteria:** Patients who have liver cirrhosis diagnosed by the image modalities of ultrasonography, computed tomography or magnetic resonance imaging together with impaired liver function, or by liver biopsy in Taipei Veterans General Hospital. **Exclusion criteria:** 1. Patients who are less than 20 years old. 2. Patients who have antecedent or active malignancy. 3. Patients who use lactulose, proton pump inhibitors, nonsteroidal anti-inflammatory drugs, antibiotics, probiotics, prebiotics within one month. 4. Patients with human immunodeficiency virus coinfection or severe comorbidities, such as chronic renal failure or uremia without regular dialysis, recent acute coronary syndrome or stroke, severe heart failure, and major trauma. 5. Patients who experience gastrointestinal operation in recent one month. 6. Patients who refuse to join the study or refuse to sign informed consent.	The diagnosis of cirrhosiswas made according to the ultrasonography, computed tomography, or magnetic resonance imaging together with impaired liver function, or by liver biopsy.	16S rRNA V3-V4 (MiSeq platform (Illumina Inc., San Diego, CA, USA))	-80°C	QIAamp DNA Stool Mini Kit (Qiagen, Hilden, Germany)
Heidrich et	**Inclusion criteria:** Healthy volunteers	Ultrasonographic	16S rRNA V1-	-80°C	TRIzol Reagent

al.2018 [13]	could enter the study when no known liver diseases were documented in their medical history.<br>**Exclusion criteria:** use of antibiotic within 3 months prior inclusion; Samples of patients after solid-organ transplantation and with sustained virological response; samples of individuals receiving antibiotics, immunosuppressive medications or L-ornithine-L-aspartate, respectively; drank excessively and patients reported the use of pre-and/or probiotics.	examination of the liver was carried out by a DEGUM II-III certified physician routinely in all patients. In this study, transient elastography (TE, Fibroscan®; Echosens, Paris, France) and Acoustic Radiation Force Impulse (ARFI) (Siemens Acuson S2000, Munich, Germany) elastography were used for assessment of liver fibrosis. Only procedures with at least 10 valid acquisitions, a success rate of >60%, and an interquartile range (IQR/M) <30% were defined as eligible for the study.	V2 (Illumina)		(Ambion) according to the manufacturer's instructions
Bajaj et al.2018 [14]	**Inclusion criteria:** All healthy controls were free of chronic diseases, were not on any prescription medications and had normal liver function.<br>**Exclusion criteria:** Patients with alcohol abuse and illicit drug use (defined by DSM-V criteria), unable to provide samples and those who were hospitalized within the last 30 days	Patients were diagnosed with cirrhosis based on liver biopsy if available, elastography or clinical, biochemical and radiological evidence of cirrhosis. Decompensation was defined as prior variceal bleeding, ascites under treatment, hepatic encephalopathy under treatment and those with jaundice.	16S rRNA V1-V2 (PGM Ion Torrent Nextgeneration sequencer)	-80°C	NA

Bajaj et al.2017 [15]	**Inclusion criteria:** Healthy controls were outpatients without any chronic diseases or medications. **Exclusion criteria:** Patients who were unable to give consent or provide stool within 48 hours of admission, with alcohol misuse, alcoholic hepatitis, on antifungal medications and with gastrointestinal bleeding episodes within 6 weeks; undergoing HCV eradication or those on probiotics within the last 3 months.	cirrhosis diagnosed using liver biopsy, evidence of frank decompensation or radiological features.	16S rRNA V1-V2 and ITS1(PGM Ion Torrent Nextgeneration sequencer)	-80°C	NA
Bajaj et al.2018 [16]	**Inclusion criteria:** cirrhosis patients **Exclusion criteria:** NA	cirrhosis (defined through biopsy, features of decompensation, endoscopic or radiological evidence of varices or cirrhosis in the setting of chronic liver disease)	16S rRNA (Ion Torrent)	-80°C	FastDNA Spin kit for Soil (MP Biomedicals)
Inoue et al.2018 [17]	**Inclusion criteria:** (a) older than 18 years of age, (b) Asian race, (c) positive serum HCV antibody (anti-HCV) and detectable serum HCV RNA (>1.2 log10 IU/mL). The exclusion criteria were as follows: (a) coinfection with hepatitis B virus (HBV) and/or human immunodeficiency virus (HIV), or other secondary organism (bacteria and fungi) infection; (b) evidence of liver disease because of other etiology; (c) use of hepatotoxic drugs; (d) HCV antiviral therapy; (e) individuals diagnosed with malignancies, except HCC, or who underwent prior anti-cancer treatment; (f) consumption of	LC patients were comprehensively diagnosed according to (a) laboratory tests such as low platelet counts; (b) FIB-4 index > 3.25; (c) Fibroscan ≥ 12.5 kPa; (d) evidence of LC (portal hypertension, including splenomegaly, ascites, esophageal varix and/or stomach varix) based on magnetic resonance elastography, transient elastography, liver biopsy and other imaging tests	16S rRNA V1-V2 (MiSeq paired-end sequencing system, Illumina Inc., San Diego, California)	-80°C	bead-beating method followed by phenol extraction.

	unhealthy commodities (including drug abuse); (g) hospitalization longer than 3 days; (h) medical history as liver transplant recipients; (i) individuals with severe cytopenias, renal failure, heart failure, and pregnant or breastfeeding women.<br>Healthy individuals as follows: (a) older than 50 years of age and (b) Asian race. The exclusion criteria were as follows: (a) infection with HCV, HBV and/or HIV, or other secondary organism (bacteria and fungi) infection; (b) evidence of liver disease and diabetes mellitus; (c) use of hepatotoxic drugs; (d) past history of liver disease; (e) consumption of unhealthy commodities including drugs; (f) hospitalization longer than 3 days; (g) prescription of lactulose, antibiotics including rifaximin, histamine H2-receptor antagonists (H2B), beta2 adrenergic receptor agonists (β2B), ursodeoxycholic acid (UDCA), and probiotics (lactose-fermenting Lactobacilli and Bifidobacteria) within 6 months prior the sampling; and (h) individuals with decompensated cirrhosis, severe cytopenias, renal failure, heart failure, and pregnant or breastfeeding women.	including MRI, CT and/or ultrasonography; (e) complications associated with portal hypertension.			
Liu et al.2018 [18]	**Inclusion criteria:** Diagnosed with LC; Participants taking any medication (including lactulose) affecting gastrointestinal motility were asked to stop the medication at least 3 days before the SBT studies. All the patients denied receiving any	diagnosed with LC by comprehensively reviewing the results of liver biopsies, imaging and laboratory tests in addition to clinical	16S rRNA V3-V4 (Illumina HiSeq 2500 platform, United States)	-80°C	E.Z.N.A.® stool DNA Kits (Omega Bio-Tek, Norcross, GA, United States)

	antibiotics during previous 3 months. **Exclusion criteria:** (a) Patients with progression to hepatic carcinoma, uncontrolled ascites or encephalopathy. (b) presence of malignancy, infections, known GI or renal disease or significant respiratory or cardiac dysfunction; (c) diagnosis of diabetes mellitus, untreated thyroid dysfunction or previous gastrointestinal surgery; (d) history of an autoimmune disease such as multiple sclerosis, rheumatoid arthritis, IBS and IBD.	symptoms, physical signs, medical history, progress notes and associated complications			
Ponziani et al.2018 [19]	Inclusion criteria: Healthy controls from the family members of patients were also included in the study and matched for sex and age with the study population. Exclusion criteria: Patients with past or present clinical history of excessive alcohol intake, concomitant diagnosis of non-alcoholic fatty liver disease (NAFLD), chronic hepatitis B virus (HBV) infection, HIV infection, autoimmune or cholestatic liver disease, diabetes, obesity or intestinal diseases, previous diagnosis of hepatocellular carcinoma (HCC) or other tumours, as well as treatment with antibiotics, prebiotics, probiotics, non-absorbable disaccharides, laxatives, ursodeoxycholic acid, or proton pump inhibitors during the last 3 months before the enrolment and throughout the study period	Cirrhosis was confirmed by histological, radiologic, elastosonographic, and/or clinical findings.	16S rRNA V3-V4 (Illumina MiSeq platform, San Diego, CA, USA)	-80°C	QIAmp Fast DNA Stool mini kit (Qiagen, Germany)
Shao et al.2018	**Included criteria:** patients who met	Liver cirrhosis was	Metagenomic	-80°C	Phenol

[20]	the diagnostic criteria of LC. The liver imaging and liver biochemistry results of all healthy controls were in the normal range. Physical examination, routine examination of blood, urine and stools, preoperative serological tests (including the detection of hepatitis B surface antigen, hepatitis C virus antibody, Treponema pallidum antibody, human immunodeficiency virus antibody), liver function, renal function, electrolyte, liver ultrasound, electrocardiogram and chest X-ray results were checked in the healthy controls to exclude any abnormal samples.<br>**Exclusion criteria:** for the control group included hypertension, diabetes, obesity,metabolic syndrome, IBD, nonalcoholic fatty liver disease, coeliac disease and cancer. Individuals who received antibiotics and/or probiotics within 8 weeks before enrolment were also excluded. To confirm diagnoses, solicited outside expert opinions for each case. Borderline or otherwise inconclusive cases were excluded from the study. After discharge of the patient from the hospital, their case history was further reviewed for medication history. Cases that progressed to hepatic carcinoma or those found to suffer from other diseases such as hypertension and diabetes were excluded.	diagnosed according to the international guidelines by comprehensive consideration of liver biopsy, imaging examination, clinical symptoms, physical signs, laboratory tests, medical history, progress notes and cirrhosis-associated complications.	sequencing (using Illumina System)		trichloromethane DNA extraction
Sun et al.2018 [21]	**Inclusion criteria:** age of 30-70 years, normal renal function, and body mass	LC was diagnosed by the	16S rRNA V3-	-80°C	NA

	index (BMI) >20. **Exclusion criteria:** (a) severe complications; and/or (b) another disease, such as cancer, intestinal disease, hypertension or metabolic disease. (c) the healthy volunteers included hypertension, diabetes, obesity, metabolic syndrome, inflammatory bowel disease (IBD), non-alcoholic fatty liver disease, coeliac disease and LC. (d) All participants who received antibiotics and/ or probiotics within 8 weeks before enrolment.	comprehensive integration of imaging findings, clinical symptoms and physical signs, laboratory test results and medical history.	V5 (Illumina MiSeq platform at Majorbio Bio-Pharm Technology Co., Ltd. (Shanghai, China))		
Caussy et al.2019 [22]	**Inclusion criteria:** Probands with NAFLD-cirrhosis had a documented evidence of NAFLD with either biopsy-proven or meeting imaging criteria for cirrhosis. **Exclusion criteria:** regular and excessive alcohol consumption within 2 years of recruitment (≥14 drinks/week for men or ≥7 drinks/week for women); use of hepatotoxic drugs or drugs known to cause hepatic steatosis; evidence of liver diseases other than NAFLD, including viral hepatitis (detected with positive serum hepatitis B surface antigen or hepatitis C viral RNA), Wilson's disease, hemochromatosis, alpha-1 antitrypsin deficiency, autoimmune hepatitis, and cholestatic or vascular liver disease; clinical or laboratory evidence of chronic illnesses associated with hepatic steatosis, including human	biopsy-proven or meeting imaging criteria for cirrhosis; a liver stiffness cut point of >3.63 kPa on MRE	16S rRNA V4 (Illumina MiSeq)	-80°C	Qiagen MagAttract PowerSoil DNA kit

	immunodeficiency virus infection (HIV), celiac disease, cystic fibrosis, lipodystrophy, dysbetalipoproteinemia, and glycogen storage diseases; evidence of active substance abuse, significant systemic illnesses, contraindication(s) to MRI, pregnant or trying to become pregnant, or any other condition which, in the investigator's opinion, may affect the patient's competence or compliance in completing the study.				
Deng et al.2019 [23]	**Inclusion criteria:** patients did not receive probiotics and antibiotics for 8 weeks prior to admission. Alcohol history and consumption (white liquor) of all patients was asked.<br>**Exclusion criteria:** Patients with solid organ transplantation, HCC, drug-induced liver injury, autoimmune liver disease and alcoholic fatty liver; patients with specific food habits, like vegetarians and lactose lovers; Use of antibiotics within 8 weeks prior sample collection.	NA	16S rRNA V4 (Illumina MiSeq platform, San Diego, CA, USA)	-80°C	MoBioPowersoil DNA extraction kits (MoBio, Carlsbad, CA, USA)
Jin et al.2019 [24]	**Inclusion criteria:** patients with cirrhosis were: (a) Diagnosis of cirrhosis by biopsy, or by clinical criteria with imaging confirmation. (b) Ability to provide a stool sample within 2 hours of evacuation. (c) Age between 18 to 80 years. (d) Ability for providing informed consent. healthy controls 1) BMI of 25-34.5 kg/m^2. 2) Men and pre-menopausal, non-pregnant or non-lactating women. 3) No diagnosis of gastrointestinal (GI)	Diagnosis of cirrhosis by biopsy, or by clinical criteria with imaging confirmation.	16S rRNA V5-V6 (Illumina MiSeq platform)	-80°C	QIAamp DNA Stool Mini Kit

	disorders. 4) No history of GI surgical intervention. 5) No history of diabetes. 6) No known allergies or intolerances to fiber sources (e.g. celiac disease). 7) Weight stable (± 3%) for ≥ 1 month. 8) Non-vegetarian. 9) Non-smoking. 10) Alcohol intake ≤ 8 drinks/week. 11) Exercise < 2 h/week. 12) No use of supplements (including pre- and probiotics). 13) No antibiotic treatment in the last 3 months. 14) No use of anti-hypertensive, lipid-lowering, anti-diabetic, anti-inflammatory (i.e. corticosteroids or chronic nonsteroidal anti-inflammatory drugs), or laxative medications.<br>**Exclusion criteria:** (a) Recent gastrointestinal bleeding within 7 days of the study. (b) Active infection on current systemic antibiotic treatment (oral antibiotics for the treatment of hepatic encephalopathy or for prophylaxis of spontaneous bacterial peritonitis were allowed). (c) Current active alcohol use.				
Zheng et al.2020 [25]	**Inclusion criteria:** Healthy people were selected from people who came to our hospital for annual physical examinations, and all of the results, including serological tests, liver function, and computed tomography scan and other tests, were in the normal range.<br>**Exclusion criteria:** all participants had not received prior anticancer	diagnosed using Magnetic Resonance (MR), Computed Tomography (CT), HE staining of pathological sections, serum AFP levels, and chronic liver disease history	16S rRNA V4 (Illumina HiSeq 2500 platform)	-80°C	CTAB DNA extraction method

	treatment; no other diseases, such as heart disease or hypertension, were present; and they did not take drugs such as antibiotics, prebiotics, or other drugs in the last 6 months. In addition, healthy participants who had intestinal and liver-related diseases				
Sung et al.2019 [26]	**Exclusion criteria:** 1) they had received an antibiotics treatment before stool sample collection, 2) fecal sample collection would interfere with treatment, or 3) they were diagnosed with another disease that could influence conscious level (eg, a cerebral vascular accident or metabolic encephalopathy).	Cirrhosis was diagnosed on the basis of clinical and laboratory data, including ultrasonography and symptoms and signs of portal hypertension. The severity of HE was graded using the West Haven Criteria, which are based on the level of impairment to autonomy, changes in consciousness, intellectual function, behavior, and the dependence on therapy.	16S rRNA V3-V4 (Illumina MiSeq); Metagenomics (Illumina HiSeq)	-80°C	a modification of the PowerSoil DNA isolation kit (Cat. #12888-100; MoBio, Carlsbad, CA)
Astbury et al.2020 [27]	**Inclusion criteria:** NASH was diagnosed on the basis of the following criteria: appropriate exclusion of other causes of liver disease including alcohol, drugs, autoimmune or viral hepatitis, or cholestatic or metabolic/genetic liver disease; a weekly ethanol consumption of <140 g in women and <210 g in men and a liver biopsy showing steatohepatitis with or without cirrhosis. The Fatty Liver Inhibition of Progression (FLIP) Consortium algorithm was used as a diagnostic	liver biopsy	16S rRNA V4 (Illumina MiSeq)	-80°C	QIAmp DNA Stool Mini Kit (Qiagen; cat 51504)

	indicator where histological diagnosis of NASH requires presence of steatosis, ballooning, and lobular inflammation, and those without all three features are identified as having fatty liver while those lacking steatosis (<5% hepatocytes with fat accumulation) are not considered as having NASH				
Bajaj et al.2020 [28]	**Inclusion criteria:** healthy controls, who were free of chronic diseases and were not on any prescription medications.<br>**Exclusion criteria:** patients with problem drinking (AUDIT-10 score >8), illicit drug use, edentulous or with dentures or with active periodontitis, unable to provide samples and those who were hospitalized within the last 30 days.	Cirrhosis was diagnosed using liver biopsy, elastography, endoscopy suggestive of varices in chronic liver disease patients, evidence of frank decompensation or radiological evidence of cirrhosis.	16S rRNA V1-V2 (PGM Ion Torrent)	-80°C	standard published techniques
Chen et al.2020 [53]	**Inclusion criteria:** healthy individuals (a) alcohol free history or alcohol consumption less than 140 g per week in males, less than 70 g per week in females; (b) smooth and soft stool that was sausage or snake shaped, and (c) voluntary participation in this study. Patients: (a) alcohol free history or alcohol consumption less than 140 g per week in males and, less than 70 g per week in females; and (b) they meet the diagnostic criteria for chronic hepatitis B according to the "EASL 2017 Clinical Practice Guidelines on the management of hepatitis B virus infection."<br>**Exclusion criteria:** (a) symptoms of	computed tomography (CT)<br>magnetic resonance imaging (MRI)<br>ultrasonography	16S rRNA V3-V4 (HiSeq 2500 (Illumina, San Diego, CA, United States))	-80°C	QIAamp PowerFecal DNA Kit (Qiagen, DE)

digestive system disorders, such as hematochezia, constipation, abdominal distention, abdominal pain, diarrhea, and jaundice within 1 month; (b) abnormal results of several tests, including: routine blood, liver function, renal function, blood fat, fasting blood glucose, HBsAg, routine fecal and fecal occult blood tests; (c) an enteritis diagnosis within 1 month; (d) chronic obstructive pulmonary disease, renal insufficiency and other systemic diseases; (e) autoimmune disease; (f) chronic fatigue syndrome and neuropsychic disease; (g) a history of antibiotic, microecological preparation, gastrointestinal motility medicine, laxative, weight loss drug, glucose lowering, blood fat regulation, glucocorticoid, or immunosuppressant treatment within 1 month; (h) history of organic diseases in the digestive system, such as gastrointestinal polyposis, ulcers, cirrhosis, and malignancies; (i) history of gastrointestinal surgery; or (j) a family history of diabetes, hypertension, coronary heart disease, metabolic syndrome, etc. Patients: (a) a history of antibiotic, microecological preparation, gastrointestinal motility medicine, weight loss drug, glucose lowering, blood fat regulation, and glucocorticoid or immunosuppressant treatment within 1 month; (b) other causes of liver disease such as NAFLD, autoimmune liver disease, hepatitis A, hepatitis C, hepatitis D,

	hepatitis E and liver parasite infection; (c) chronic fatigue syndrome and neuropsychic disease; (d) a history of gastrointestinal surgery; or (e) a family history of diabetes, hypertension, coronary heart disease, metabolic syndrome, etc.				
Cox et al.2020 [29]	**Inclusion criteria:** The healthy controls were free of chronic diseases, were not on any prescription medications and had normal liver function.<br>**Exclusion criteria:** Patients with alcohol misuse and/or illicit drug usage (defined by DSM-V criteria), inability to provide samples and those who were hospitalized within the last 30 days.	The diagnosis of cirrhosis was based on liver biopsy if available, ultrasound-based elastography or clinical, biochemical and radiological evidence of cirrhosis.<br>Decompensation was defined as variceal bleeding in the immediate past, ascites under treatment, hepatic encephalopathy under treatment and those with jaundice.	16S rRNA	-80°C	NA
Lapidot et al.2020 [30]	**Inclusion criteria:** Patients with HCV cirrhosis were recruited before HCV eradication. Subjects in the control group had no history of liver disease and no significant alcohol consumption.<br>**Exclusion criteria:** 1. Treatment with antibiotics, probiotics, prebiotics, and laxatives during the previous 3 months.<br>2. Other cirrhosis etiologies, including hepatitis B virus (HBV), human immunodeficiency virus (HIV), alcoholic steatohepatitis, cholestatic	Cirrhosis diagnosis was based on histological and/or clinical findings (laboratory parameters, imaging findings, signs of portal hypertension at liver imaging or endoscopy)	16S rRNA V4 (Illumina MiSeq)	-80°C	PureLink Microbiome DNA purification kit (Invitrogen, Thermo Fisher Scientific, Carlsbad, CA, USA)

	disorders (primary biliary cholangitis or primary sclerosing cholangitis), and inherited liver disorders leading to cirrhosis, i.e., hemochromatosis, Wilson's disease, and alpha-1 antitrypsin deficiency.<br>3. A diagnosis of inflammatory bowel disease or celiac disease.<br>4. A diagnosis of other malignancy (not HCC) in the last 3 years.				
Oh et al.2020 [31]	**Inclusion criteria:** patients proven by either biopsy or by meeting imaging criteria. A detailed medical history, physical examination, and testing to rule out other causes of chronic liver diseases, and fasting laboratory tests.<br>**Exclusion criteria:** significant alcohol intake (>10 g/day in females or >20 g/day in males) for at least 3 consecutive months over the previous 12 months or if the quantity of alcohol consumed could not be reliably ascertained; clinical or biochemical evidence of liver diseases other than NAFLD (e.g., viral hepatitis, HIV, coeliac disease, cystic fibrosis, autoimmune hepatitis); metabolic and/or genetic liver disease (e.g., Wilson's disease, haemochromatosis, polycystic liver disease, alpha-1-antitrypsin deficiency, dysbetalipoproteinaemia); clinical or laboratory evidence of systemic infection or any other clinical evidence of liver disease associated with hepatic steatosis; use of drugs known to cause	Participants met the criteria for NAFLD-related cirrhosis if they had NAFLD according to the definition above and biopsy-proven cirrhosis (histologic stage 4 fibrosis). For the diagnosis of advanced fibrosis, MRE is the most accurate non-invasive test to date. The presence of cirrhosis was determined by a liver stiffness assessment by MRE with a threshold ≥ 4.69 kPa. If MRE was not performed due to contraindications, then a transient elastography assessment with a VCTE threshold ≥ 11.8 kPa was used as criteria for advanced fibrosis.	Metagenomic sequencing (using Illumina MiSeq platform)	-80°C	Qiagen MagAttract PowerSoil DNA kit

	hepatic steatosis (e.g., amiodarone, glucocorticoids, methotrexate, L-asparaginase and valproic acid) for at least 3 months in the last past 6 months; history of bariatric surgery; presence of systemic infectious illnesses; females who were pregnant or nursing at the time of the study; contraindications to MRI (e.g., metal implants, severe claustrophobia, body circumference greater than the imaging chamber); any other condition(s) which, based on the principal investigator's opinion, may significantly affect the participant's compliance, competence, or ability to complete the study.				
Sydor et al.2020 [32]	**Exclusion criteria:** Patients with a history of alcohol intake or those with viral hepatitis.	Cirrhosis was diagnosed by histology or clinical signs of portal hypertension.	16S rRNA MiSeq (2×250 bp, Illumina, Hayward, CA, USA)	-80°C	QIamp-DNA isolation kit (Qiagen, Hilden, Germany)
Yang et al.2020 [33]	**Inclusion criteria:** All the participants in this study had lived in the Pearl River Delta in Guangdong Province for more than 3 years.<br>**Exclusion criteria:** Patients who were infected by human immunodeficiency virus (HIV) or hepatitis C virus (HCV) or had other diseases, such as alcoholic hepatitis, fatty liver disease, acute or chronic infectious diseases, autoimmune diseases, or non-Hepatitis B liver diseases; Patients with a body mass index (BMI) (kg/m2) of less than 18.5 or more than 23.9; Patients who with any gastrointestinal disease,	Liver biopsy or imaging examinations including trans-abdominal ultrasound, computed X-ray tomography (CT), or nuclear magnetic resonance (NMR) were performed when necessary.	16S rRNA V4 (NovaSeq PE250 platform)	-80°C	E.Z.N.A. ®Stool DNA Kit (D4015, Omega, Inc., USA)

	which may be linked to a leaky gut and bacterial translocation to the liver. In addition, patients who took antibiotics or traditional Chinese medicine orally within the previous 3 months.				
Zeng et al.2020 [34]	**Inclusion criteria:** The cases and controls were matched for gender, body mass index (BMI), dietary habits and other demographic factors.<br>**Exclusion criteria:** patients infected with other viruses (such as HIV, HCV, HDV, HEV, etc), smoking or alcohol consumption, antibiotic or immunosuppressive drugs treatment within 6 months, acute or chronic infectious diseases, other liver diseases (such as nonalcoholic fatty liver disease, steatohepatitis, alcoholic liver disease, cholestatic liver disease and HCV-induced liver disease, etc), internal disease (such as IBS, IBD, etc), metabolic diseases (such as diabetes mellitus, obesity, fatty liver disease, etc), other malignancy (such as gastric cancer, pancreatic cancer, oesophageal cancer, etc) and autoimmune disease (such as rheumatoid arthritis, multiple sclerosis, etc).	the diagnosis of LC was made according to the 'Guideline of Prevention and Treatment for Chronic Hepatitis B (2015 Version)', enacted by the Chinese Society of Hepatology and Chinese Society of Infectious Diseases, Chinese Medical Association.	16S rRNA V3-V4 (HiSeq 2500, Illumina)	-80°C	QIAamp Fast DNA stool mini kit (Qiagen)
Huan et al.2021 [35]	**Inclusion criteria:** patients with an identified risk factor for cirrhosis; portal hypertension and liver dysfunction; evidence of LC on abdominal CT or color doppler ultrasound imaging	abdominal CT or color doppler ultrasound imaging	16S rRNA V3-V4 (Illumina MiSeq PE300 system (OE Biotech Co., Ltd, Shanghai, China))	-80°C	DNeasy PowerSoil Kit (Tiangen Biotechnology Company, Beijing, China)

Ponziani et al.2021 [36]	**Inclusion criteria:** age ≥ 18 years, absence of systemic or intestinal pathologies associated with gut microbiota alterations (eg celiac disease, inflammatory bowel diseases, diabetes mellitus etc) and complete abstinence from alcohol consumption for at least one year. A group of subjects without cirrhosis comparable for age and sex distribution and meeting the same eligibility criteria were enrolled as controls. The only comorbidities allowed for the control group were hypertension, prior stroke (more than 1 year before the enrolment) or non-obstructive peripheral vascular disease and mild chronic obstructive pulmonary disease.<br>**Exclusion criteria:** Patients with previous or active tumours, chronic neurodegenerative or muscle diseases, use of probiotics, prebiotics or antibiotics during the previous 3 months, and those on vegetarian or vegan diet; Actively exercising subjects.	NA	16S rRNA V3-V4 (Illumina MiSeq)	-80°C	QIAmp Fast DNA Stool mini kit (Qiagen, Germany)
Ren et al.2021 [37]	**Inclusion criteria:** Controls: Medical history and physical examination results indicated that the patients were in good health, with no history of liver or GI diseases, and no chronic diseases such as hypertension or diabetes. Liver cirrhosis diagnosed based on imaging data, biochemical indicators, clinical data, and pathologic examination; 18-60y of age; Complete abdominal	Liver cirrhosis diagnosed based on imaging data, biochemical indicators, clinical data, and pathologic examination	Metagenomic sequencing (using Illumina HiSeq platform)	-80°C	QIAamp PowerFecal Pro DNA Kit

	computed tomography (CT) examination; No use of antibiotics or microbial preparations within 4 wk of fecal collection; and No history of gastrointestinal (GI) bleeding within 4 wk of fecal collection.<br>**Exclusion criteria:**<br>Liver cancer combined with other malignant tumors;<br>Severe respiratory and circulatory diseases; and<br>History of GI surgery.				
Alvares-da-Silva et al.2022 [38]	**Inclusion criteria:** Controls were subjects without chronic diseases, any illicit drug use, alcohol abuse (AUDIT-10≥8), or prescription medications.<br>**Exclusion criteria:**<br>Patients with an unclear history of cirrhosis, any illicit drug use, alcohol abuse (AUDIT-10≥8), and those unable to consent, provide dietary history or samples	Cirrhosis was diagnosed by liver biopsy, transient elastography, presence of signs of portal hypertension, varices, or thrombocytopenia in patients with chronic liver disease, or frank decompensation. Decompensation was defined as ascites, hepatic encephalopathy (HE), variceal bleeding or jaundice.	16S rRNA V1-V2 (PGM Ion Torrent)	-80°C	NA
Baltazar-Díaz et al.2022 [39]	**Inclusion criteria:**<br>(a) Decompensated inpatients with diagnosis of cirrhosis by biopsy, or by clinical criteria with imaging confirmation, within Child–Pugh category B or C. (b) Age between 18 to 70 years, (c) BMI between 18.5 and 29.9 kg/m^2. Healthy subjects (control group): (a) Age between 18 to 70 years, (b) BMI between 18.5 and 29.9	by biopsy, or by clinical criteria with imaging confirmation, within Child–Pugh category B or C. Decompensated alcoholic cirrhosis was defined when patients had 1 or more of the following complications: ascites, jaundice,	16S rRNA V3-V4 Illumina MiSeq System (Illumina, San Diego, CA, USA)	-80°C	QIAamp PowerFecal DNA Kit (QIAGEN, Hilden, Germany)

	kg/m2, (c) No current or past SARS-CoV-2 infection for at least 3 months before recruiting, (d) No use of prebiotics/probiotics 4 weeks before recruiting, (e) No use of antibiotics 3 months before recruiting, (f) No known allergies or intolerances to fiber sources, (g) Non-vegetarian or smokers, (h) Alcohol intake 28 g alcohol/week.<br>**Exclusion criteria:**<br>Excessive consumption of alcohol 72 h before recruiting (>48 g alcohol/day), use of prebiotics/probiotics 4 weeks before recruiting, HIV, hepatitis B or C infection, current or past severe SARS-CoV-2 infection, or any chronic gastrointestinal or autoimmune diseases	gastrointestinal bleeding, hepatic encephalopathy and/or bacterial infections. All inpatients were under lactulose or antibiotic prophylaxis.			
Hua et al.2022 [40]	**Inclusion criteria:** The diagnosis was based on the guidelines for chronic hepatitis B diagnosis of the American Association for the Study of Liver Diseases. All patients tested positive for serum HBsAg for >24 weeks.<br>**Exclusion criteria:** a positive pregnancy test in females, received immunomodulator treatment in the previous 6 months, coinfection with human immunodeficiency virus, presence of thyroid dysfunction, alcoholic hepatitis, autoimmune diseases, and psychological issues.	NA	16S rRNA V4	-80°C	NA
Maslennikov et al.2022 [41]	**Inclusion criteria:** diagnosis of cirrhosis verified by histological examination or clinical, biochemical,	by histological examination or clinical, biochemical, and	16S rRNA V3-V4 Illumina MiSeq System	-80°C	AmpliPrime DNA-sorb-AM kit (NextBio,

	and ultrasound findings, and age between 18 and 70 years.<br>**Exclusion criteria:** use of lactulose, lactitol, or other prebiotics, probiotics, antibiotics, or metformin in the past 6 wk, alcohol consumption in the past 6 wk, or diagnosis of inflammatory bowel disease, cancer, or any other serious disease.	ultrasound findings	(Illumina, San Diego, CA, USA)		Moscow, Russia)
Shu et al.2022 [42]	**Exclusion criteria:** (1) Patients not treated with antibiotics; (2) co-infection with hepatitis C virus, hepatitis D virus, human immunodeficiency virus or decompensated liver disease, pregnancy and alcoholism within 1 year before the treatment; (3) history of therapy with systemic corticosteroids, antineoplastic or immunomodulator drugs.	the diagnosis of decompensated cirrhosis and compensated cirrhosis was made according to the 'Guidelines for diagnosis and Treatment of cirrhosis (2020 Version)'.	16S rRNA V3-V4 Illumina HiSeq System	-80°C	QIAamp Fast DNA stool mini kit (Qiagen)
Sun et al.2022 [43]	**Inclusion criteria:**<br>(i) They were over 18 years old<br>(ii) Their HBsAg positive and/or HBV-DNA positive were more than 6 months<br>(iii) Their imaging examination suggested cirrhosis, portal hypertension, esophageal varices, splenomegaly, with or without ascites, without infection, hepatic encephalopathy, and gastrointestinal bleeding<br>patients whose results of physical examination, blood routine, urine routine, liver function, kidney function, serological markers of HBV, and abdominal ultrasound were within	Their imaging examination suggested cirrhosis, portal hypertension, esophageal varices, splenomegaly, with or without ascites, without infection, hepatic encephalopathy, and gastrointestinal bleeding	16S rRNA V3-V4 Illumina NovaSeq System	-80°C	the cetyltrimethylammonium bromide method

	the normal range, and those without heart, brain, kidney, and lung diseases, were selected as healthy controls (HC).<br>**Exclusion criteria:** patients complicated by liver diseases such as alcoholic liver disease, autoimmune liver disease, fatty liver disease, and other viral liver diseases; patients who had systematically used antibiotics, probiotics, and proton pump inhibitors within 1 month before enrollment; and pregnant and lactating women.				
Ullah et al.2022 [44]	**Inclusion criteria:** All control group members had normal liver biochemistry tests without any evidence of hepatic or other diseases. (a) alcohol-free history or alcohol consumption less than 140 g per weekin males, less than 70 g per week in females; (b) smooth and soft stool that was sausage or snake-shaped, and (c) voluntary participation in this study.<br>**Exclusion criteria:** (a) symptoms of digestive system disorders, such as hematochezia, constipation, abdominal distention, abdominal pain, diarrhea, and jaundice within 1 month; (b) abnormal results of several tests, including: routine blood, liver function, renal function, blood fat, fasting blood glucose, HBsAg, routine faecal and faecal occult blood tests; (c) an enteritis diagnosis within 1 month; (d) chronic obstructive pulmonary disease, renal insufficiency and other	liver cirrhosis was confirmed from the medical history of the patients.	16S rRNA V3-V4 Illumina MiSeq 250 System	-80°C	Stool Mini QIA-amp DNA extraction kit (Qiagen Valencia, CA)

	systemic diseases; I autoimmune disease; (f) chronic fatigue syndrome and neuropsychic disease; (g) a history of antibiotic, microecological preparation, gastrointestinal motility medicine, laxative, weight loss drug, glucose lowering, blood fat regulation, glucocorticoid, or immunosuppressant treatment within 1 month; (h) history of organic diseases in the digestive system, such as gastrointestinal polyposis, ulcers, cirrhosis, and malignancies; (i) history of gastrointestinal surgery; or (j) a family history of diabetes, hypertension, coronary heart disease, metabolic syndrome, etc.				
Zhou et al.2022 [45]	**Inclusion criteria:** the presence of decompensated liver cirrhosis and ascites fluid.<br>**Exclusion criteria:** upper gastrointestinal bleeding, intake of antibiotic therapy in the previous 2 weeks, hepatocellular carcinoma, other associated causes of ascites (such as tubercular or malignant ascites), and severe cardiopulmonary or renal complications.	The diagnosis of cirrhosis was based on clinical, biochemical, radiological (ultrasonography), and endoscopic findings (presence of varices) or liver histology.	16S rRNA V3-V4 Illumina NovaSeq platform	-80°C	the CTAB/SDS method
Chen et al.2023 [46]	**Inclusion criteria:** No participants suffered hypertension, diabetes, inflammatory bowel disease, or necrotizing enteritis. Besides, none of these participants took proton pump inhibitors, antibiotics, or probiotics within 2 weeks before sample collection.	Patients were diagnosed with LC in terms of medical history, liver biopsy, clinical symptoms, laboratory tests, imaging tests, histological examinations, and complications. The	16S rRNA V3-V4 Illumina HiSeq 2500 platform (Illumina, San Diego, CA, USA)	-80°C	the QIAamp DNA Mini Kit (Qiagen, Hilden, Germany)

		diagnosis for CLC was based on the criteria described in Chinese guidelines on the management of liver cirrhosis (abbreviated version)			
Lai et al.2023 [47]	**Inclusion criteria:** For all participants, no exposure to antibiotics or probiotics was allowed for at least three months before stool collection.	Diagnosis of LC was based on clinical portal hypertension and supporting images, including ultrasonography (Fibroscan), abdominal computed tomography (CT), and magnetic resonance imaging (MRI), or definitive liver pathology.	16S rRNA V3-V4 Illumina MiSeq platform (Illumina, San Diego, CA, USA)	-80°C	the QIAamp DNA Stool Mini Kit (Qiagen, Hilden, Germany)
Wang et al.2023 [48]	**Inclusion criteria:** Healthy volunteers: medical history, physical examination, chest X-ray, routine blood tests, blood glucose, liver function, kidney function, and other physical and chemical examinations, which revealed no diseases of the brain, heart, lung, liver, kidney, or other major organ systems.<br>**Exclusion criteria:** Patients with diabetes, mental illness, hepatocellular carcinoma, primary sclerosing cholangitis, autoimmune hepatitis, gastrointestinal diseases, and a history of gastrointestinal surgery; patients who underwent hormone replacement therapy or used antipsychotic	A liver biopsy, the discovery of varices or portal hypertension, or the decompensation of chronic liver disease were all used to diagnose liver cirrhosis.	16S rRNA V4 Illumina NovaSeq platform (Illumina, San Diego, CA, USA)	-80°C	CTAB/SDS technique

	medication, antibiotics, or probiotics within the 6 weeks prior to the study, as well as patients with active alcohol use disorder (AUDIT-10 > 8).				
Wu et al.2023 [49]	**Inclusion criteria:** patients with liver cirrhosis were age ≥ 18 years, and the diagnosis of cirrhosis was confirmed by liver biopsy or a combination of clinical, biochemical, ultrasound, elastographic and endoscopic examinations.<br>**Exclusion criteria:** use of gut microecological agents such as probiotics and prebiotics, antibiotics and ursodeoxycholic acid in last 3 months, previous intestinal resection and carcinoma, intestinal infectious, immune diseases, diabetes mellitus, obesity, psychosomatic disorders, organ failures, pregnant/lactating women and lack of informed consent.	the diagnosis of cirrhosis was confirmed by liver biopsy or a combination of clinical, biochemical, ultrasound, elastographic and endoscopic examinations.	16S rRNA V3-V4 DNBSEQ platform (Beijing Genomics Institution)	-80°C	MagPure Stool DNA KF Kit B
Yan et al.2023 [50]	**Inclusion criteria:** (i) patients diagnosed with primary liver cancer; (ii) aged 18-75 years; (iii) patients with BCLC C and D stage; (iv) patients had a history of chronic hepatitis B (HBsAg) positive > 6 months; (v) patients were not treated with drugs to regulate gut microbes during the past 1 month. (i) Health controls with theWorld Health Organization's definition of health (i.e., in good physical, mental, and social conditions); (ii) Health controls have no abnormalities in liver function testing; (iii) Health controls have no history of alcohol consumption.	NA	16S rRNA V3-V5, 18S rRNA, ITS	-80°C	MagPure Soil DNA KF Kit (Magen, Germany)

	**Exclusion criteria:** (i) patients with cholangiocarcinoma; (ii) patients with metastatic liver cancer; (iii) patients comorbid with other types of tumor; (iv) patients who were lost to follow-up; (v) patients with incomplete clinical data.				
Zhang et al.2023 [51]	**Inclusion criteria:** Patients who were diagnosed as chronic HBV liver disease with positive HBV surface antigen test for at least 6 months were recruited.<br>**Exclusion criteria:** liver diseases caused by other viruses and alcohol, and the interference of other cancers and chronic diseases.	NA	16S rRNA V3-V4 Illumina	-80°C	NA
Efremova et al.2024 [52]	**Inclusion criteria:** the diagnosis of which was made based on histology or a combination of physical examination, laboratory and instrumental data, signed written informed consent, and an age of between 18 and 70 years.<br>**Exclusion criteria:** use of drugs that could affect the composition of the gut microbiota (lactulose, lactitol, or other prebiotics, probiotics, antibiotics, and metformin) in the preceding six weeks; alcohol consumption in the preceding six weeks; current infection (except spontaneous bacterial peritonitis); inflammatory bowel disease, cancer, renal failure, or any other serious disease.	the diagnosis of which was made based on histology or a combination of physical examination, laboratory and instrumental data	16S rRNA	-80°C	NA

Supplementary Table S6. Methodology and findings of the included studies assessing beta diversity for the patient vs. control group comparison.

Study	Metric	Analysis	Finding
Chen et al.2011 [1]	UniFrac (unweighted)	PCA<br>PLS-DA	LC/ controls: sig. different
Wei et al.2013 [4]	-	PCA	LC/ controls: sig. different
Zhang et al.2013 [3]	UniFrac (G-full-tree)	PCoA	LC/ controls: sig. different
Bajaj et al.2014 [5]	-	PCA	LC / controls: sig. different
Ahluwalia et al.2016 [8]	UniFrac	PCoA	LC / controls: sig. different
Bajaj et al.2016 [9]	UniFrac (weighted)	PCoA	LC/ controls: sig. different
Chen et al.2016 [10]	UniFrac (weighted)	PCoA<br>PLS-DA	LC/ controls: sig. different
Wei et al.2016 [11]	UniFrac (unweighted)	PCA	LC/ controls: sig. different
Lee et al.2023 [12]	UniFrac (unweighted)	PCoA	LC/ controls: sig. different
Heidrich et al.2018 [13]	Bray-Curtis	PCoA	LC/ controls: sig. different
Bajaj et al.2018 [14]	UniFrac (weighted)	PCoA	LC/ controls: sig. different
Inoue et al.2018 [17]	UniFrac (weighted)<br>Bray-Curtis	PCoA	LC/ controls: sig. different
Liu et al.2018 [18]	Bray-Curtis	PCoA	LC/ controls: sig. different
Ponziani et al.2018 [19]	UniFrac (weighted)	PCoA	LC/ controls: sig. different
Shao et al.2018 [20]	Bray-Curtis	PCoA	LC/ controls: sig. different
Sun et al.2018 [21]	UniFrac (weighted and unweighted)	PCoA	LC/ controls: sig. different
Caussy et al.2019 [22]	UniFrac (unweighted)	PCoA	LC/ controls: no sig. different
Deng et al.2019 [23]	Bray-Curtis	PCoA	LC/ controls: no sig. different
Jin et al.2019 [24]	UniFrac (weighted and unweighted)<br>Bray-Curtis	PCoA	LC/ controls: sig. different
Zheng et al.2020 [25]	UniFrac (unweighted)	PCoA<br>NMDS	LC/ controls: sig. different
Sung et al.2019 [26]	Bray-Curtis	PCoA<br>NMDS	LC/ controls: no sig. different
Astbury et al.2020 [27]	UniFrac (unweighted)	PCoA	LC/ controls: sig. different
Chen et al.2020 [53]	Bray-Curtis	NMDS	LC/ controls: sig. different
Lapidot et al.2020 [30]	UniFrac (weighted and unweighted)	PCoA	LC/ controls: sig. different
Oh et al.2020 [31]	UniFrac (weighted)	PCoA	LC/ controls: sig. different
Yang et al.2020 [33]	UniFrac (weighted)	PCoA	LC/ controls: sig. different
Zeng et al.2020 [34]	UniFrac (weighted)	PCoA	LC/ controls: sig. different
Huan et al.2021 [35]	UniFrac (weighted)	PCoA	LC/ controls: no sig. different

Ponziani et al.2021 [36]	UniFrac (weighted)	PCoA	LC/ controls: sig. different
Alvares-da-Silva et al.2022 [38]	Bray-Curtis	PCoA	LC/ controls: sig. different
Baltazar-Díaz et al.2022 [39]	UniFrac (weighted and unweighted)	PCoA	LC/ controls: sig. different
Shu et al.2022 [42]	UniFrac (weighted)	PCoA	LC/ controls: sig. different
Sun et al.2022 [43]	Jaccard	PCoA	LC/ controls: sig. different
Ullah et al.2022 [44]	Bray-Curtis	NMDS	LC/ controls: sig. different
Zhou et al.2022 [45]	UniFrac (unweighted)	PCoA<br>NMDS	LC/ controls: sig. different
Chen et al.2023 [46]	UniFrac (unweighted)	PCoA<br>NMDS<br>PCA	LC/ controls: sig. different
Lai et al.2023 [47]	UniFrac (unweighted)<br>Bray-Curtis	PCoA	LC/ controls: no sig. different
Wang et al.2023 [48]	UniFrac (weighted and unweighted)<br>Bray-Curtis	PCoA<br>PCA<br>NMDS	LC/ controls: sig. different
Wu et al.2023 [49]	UniFrac (weighted)	PCoA	LC/ controls: sig. different
Yan et al.2023 [50]	UniFrac (weighted)	PCoA	LC/ controls: sig. different
Zhang et al.2023 [51]	Bray-Curtis	PCoA	LC/ controls: sig. different

Abbreviation: sig. = significantly

Supplementary Figure S7. Forest plots for the association between Faith's PD and LC.

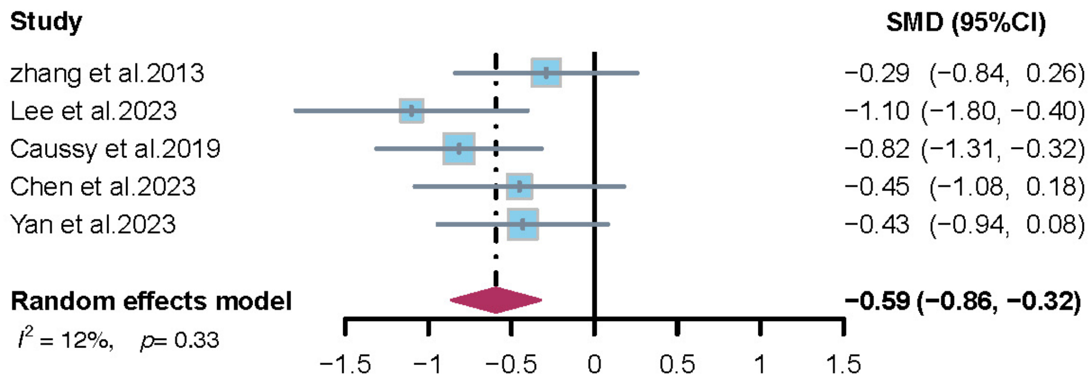

Supplementary Table S8. Publication bias tests (Egger's test) for the association between alpha diversity and LC.

Alpha diversity	Coefficient	Standard error	*P*	t	95%CI of intercept
Shannon	-2.406	1.072	0.019	-2.245	-0.367, 0.722
Chao1	-0.288	2.363	0.775	-0.122	-1.939, 1.103
Simpson	1.090	1.720	0.290	0.634	-1.787, 0.297
Observed species	-2.523	2.743	0.022	-0.920	-0.653, 3.273
PD	-0.637	4.183	0.569	-0.152	-3.650, 3.985
ACE	-0.589	4.092	0.568	-0.144	-2.425, 3.153

Supplementary Table S9. Summary of findings for each taxon reported at phylum level.

**Phylum**	N	Increased (%)	Decreased (%)	No difference (%)	Results
Bacteroidetes	22	4 (18.2)	11 (50.0)	7 (31.8)	Not consistent
Proteobacteria	22	19 (86.4)	0 (0)	3 (13.6)	Not consistent
Firmicutes	19	4 (21.1)	8 (42.1)	7 (36.8)	Not consistent
Actinobacteria	9	3 (33.3)	2 (22.2)	4 (44.4)	Not consistent
Fusobacteria	5	3 (60.0)	0 (0)	2 (40.0)	Not consistent
Verrucomicrobia	5	2 (40.0)	0 (0)	3 (60.0)	Not consistent
Tenericutes	3	0 (0)	1 (33.3)	2 (66.7)	Not consistent
Cyanobacteria	2	0 (0)	0 (0)	2 (100)	Need verification
Euryarchaeota	2	0 (0)	1 (50.0)	1 (50.0)	Not consistent
TM7	2	0 (0)	0 (0)	2 (100)	Need verification

Supplementary Table S10. Summary of findings for each taxon reported at class level.

**Class**	N	Increased (%)	Decreased (%)	Results
Gammaproteobacteria	7	7 (100)	0 (0)	Increased
Bacilli	16	16 (100)	0 (0)	Increased
Clostridia	13	0 (0)	13 (100)	Decreased
Negativicutes	3	3 (100)	0 (0)	Increased
Bacteroidia	7	4 (57.1)	3 (42.9)	Not consistent
Erysipelotrichi	3	1 (33.3)	2 (66.7)	Not consistent
Actinobacteria	2	1 (50.0)	1 (50.0)	Need verification
Coriobacteria	2	1 (50.0)	1 (50.0)	Need verification

Supplementary Table S11. Summary of findings for each taxon reported at family level.

**Family**	N	Increased (%)	Decreased (%)	No difference (%)	Results
Enterobacteriaceae	19	17 (89.5)	0 (0)	2 (10.5)	Increased
Streptococcaceae	19	13 (68.4)	0 (0)	6 (31.6)	Not consistent
Lachnospiraceae	17	2 (11.8)	14 (82.4)	1 (5.9)	Not consistent
Bacteroidaceae	16	3 (18.8)	8 (50.0)	5 (31.3)	Not consistent
Prevotellaceae	16	1 (6.3)	5 (31.3)	10 (62.5)	Not consistent
Ruminococcaceae	16	1 (6.3)	11 (68.8)	4 (25.0)	Not consistent
Veillonellaceae	15	6 (40.0)	2 (13.3)	7 (46.7)	Not consistent
Lactobacillaceae	10	7 (70.0)	0 (0)	3 (30.0)	Not consistent
Porphyromonadaceae	10	0 (0)	6 (60.0)	4 (40.0)	Not consistent
Rikenellaceae	9	1 (11.1)	4 (44.4)	4 (44.4)	Not consistent
Clostridiaceae	6	1 (16.7)	2 (33.3)	3 (50.0)	Not consistent
Bifidobacteriaceae	5	3 (60.0)	0 (0)	2 (40.0)	Not consistent
Enterococcaceae	5	3 (60.0)	0 (0)	2 (40.0)	Not consistent
Erysipelotrichaceae	5	2 (40.0)	1 (20.0)	2 (40.0)	Not consistent
Pasteurellaceae	5	4 (80.0)	0 (0)	1 (20.0)	Not consistent
Clostridiales XIV	4	0 (0)	4 (100)	0 (0)	Decreased
Coriobacteriaceae	4	1 (25.0)	1 (25.0)	2 (50.0)	Not consistent
Peptostreptococcaceae	4	2 (50.0)	1 (25.0)	1 (25.0)	Not consistent
Verrucomicrobiaceae	4	2 (50.0)	1 (25.0)	1 (25.0)	Not consistent
Barnesiellaceae	3	0 (0)	1 (33.3)	2 (66.7)	Not consistent
Fusobacteriaceae	3	3 (100)	0 (0)	0 (0)	Increased
Micrococcaceae	3	2 (66.7)	0 (0)	1 (33.3)	Not consistent
Mogibacteriaceae	3	1 (33.3)	0 (0)	2 (66.7)	Not consistent
Staphylococcaceae	3	2 (66.7)	1 (33.3)	0 (0)	Not consistent
Turicibacteraceae	3	0 (0)	1 (33.3)	2 (66.7)	Not consistent
Acidaminococcaceae	2	0 (0)	2 (100)	0 (0)	Need verification
Akkermansiaceae	2	2 (100)	0 (0)	0 (0)	Need verification
Carnobacteriaceae	2	0 (0)	0 (0)	2 (100)	Need verification
Christensenellaceae	2	0 (0)	1 (50.0)	1 (50.0)	Not consistent
Clostridiaceae_1	2	2 (100)	0 (0)	0 (0)	Need verification
Dehalobacteriaceae	2	0 (0)	0 (0)	2 (100)	Need verification
Desulfovibrionaceae	2	0 (0)	2 (100)	0 (0)	Need verification
Methanobacteriaceae	2	0 (0)	2 (100)	0 (0)	Need verification
Oscillospiraceae	2	1 (50.0)	1 (50.0)	0 (0)	Not consistent
Sutterellaceae	2	1 (50.0)	1 (50.0)	0 (0)	Not consistent
Tannerellaceae	2	1 (50.0)	1 (50.0)	0 (0)	Not consistent

Supplementary Table S12. Summary of findings for each taxon reported at genus level.

**Genus**	N	Increased (%)	Decreased (%)	No difference (%)	Results
Veillonella	15	13 (86.7)	0 (0)	2 (13.3)	Increased
Streptococcus	13	12 (92.3)	0 (0)	1 (7.7)	Increased
Bacteroides	12	2 (16.7)	7 (58.3)	3 (25.0)	Not consistent
Lactobacillus	12	8 (66.7)	0 (0)	4 (33.3)	Not consistent
Coprococcus	10	0 (0)	6 (60.0)	4 (40.0)	Not consistent
Faecalibacterium	10	0 (0)	6 (60.0)	4 (40.0)	Not consistent
Haemophilus	10	8 (80.0)	1 (10.0)	1 (10.0)	Increased
Megasphaera	10	7 (70.0)	2 (20.0)	1 (10.0)	Not consistent
Blautia	9	2 (22.2)	5 (55.6)	2 (22.2)	Not consistent
Dorea	9	1 (11.1)	5 (55.6)	3 (33.3)	Not consistent
Ruminococcus	8	0 (0)	5 (62.5)	3 (37.5)	Not consistent
Lachnospira	7	1 (14.3)	4 (57.1)	2 (28.6)	Not consistent
Paraprevotella	7	0 (0)	5 (71.4)	2 (28.6)	Not consistent
Bifidobacterium	6	1 (16.7)	2 (33.3)	3 (50.0)	Not consistent
Collinsella	6	2 (33.3)	2 (33.3)	2 (33.3)	Not consistent
Dialister	6	2 (33.3)	2 (33.3)	2 (33.3)	Not consistent
Eubacterium	6	1 (16.7)	4 (66.7)	1 (16.7)	Not consistent
Parabacteroides	6	0 (0)	4 (66.7)	2 (33.3)	Not consistent
Prevotella	6	2 (33.3)	2 (33.3)	2 (33.3)	Not consistent
Akkermansia	5	3 (60.0)	1 (20.0)	1 (20.0)	Not consistent
Anaerostipes	5	1 (20.0)	2 (40.0)	2 (40.0)	Not consistent
Bilophila	5	0 (0)	5 (100)	0 (0)	Decreased
Clostridium	5	0 (0)	3 (60.0)	2 (40.0)	Not consistent
Enterococcus	5	3 (60.0)	1 (20.0)	1 (20.0)	Not consistent
Escherichia_Shigella	5	3 (60.0)	0 (0)	2 (40.0)	Not consistent
Fusicatenibacter	5	2 (40.0)	2 (40.0)	1 (20.0)	Not consistent
Lachnoclostridium	5	2 (40.0)	2 (40.0)	1 (20.0)	Not consistent
Roseburia	5	0 (0)	1 (20.0)	4 (80.0)	Not consistent
Subdoligranulum	5	0 (0)	4 (80.0)	1 (20.0)	Decreased
Turicibacter	5	2 (40.0)	1 (20.0)	2 (40.0)	Not consistent

Alistipes	4	0 (0)	4 (100)	0 (0)	Decreased
Holdemania	4	1 (25.0)	3 (75.0)	0 (0)	Not consistent
Odoribacter	4	1 (25.0)	3 (75.0)	0 (0)	Not consistent
Phascolarctobacterium	4	0 (0)	3 (75.0)	1 (25.0)	Not consistent
Rothia	4	2 (50.0)	0 (0)	2 (50.0)	Not consistent
Adlercreutzia	3	0 (0)	1 (33.3)	2 (66.7)	Not consistent
Anaerotruncus	3	0 (0)	2 (66.7)	1 (33.3)	Not consistent
Atopobium	3	2 (66.7)	0 (0)	1 (33.3)	Not consistent
Catenibacterium	3	0 (0)	1 (33.3)	2 (66.7)	Not consistent
Coprobacillus	3	1 (33.3)	2 (66.7)	0 (0)	Not consistent
Eggerthella	3	1 (33.3)	0 (0)	2 (66.7)	Not consistent
Granulicatella	3	1 (33.3)	0 (0)	2 (66.7)	Not consistent
Oscillospira	3	0 (0)	0 (0)	3 (100)	No difference
Parasutterella	3	1 (33.3)	2 (66.7)	0 (0)	Not consistent
Prevotella_9	3	0 (0)	2 (66.7)	1 (33.3)	Not consistent
Ruminococcaceae_UCG-014	3	2 (66.7)	1 (33.3)	0 (0)	Not consistent
Terrisporobacter	3	2 (66.7)	1 (33.3)	0 (0)	Not consistent
[Eubacterium]_hallii_group	2	2 (100)	0 (0)	0 (0)	Need verification
[Eubacterium]_ventriosum_group	2	0 (0)	2 (100)	0 (0)	Need verification
Acidaminococcus	2	2 (100)	0 (0)	0 (0)	Need verification
Alloprevotella	2	2 (100)	0 (0)	0 (0)	Need verification
Bacillus	2	0 (0)	2 (100)	0 (0)	Need verification
Barnesiella	2	0 (0)	2 (100)	0 (0)	Need verification
Bifidobacteria	2	0 (0)	1 (50.0)	1 (50.0)	Not consistent
Bifidobacterium	2	0 (0)	1 (50.0)	1 (50.0)	Not consistent
Butyricicoccus	2	0 (0)	2 (100)	0 (0)	Need verification
Butyricimonas	2	0 (0)	2 (100)	0 (0)	Need verification
Christensenella	2	0 (0)	0 (0)	2 (100)	Need verification
Clostridium IV	2	0 (0)	2 (100)	0 (0)	Need verification
Clostridium_sensu_stricto_1	2	2 (100)	0 (0)	0 (0)	Need verification
Coprococcus_2	2	2 (100)	0 (0)	0 (0)	Need verification

Dehalobacterium	2	0 (0)	0 (0)	2 (100)	Need verification
Deltaproteobacteria	2	0 (0)	2 (100)	0 (0)	Need verification
Flavonifractor	2	1 (50.0)	0 (0)	1 (50.0)	Not consistent
Intestinibacter	2	2 (100)	0 (0)	0 (0)	Need verification
Klebsiella	2	2 (100)	0 (0)	0 (0)	Need verification
Lachnobacterium	2	0 (0)	0 (0)	2 (100)	Need verification
Marvinbryantia	2	0 (0)	2 (100)	0 (0)	Need verification
Megamonas	2	0 (0)	1 (50.0)	1 (50.0)	Not consistent
Methanobrevibacter	2	0 (0)	2 (100)	0 (0)	Need verification
Mitsuokella	2	0 (0)	2 (100)	0 (0)	Need verification
norank_f__Lachnospiraceae	2	0 (0)	2 (100)	0 (0)	Need verification
Oxalobacter	2	0 (0)	1 (50.0)	1 (50.0)	Not consistent
Pseudoflavonifractor	2	0 (0)	2 (100)	0 (0)	Need verification
Selenomonas	2	1 (50.0)	1 (50.0)	0 (0)	Not consistent
Slackia	2	0 (0)	0 (0)	2 (100)	Need verification
Staphylococcus	2	1 (50.0)	1 (50.0)	0 (0)	Not consistent
Tannerella	2	0 (0)	2 (100)	0 (0)	Need verification

Supplementary Figure S13. Funnel plots for assessing publication bias.

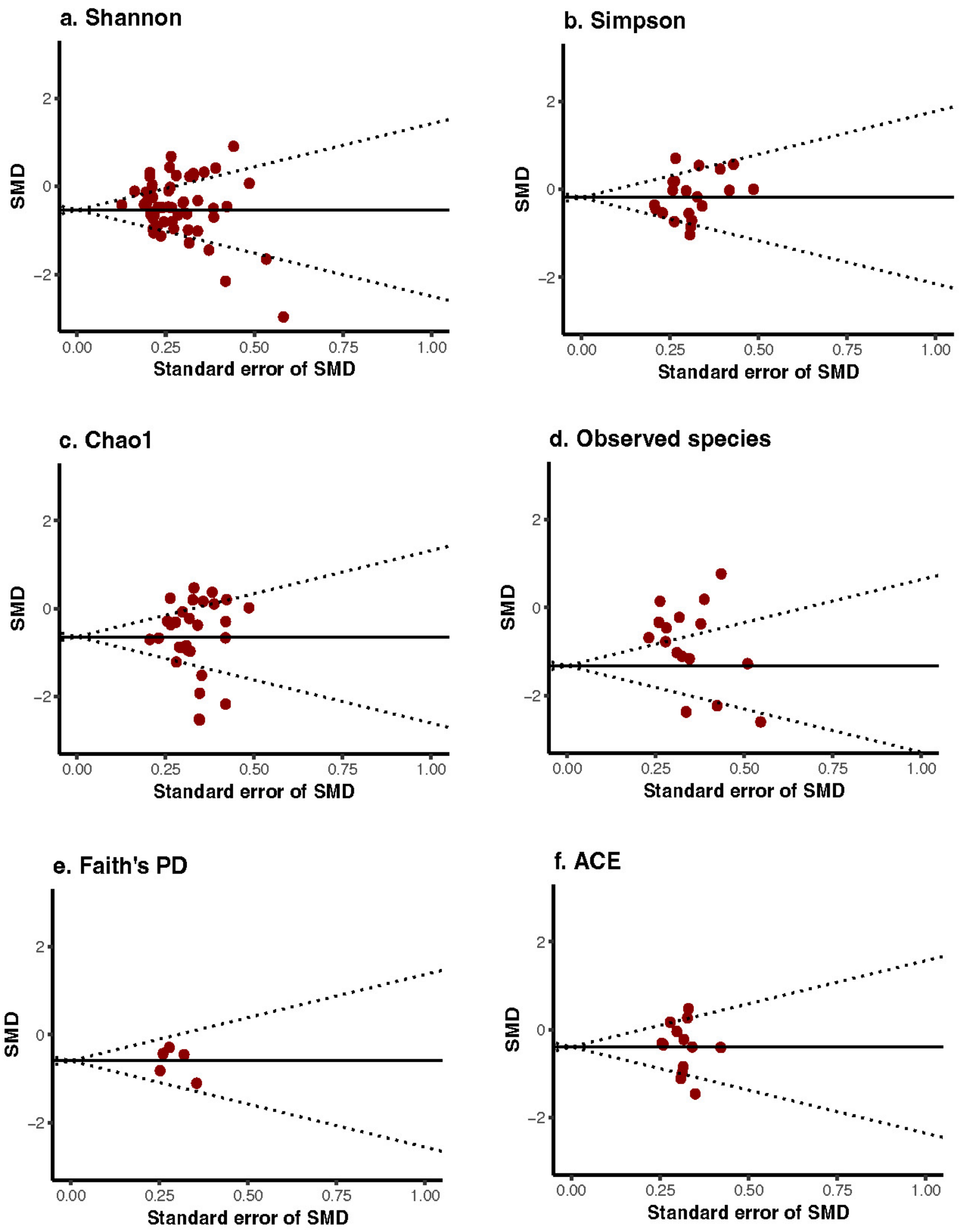

Supplementary Figure S14. Changes of relative abundance of 10 phyla included in analysis.

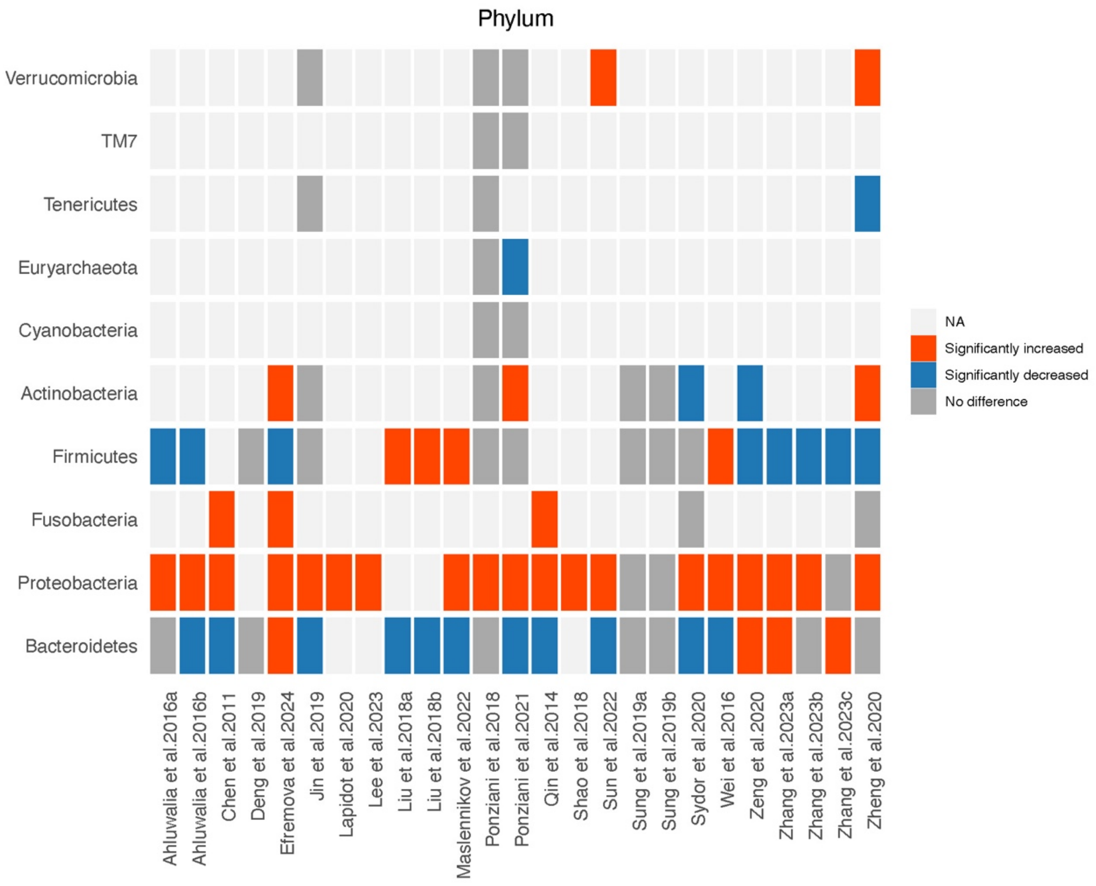

Supplementary Figure S15. Changes of relative abundance of 8 classes included in analysis.

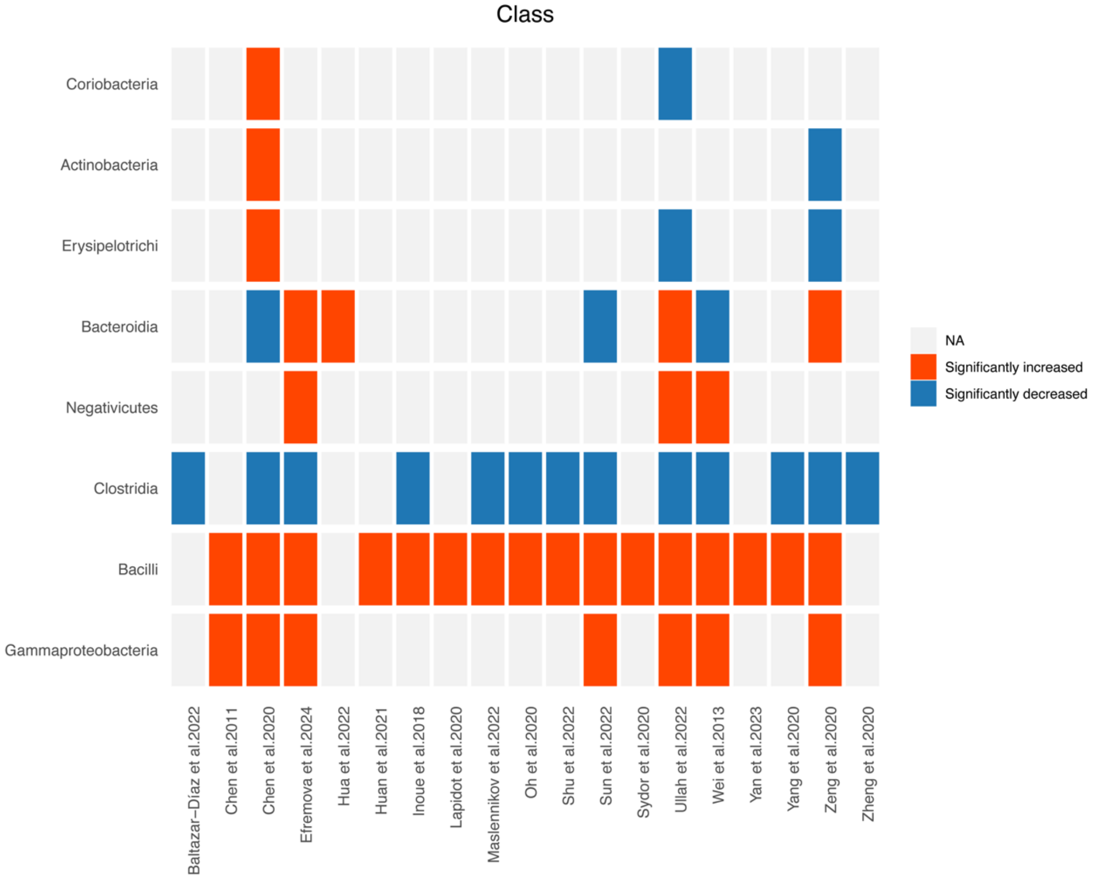

Supplementary Figure S16. Changes of relative abundance of 36 families included in analysis.

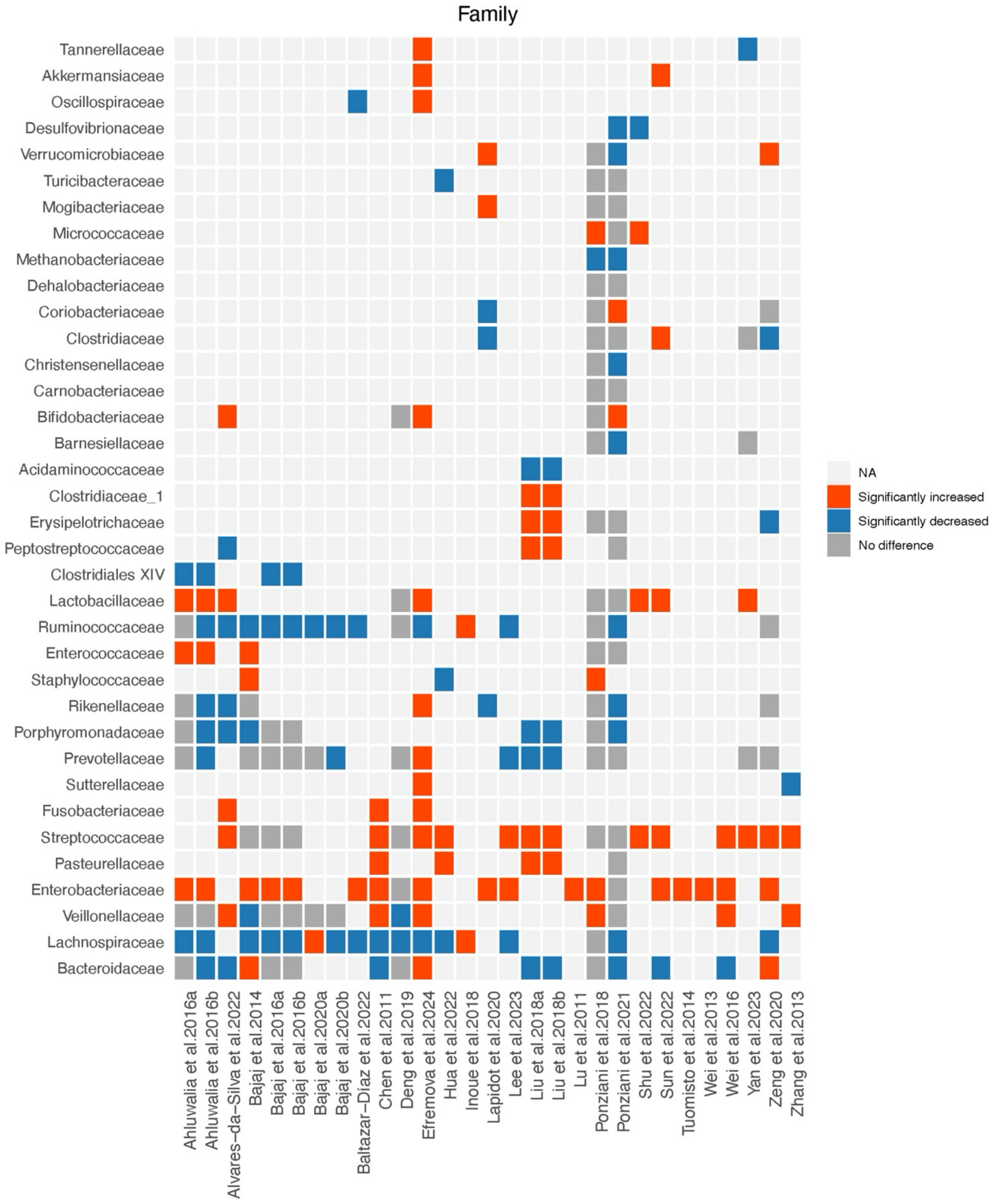

Supplementary Figure S17. Changes of relative abundance of 78 genera included in analysis.

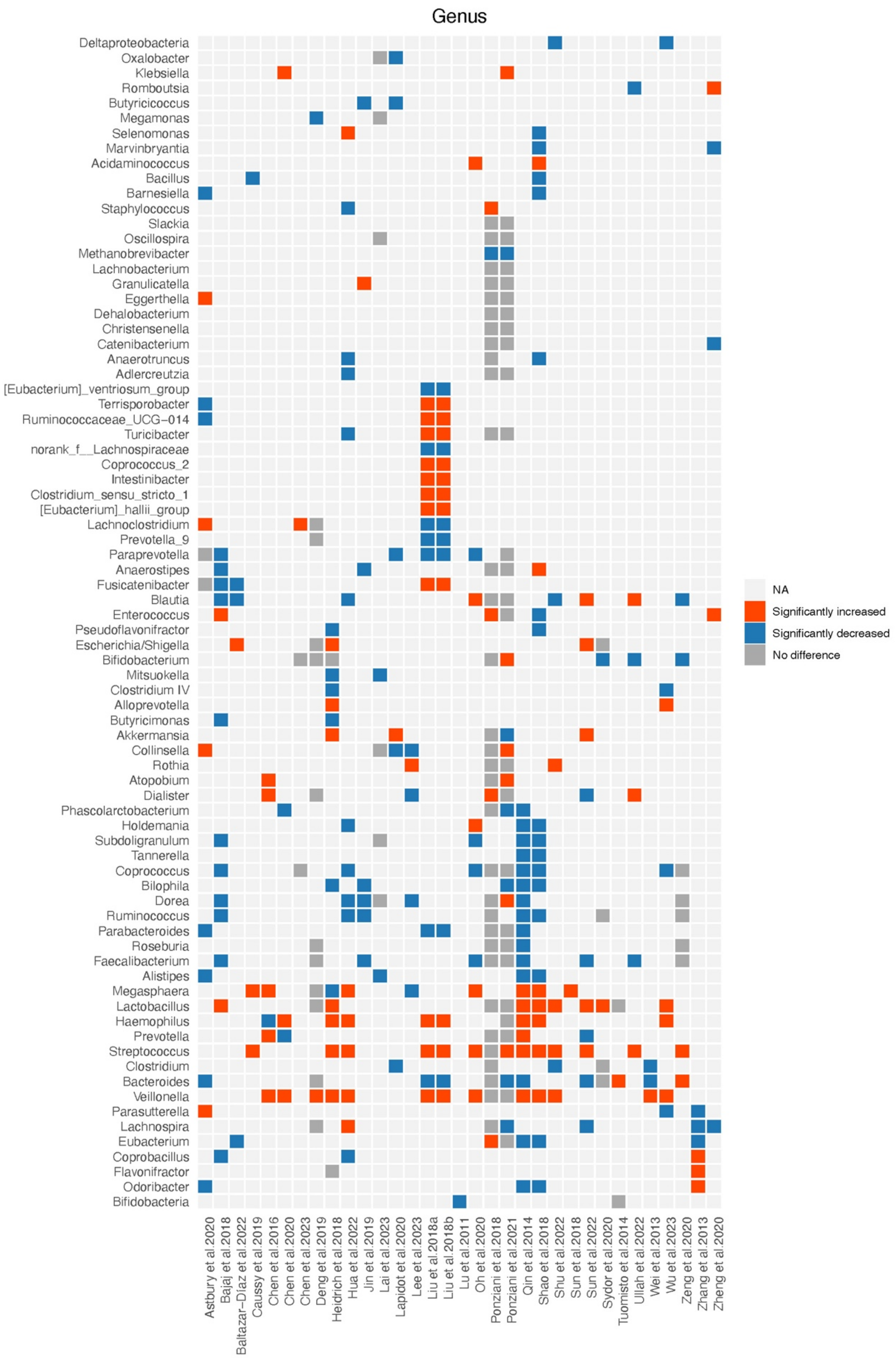

Supplementary Figure S18. A meta-analysis of the cirrhosis dysbiosis ratio.

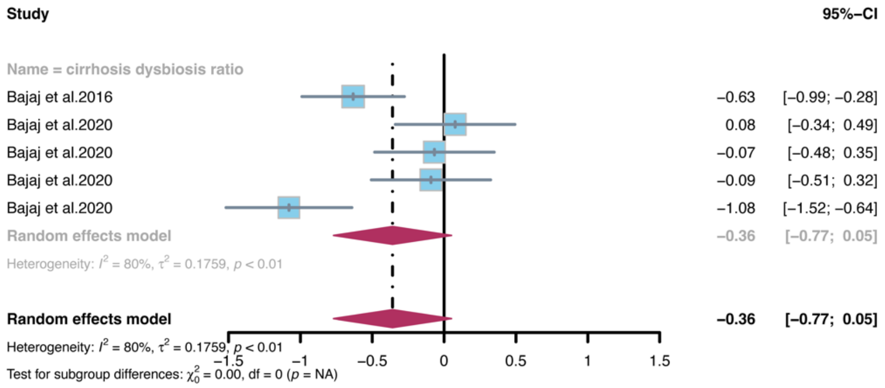

Supplementary Figure S19. A meta-analysis of the reported microbial taxa at phylum level.

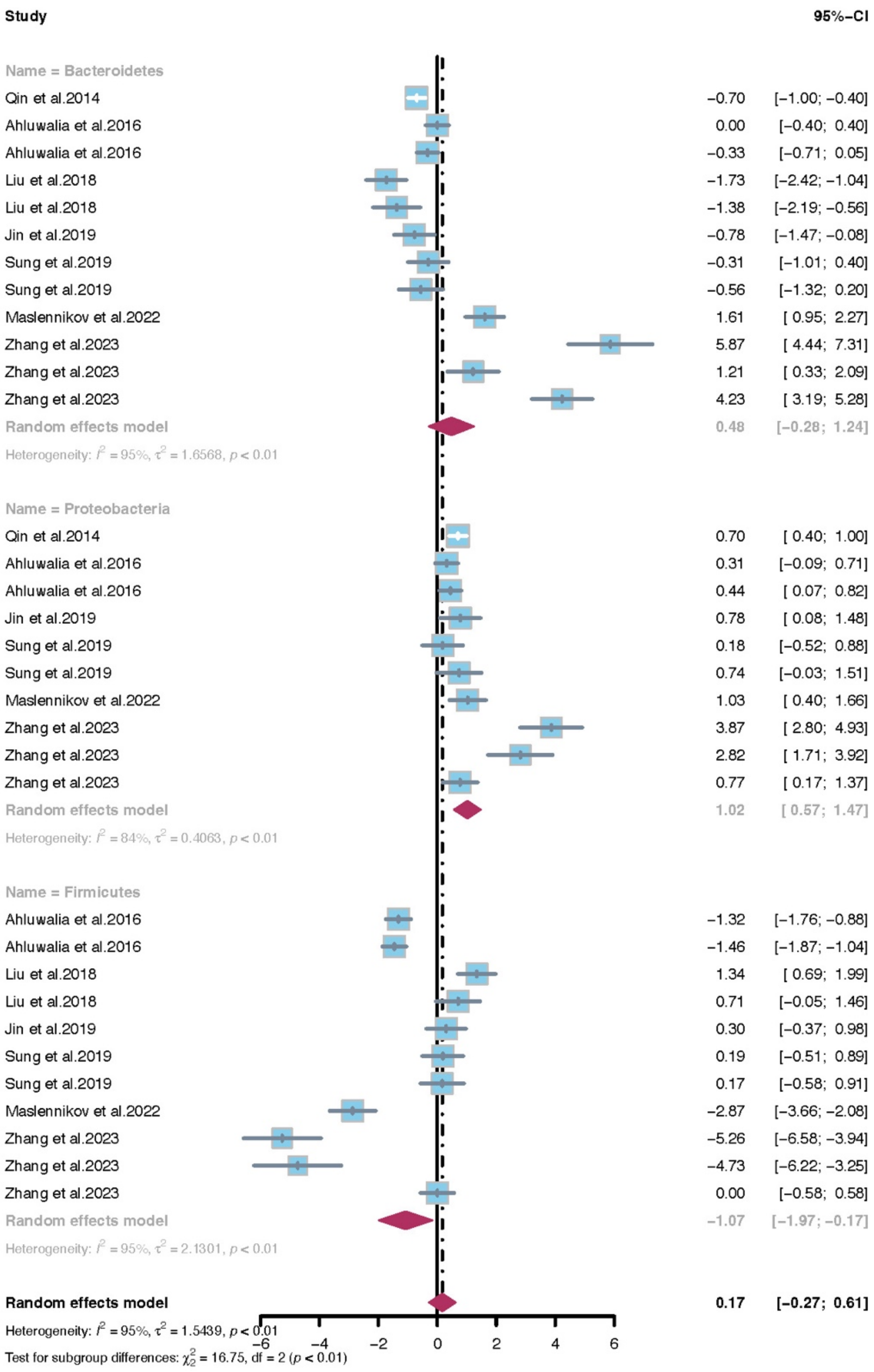

Supplementary Figure S20. A meta-analysis of the reported microbial taxa at class level.

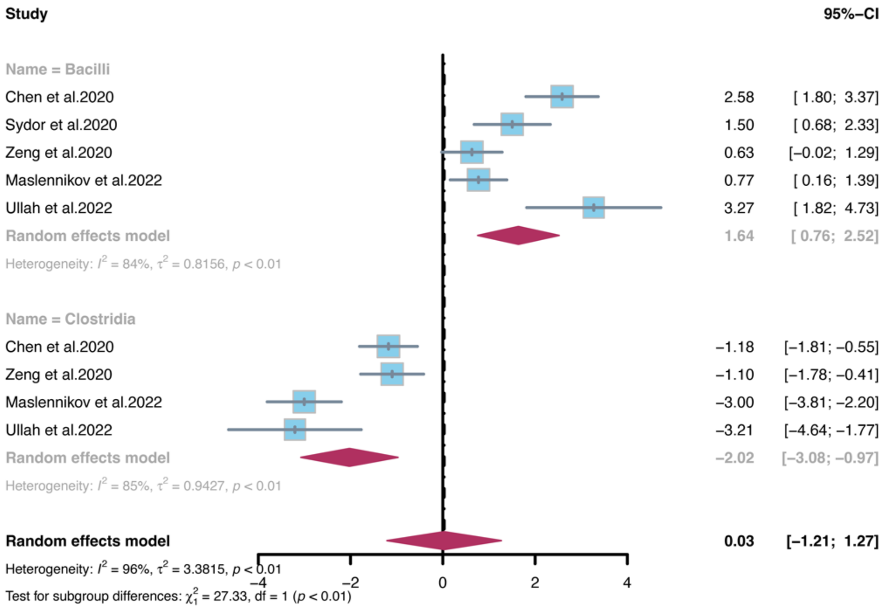

Supplementary Figure S21. A meta-analysis of the reported microbial taxa at family level.

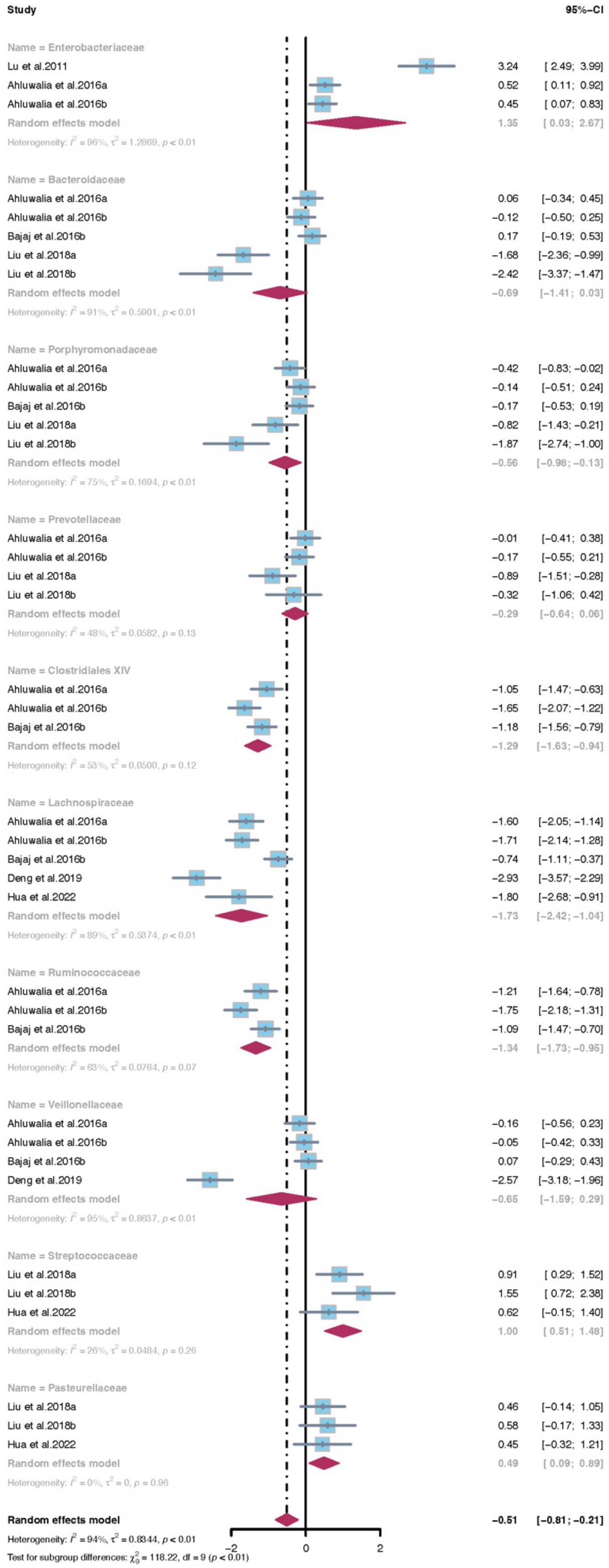

Supplementary Figure S22. A meta-analysis of the reported microbial taxa at genus level.

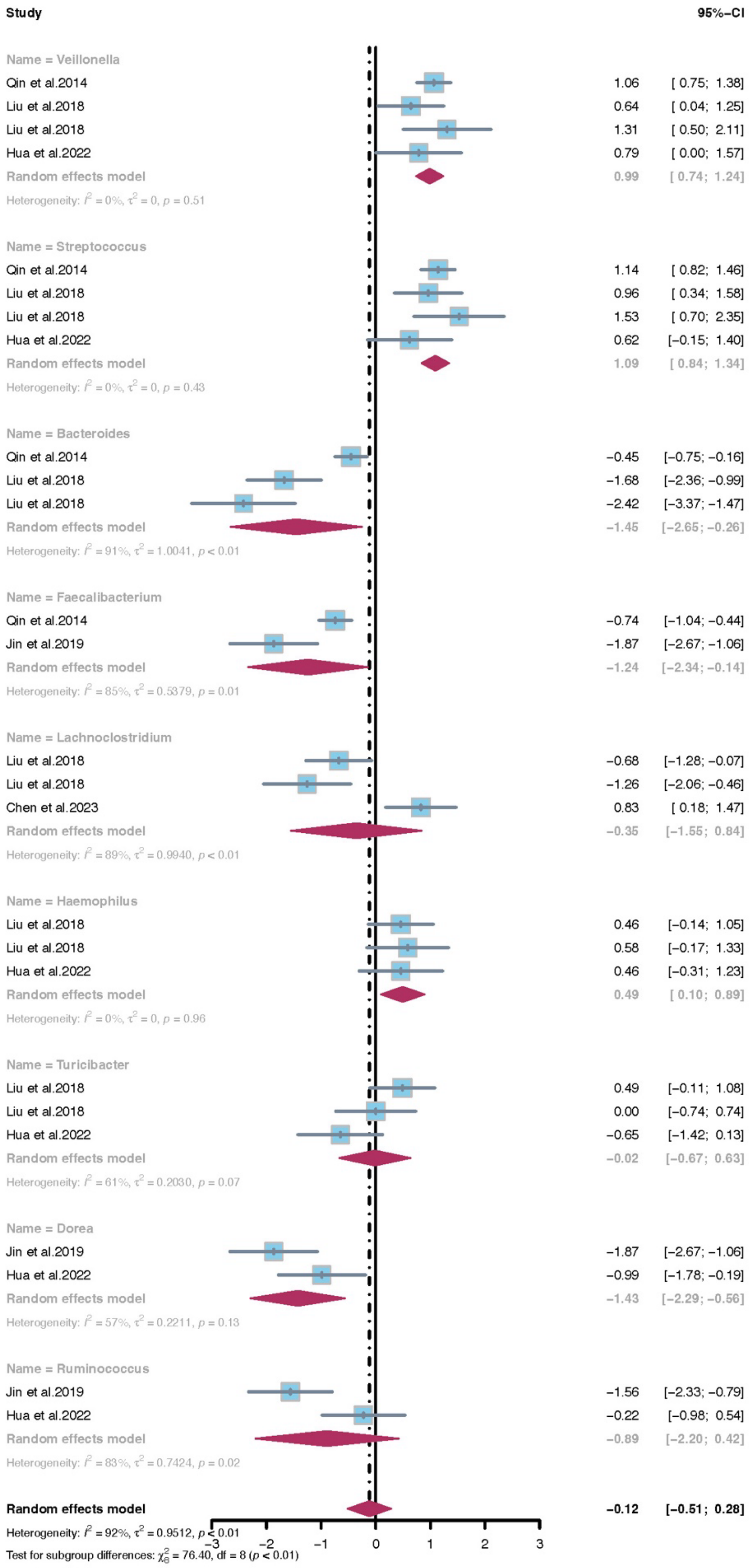

References

1. Chen Y, Yang F, Lu H, Wang B, Chen Y, Lei D, Wang Y, Zhu B, Li L: **Characterization of fecal microbial communities in patients with liver cirrhosis**. *Hepatology* 2011, **54**(2):562-572.
2. Lu H, Wu Z, Xu W, Yang J, Chen Y, Li L: **Intestinal Microbiota Was Assessed in Cirrhotic Patients with Hepatitis B Virus Infection**. *Microbial Ecology* 2011, **61**(3):693-703.
3. Zhang Z, Zhai H, Geng J, Yu R, Ren H, Fan H, Shi P: **Large-scale survey of gut microbiota associated with MHE Via 16S rRNA-based pyrosequencing**. *Am J Gastroenterol* 2013, **108**(10):1601-1611.
4. Wei X, Yan X, Zou D, Yang Z, Wang X, Liu W, Wang S, Li X, Han J, Huang L *et al*: **Abnormal fecal microbiota community and functions in patients with hepatitis B liver cirrhosis as revealed by a metagenomic approach**. *BMC Gastroenterology* 2013, **13**(1).
5. Bajaj JS, Heuman DM, Hylemon PB, Sanyal AJ, White MB, Monteith P, Noble NA, Unser AB, Daita K, Fisher AR *et al*: **Altered profile of human gut microbiome is associated with cirrhosis and its complications**. *J Hepatol* 2014, **60**(5):940-947.
6. Qin N, Yang F, Li A, Prifti E, Chen Y, Shao L, Guo J, Le Chatelier E, Yao J, Wu L *et al*: **Alterations of the human gut microbiome in liver cirrhosis**. *Nature* 2014, **513**(7516):59-64.
7. Tuomisto S, Pessi T, Collin P, Vuento R, Aittoniemi J, Karhunen PJ: **Changes in gut bacterial populations and their translocation into liver and ascites in alcoholic liver cirrhotics**. *BMC Gastroenterology* 2014, **14**(1).
8. Ahluwalia V, Betrapally NS, Hylemon PB, White MB, Gillevet PM, Unser AB, Fagan A, Daita K, Heuman DM, Zhou H *et al*: **Impaired Gut-Liver-Brain Axis in Patients with Cirrhosis**. *Sci Rep* 2016, **6**:26800.
9. Bajaj JS, Sterling R, Hylemon P, White MB, Unser A, Heuman D, Fuchs M, Nixon D, Sikaroodi M, Gillevet PM: **HCV eradication does not impact gut dysbiosis or systemic inflammation in cirrhotic patients**. *Gastroenterology* 2016, **150**(4):S219.
10. Chen Y, Ji F, Guo J, Shi D, Fang D, Li L: **Dysbiosis of small intestinal microbiota in liver cirrhosis and its association with etiology**. *Sci Rep* 2016, **6**:34055.
11. Wei X, Jiang S, Zhao X, Li H, Lin W, Li B, Lu J, Sun Y, Yuan J: **Community-Metabolome Correlations of Gut Microbiota from Child-Turcotte-Pugh of A and B Patients**. *Front Microbiol* 2016, **7**:1856.
12. Lee PC, Lee KC, Yang TC, Lu HS, Cheng TY, Chen YJ, Chiou JJ, Huang CW, Yang UC, Chia-Hui Tan E *et al*: **Sarcopenia-related gut microbial changes are associated with the risk of complications in people with cirrhosis**. *JHEP Rep* 2023, **5**(1):100619.
13. Heidrich B, Vital M, Plumeier I, Döscher N, Kahl S, Kirschner J, Ziegert S, Solbach P, Lenzen H, Potthoff A *et al*: **Intestinal microbiota in patients with chronic hepatitis C with and without cirrhosis compared with healthy controls**. *Liver Int* 2018, **38**(1):50-58.
14. Bajaj JS, Idilman R, Mabudian L, Hood M, Fagan A, Turan D, White MB, Karakaya F, Wang J, Atalay R *et al*: **Diet affects gut microbiota and modulates hospitalization risk differentially in an international cirrhosis cohort**. *Hepatology* 2018, **68**(1):234-247.
15. Bajaj JS, Liu EJ, Kheradman R, Fagan A, Heuman DM, White M, Gavis EA, Hylemon P, Sikaroodi M, Gillevet PM: **Fungal dysbiosis in cirrhosis**. *Gut* 2018, **67**(6):1146-1154.

16. Bajaj JS, Thacker LR, Fagan A, White MB, Gavis EA, Hylemon PB, Brown R, Acharya C, Heuman DM, Fuchs M *et al*: **Gut microbial RNA and DNA analysis predicts hospitalizations in cirrhosis**. *JCI Insight* 2018, **3**(5).

17. Inoue T, Nakayama J, Moriya K, Kawaratani H, Momoda R, Ito K, Iio E, Nojiri S, Fujiwara K, Yoneda M *et al*: **Gut Dysbiosis Associated With Hepatitis C Virus Infection**. *Clin Infect Dis* 2018, **67**(6):869-877.

18. Liu Y, Jin Y, Li J, Zhao L, Li Z, Xu J, Zhao F, Feng J, Chen H, Fang C *et al*: **Small Bowel Transit and Altered Gut Microbiota in Patients With Liver Cirrhosis**. *Front Physiol* 2018, **9**:470.

19. Ponziani FR, Putignani L, Paroni Sterbini F, Petito V, Picca A, Del Chierico F, Reddel S, Calvani R, Marzetti E, Sanguinetti M *et al*: **Influence of hepatitis C virus eradication with direct-acting antivirals on the gut microbiota in patients with cirrhosis**. *Aliment Pharmacol Ther* 2018, **48**(11-12):1301-1311.

20. Shao L, Ling Z, Chen D, Liu Y, Yang F, Li L: **Disorganized Gut Microbiome Contributed to Liver Cirrhosis Progression: A Meta-Omics-Based Study**. *Front Microbiol* 2018, **9**:3166.

21. Sun YL, Li WQ, Ding PX, Wang ZW, Wei CH, Ma XX, Zhang RF, Wu Y, Zhou L, Liang RP *et al*: **Specific alterations in gut microbiota are associated with prognosis of Budd-Chiari syndrome**. *Oncotarget* 2018, **9**(3):3303-3320.

22. Caussy C, Tripathi A, Humphrey G, Bassirian S, Singh S, Faulkner C, Bettencourt R, Rizo E, Richards L, Xu ZZ *et al*: **A gut microbiome signature for cirrhosis due to nonalcoholic fatty liver disease**. *Nat Commun* 2019, **10**(1):1406.

23. Deng YD, Peng XB, Zhao RR, Ma CQ, Li JN, Yao LQ: **The intestinal microbial community dissimilarity in hepatitis B virus-related liver cirrhosis patients with and without at alcohol consumption**. *Gut Pathog* 2019, **11**:58.

24. Jin M, Kalainy S, Baskota N, Chiang D, Deehan EC, McDougall C, Tandon P, Martínez I, Cervera C, Walter J *et al*: **Faecal microbiota from patients with cirrhosis has a low capacity to ferment non-digestible carbohydrates into short-chain fatty acids**. *Liver Int* 2019, **39**(8):1437-1447.

25. Zheng R, Wang G, Pang Z, Ran N, Gu Y, Guan X, Yuan Y, Zuo X, Pan H, Zheng J *et al*: **Liver cirrhosis contributes to the disorder of gut microbiota in patients with hepatocellular carcinoma**. *Cancer Med* 2020, **9**(12):4232-4250.

26. Sung CM, Lin YF, Chen KF, Ke HM, Huang HY, Gong YN, Tsai WS, You JF, Lu MJ, Cheng HT *et al*: **Predicting Clinical Outcomes of Cirrhosis Patients With Hepatic Encephalopathy From the Fecal Microbiome**. *Cell Mol Gastroenterol Hepatol* 2019, **8**(2):301-318 e302.

27. Astbury S, Atallah E, Vijay A, Aithal GP, Grove JI, Valdes AM: **Lower gut microbiome diversity and higher abundance of proinflammatory genus Collinsella are associated with biopsy-proven nonalcoholic steatohepatitis**. *Gut Microbes* 2020, **11**(3):569-580.

28. Bajaj JS, Torre A, Rojas ML, Fagan A, Nandez IE, Gavis EA, De Leon Osorio O, White MB, Fuchs M, Sikaroodi M *et al*: **Cognition and hospitalizations are linked with salivary and faecal microbiota in cirrhosis cohorts from the USA and Mexico**. *Liver Int* 2020, **40**(6):1395-1407.

29. Cox IJ, Idilman R, Fagan A, Turan D, Ajayi L, Le Guennec AD, Taylor-Robinson SD, Karakaya F, Gavis E, Andrew Atkinson R *et al*: **Metabolomics and microbial composition**

increase insight into the impact of dietary differences in cirrhosis. *Liver Int* 2020, **40**(2):416-427.

30. Lapidot Y, Amir A, Nosenko R, Uzan-Yulzari A, Veitsman E, Cohen-Ezra O, Davidov Y, Weiss P, Bradichevski T, Segev S *et al*: **Alterations in the Gut Microbiome in the Progression of Cirrhosis to Hepatocellular Carcinoma**. *mSystems* 2020, **5**(3).
31. Oh TG, Kim SM, Caussy C, Fu T, Guo J, Bassirian S, Singh S, Madamba EV, Bettencourt R, Richards L *et al*: **A Universal Gut-Microbiome-Derived Signature Predicts Cirrhosis**. *Cell Metab* 2020, **32**(5):901.
32. Sydor S, Best J, Messerschmidt I, Manka P, Vilchez-Vargas R, Brodesser S, Lucas C, Wegehaupt A, Wenning C, Aßmuth S *et al*: **Altered Microbiota Diversity and Bile Acid Signaling in Cirrhotic and Noncirrhotic NASH-HCC**. *Clin Transl Gastroenterol* 2020, **11**(3):e00131.
33. Yang XA, Lv F, Wang R, Chang Y, Zhao Y, Cui X, Li H, Yang S, Li S, Zhao X *et al*: **Potential role of intestinal microflora in disease progression among patients with different stages of Hepatitis B**. *Gut Pathog* 2020, **12**:50.
34. Zeng Y, Chen S, Fu Y, Wu W, Chen T, Chen J, Yang B, Ou Q: **Gut microbiota dysbiosis in patients with hepatitis B virus-induced chronic liver disease covering chronic hepatitis, liver cirrhosis and hepatocellular carcinoma**. *J Viral Hepat* 2020, **27**(2):143-155.
35. Huan H, Ren T, Xu L, Hu H, Liu C: **Compositional distinction of gut microbiota between Han Chinese and Tibetan populations with liver cirrhosis**. *PeerJ* 2021, **9**:e12142.
36. Ponziani FR, Picca A, Marzetti E, Calvani R, Conta G, Del Chierico F, Capuani G, Faccia M, Fianchi F, Funaro B *et al*: **Characterization of the gut-liver-muscle axis in cirrhotic patients with sarcopenia**. *Liver Int* 2021, **41**(6):1320-1334.
37. Ren X, Hao S, Yang C, Yuan L, Zhou X, Zhao H, Yao J: **Alterations of intestinal microbiota in liver cirrhosis with muscle wasting**. *Nutrition* 2021, **83**:111081.
38. Alvares-da-Silva MR, Oliveira CP, Fagan A, Longo L, Thoen RU, Yoshimura Zitelli PM, Tanaka Ferreira RM, McGeorge S, Shamsaddini A, Farias AQ *et al*: **Interaction of Microbiome, Diet, and Hospitalizations Between Brazilian and American Patients With Cirrhosis**. *Clin Gastroenterol Hepatol* 2022, **20**(4):930-940.
39. Baltazar-Díaz TA, González-Hernández LA, Aldana-Ledesma JM, Peña-Rodríguez M, Vega-Magaña AN, Zepeda-Morales ASM, López-Roa RI, Del Toro-Arreola S, Martínez-López E, Salazar-Montes AM *et al*: **Escherichia/Shigella, SCFAs, and Metabolic Pathways-The Triad That Orchestrates Intestinal Dysbiosis in Patients with Decompensated Alcoholic Cirrhosis from Western Mexico**. *Microorganisms* 2022, **10**(6).
40. Hua X, Feng H: **Changes in intestinal microbiota of HBV-associated liver cirrhosis with/without hepatic encephalopathy**. *Medicine (Baltimore)* 2022, **101**(33):e29935.
41. Maslennikov R, Ivashkin V, Alieva A, Poluektova E, Kudryavtseva A, Krasnov G, Zharkova M, Zharikov Y: **Gut dysbiosis and body composition in cirrhosis**. *World J Hepatol* 2022, **14**(6):1210-1225.
42. Shu W, Shanjian C, Jinpiao L, Qishui O: **Gut microbiota dysbiosis in patients with hepatitis B virus-related cirrhosis**. *Ann Hepatol* 2022, **27**(2):100676.

43. Sun X, Chi X, Zhao Y, Liu S, Xing H: **Characteristics and Clinical Significance of Intestinal Microbiota in Patients with Chronic Hepatitis B Cirrhosis and Type 2 Diabetes Mellitus**. *J Diabetes Res* 2022, **2022**:1826181.
44. Ullah N, Kakakhel MA, Khan I, Gul Hilal M, Lajia Z, Bai Y, Sajjad W, Yuxi L, Ullah H, Almohaimeed HM *et al*: **Structural and compositional segregation of the gut microbiota in HCV and liver cirrhotic patients: A clinical pilot study**. *Microb Pathog* 2022, **171**:105739.
45. Zhou Z, Lv H, Lv J, Shi Y, Huang H, Chen L, Shi D: **Alterations of gut microbiota in cirrhotic patients with spontaneous bacterial peritonitis: A distinctive diagnostic feature**. *Front Cell Infect Microbiol* 2022, **12**:999418.
46. Chen Y, Chen S, Xu C, Yu L, Chu S, Bao J, Wang J, Wang J: **Identification of Diagnostic Biomarkers for Compensatory Liver Cirrhosis Based on Gut Microbiota and Urine Metabolomics Analyses**. *Mol Biotechnol* 2023.
47. Lai MW, Chu YD, Hsu CW, Chen YC, Liang KH, Yeh CT: **Multi-Omics Analyses Identify Signatures in Patients with Liver Cirrhosis and Hepatocellular Carcinoma**. *Cancers* 2023, **15**(1).
48. Wang Q, Chen C, Zuo S, Cao K, Li H: **Integrative analysis of the gut microbiota and faecal and serum short-chain fatty acids and tryptophan metabolites in patients with cirrhosis and hepatic encephalopathy**. *J Transl Med* 2023, **21**(1):395.
49. Wu Z, Zhou H, Liu D, Deng F: **Alterations in the gut microbiota and the efficacy of adjuvant probiotic therapy in liver cirrhosis**. *Front Cell Infect Microbiol* 2023, **13**:1218552.
50. Yan F, Zhang Q, Shi K, Zhang Y, Zhu B, Bi Y, Wang X: **Gut microbiota dysbiosis with hepatitis B virus liver disease and association with immune response**. *Front Cell Infect Microbiol* 2023, **13**:1152987.
51. Zhang H, Wu J, Liu Y, Zeng Y, Jiang Z, Yan H, Lin J, Zhou W, Ou Q, Ao L: **Identification reproducible microbiota biomarkers for the diagnosis of cirrhosis and hepatocellular carcinoma**. *AMB Express* 2023, **13**(1):35.
52. Efremova I, Maslennikov R, Poluektova E, Medvedev O, Kudryavtseva A, Krasnov G, Fedorova M, Romanikhin F, Bakhitov V, Aliev S *et al*: **Gut Microbiota and Biomarkers of Endothelial Dysfunction in Cirrhosis**. *Int J Mol Sci* 2024, **25**(4).
53. Chen Z, Xie Y, Zhou F, Zhang B, Wu J, Yang L, Xu S, Stedtfeld R, Chen Q, Liu J *et al*: **Featured Gut Microbiomes Associated With the Progression of Chronic Hepatitis B Disease**. *Front Microbiol* 2020, **11**:383.
54. Li L, Ding HG: **The analysis of the DNA fingerpring of intestinal flora in liver cirrhotic patients**. *Hepatology International* 2010, **4**(1):257.
55. Bajaj JS, Ridlon JM, Hylemon PB, Thacker LR, Heuman DM, Smith S, Sikaroodi M, Gillevet PM: **Linkage of gut microbiome with cognition in hepatic encephalopathy**. *Am J Physiol Gastrointest Liver Physiol* 2012, **302**(1):G168-175.
56. Bajaj JS, Betrapally NS, Hylemon PB, Heuman DM, Daita K, White MB, Unser A, Thacker LR, Sanyal AJ, Kang DJ *et al*: **Salivary microbiota reflects changes in gut microbiota in cirrhosis with hepatic encephalopathy**. *Hepatology* 2015, **62**(4):1260-1271.

57. Bajaj JS, Betrapally NS, Hylemon PB, Thacker LR, Daita K, Kang DJ, White MB, Unser AB, Fagan A, Gavis EA *et al*: **Gut Microbiota Alterations can predict Hospitalizations in Cirrhosis Independent of Diabetes Mellitus**. *Sci Rep* 2015, **5**:18559.
58. Flass T, Tong S, Frank DN, Wagner BD, Robertson CE, Kotter CV, Sokol RJ, Zemanick E, Accurso F, Hoffenberg EJ *et al*: **Intestinal lesions are associated with altered intestinal microbiome and are more frequent in children and young adults with cystic fibrosis and cirrhosis**. *PLoS One* 2015, **10**(2):e0116967.
59. Ponziani FR, Gasbarrini A, Pompili M: **Letter to the Editor: Restructuring the Gut Microbiota of Patients with Cirrhosis After HCV Eradication: A Matter of Time?** *Hepatology* 2021, **74**(3):1718.
60. Cao X, Zolnikova O, Maslennikov R, Reshetova M, Poluektova E, Bogacheva A, Zharkova M, Ivashkin V: **Differences in Fecal Short-Chain Fatty Acids between Alcoholic Fatty Liver-Induced Cirrhosis and Non-alcoholic (Metabolic-Associated) Fatty Liver-Induced Cirrhosis**. *Metabolites* 2023, **13**(7).
61. Höppner J, Krohn S, van den Munckhof EHA, Kallies R, Herber A, Zeller K, Tünnemann J, Matz-Soja M, Chatzinotas A, Böhm S *et al*: **Changes of the bacterial composition in duodenal fluid from patients with liver cirrhosis and molecular bacterascites**. *Sci Rep* 2023, **13**(1):23001.
62. Bajaj JS, Hylemon PB, Ridlon JM, Heuman DM, Daita K, White MB, Monteith P, Noble NA, Sikaroodi M, Gillevet PM: **Colonic mucosal microbiome differs from stool microbiome in cirrhosis and hepatic encephalopathy and is linked to cognition and inflammation**. *Am J Physiol Gastrointest Liver Physiol* 2012, **303**(6):G675-685.
63. Liu J, Wu D, Ahmed A, Li X, Ma Y, Tang L, Mo D, Ma Y, Xin Y: **Comparison of the gut microbe profiles and numbers between patients with liver cirrhosis and healthy individuals**. *Curr Microbiol* 2012, **65**(1):7-13.
64. Mou H, Yang F, Zhou J, Bao C: **Correlation of liver function with intestinal flora, vitamin deficiency and IL-17A in patients with liver cirrhosis**. *Exp Ther Med* 2018, **16**(5):4082-4088.
65. Cox IJ, Peña Rodríguez M, Fagan A, Rojas-Lara MV, Le Guennec A, Rodriguez-Alvarez F, McGeorge S, Escalona-Nandez I, Torre A, Bajaj JS: **Stool microbiota show greater linkages with plasma metabolites compared to salivary microbiota in a multinational cirrhosis cohort**. *Liver International* 2022, **42**(10):2274-2282.
66. Chen Y, Chen Z, Guo R, Chen N, Lu H, Huang S, Wang J, Li L: **Correlation between gastrointestinal fungi and varying degrees of chronic hepatitis B virus infection**. *Diagnostic Microbiology and Infectious Disease* 2011, **70**(4):492-498.
67. Aliwa B, Horvath A, Traub J, Feldbacher N, Habisch H, Fauler G, Madl T, Stadlbauer V: **Altered gut microbiome, bile acid composition and metabolome in sarcopenia in liver cirrhosis**. *J Cachexia Sarcopenia Muscle* 2023, **14**(6):2676-2691.
68. Ciocan D, Voican CS, Wrzosek L, Hugot C, Rainteau D, Humbert L, Cassard AM, Perlemuter G: **Bile acid homeostasis and intestinal dysbiosis in alcoholic hepatitis**. *Aliment Pharmacol Ther* 2018, **48**(9):961-974.
69. Usami M, Miyoshi M, Kanbara Y, Aoyama M, Sakaki H, Shuno K, Hirata K, Takahashi M, Ueno K, Hamada Y *et al*: **Analysis of fecal microbiota, organic acids and plasma lipids in hepatic cancer patients with or without liver cirrhosis**. *Clin Nutr* 2013, **32**(3):444-451.

70. Grat M, Wronka KM, Krasnodebski M, Masior L, Lewandowski Z, Kosinska I, Grat K, Stypulkowski J, Rejowski S, Wasilewicz M *et al*: **Profile of Gut Microbiota Associated With the Presence of Hepatocellular Cancer in Patients With Liver Cirrhosis**. *Transplant Proc* 2016, **48**(5):1687-1691.
71. Jacobs JP, Dong TS, Agopian V, Lagishetty V, Sundaram V, Noureddin M, Ayoub WS, Durazo F, Benhammou J, Enayati P *et al*: **Microbiome and bile acid profiles in duodenal aspirates from patients with liver cirrhosis: The Microbiome, Microbial Markers and Liver Disease Study**. *Hepatology Research* 2018, **48**(13):1108-1117.
72. Bajaj JS, Sikaroodi M, Fagan A, Heuman D, Gilles H, Gavis EA, Fuchs M, Gonzalez-Maeso J, Nizam S, Gillevet PM *et al*: **Posttraumatic stress disorder is associated with altered gut microbiota that modulates cognitive performance in veterans with cirrhosis**. *Am J Physiol Gastrointest Liver Physiol* 2019, **317**(5):G661-g669.
73. Bajaj JS, Vargas HE, Reddy KR, Lai JC, O'Leary JG, Tandon P, Wong F, Mitrani R, White MB, Kelly M *et al*: **Association Between Intestinal Microbiota Collected at Hospital Admission and Outcomes of Patients With Cirrhosis**. *Clin Gastroenterol Hepatol* 2019, **17**(4):756-765 e753.
74. Bajaj JS, Reddy KR, Tandon P, Garcia-Tsao G, Kamath PS, O'Leary JG, Wong F, Lai J, Vargas H, Thuluvath PJ *et al*: **Association of serum metabolites and gut microbiota at hospital admission with nosocomial infection development in patients with cirrhosis**. *Liver Transpl* 2022, **28**(12):1831-1840.
75. Bajaj JS, Rodriguez MP, Fagan A, McGeorge S, Sterling RK, Lee H, Luketic V, Fuchs M, Davis BC, Sikaroodi M et al: Impact of **bacterial infections and spontaneous bacterial peritonitis prophylaxis on phage-bacterial dynamics in cirrhosis**. *Hepatology* 2022, **76**(6):1723-1734.
76. Bajaj JS, Shamsaddini A, Sikaroodi M, Davis B, Puri P, Fuchs M, Fagan A, McGeorge S, Gillevet P: **Active alcohol misuse is linked with lower short-chain fatty acid producing microbiota in a matched study of 450 patients with cirrhosis**. *Journal of Hepatology* 2022, **77**:S130-S131.
77. Haraguchi M, Miuma S, Masumoto H, Ichikawa T, Kanda Y, Sasaki R, Fukushima M, Miyaaki H, Taura N, Nakao K: **Bacteroides in colonic mucosa-associated microbiota affects the development of minimal hepatic encephalopathy in patients with cirrhosis**. *Hepatol Int* 2019, **13**(4):482-489.
78. Lang S, Farowski F, Martin A, Wisplinghoff H, Vehreschild M, Krawczyk M, Nowag A, Kretzschmar A, Scholz C, Kasper P *et al*: **Prediction of advanced fibrosis in non-alcoholic fatty liver disease using gut microbiota-based approaches compared with simple non-invasive tools**. *Sci Rep* 2020, **10**(1):9385.
79. Bajaj JS, Shamsaddini A, Fagan A, McGeorge S, Gavis E, Sikaroodi M, Brenner LA, Wade JB, Gillevet PM: **Distinct gut microbial compositional and functional changes associated with impaired inhibitory control in patients with cirrhosis**. *Gut Microbes* 2021, **13**(1):1953247.
80. Shen TD, Daniel SG, Patel S, Kaplan E, Phung L, Lemelle-Thomas K, Chau L, Herman L, Trisolini C, Stonelake A *et al*: **The Mucosally-Adherent Rectal Microbiota Contains Features Unique to Alcohol-Related Cirrhosis**. *Gut Microbes* 2021, **13**(1):1987781.

81. Lin MJ, Su TH, Chen CC, Wu WK, Hsu SJ, Tseng TC, Liao SH, Hong CM, Yang HC, Liu CJ *et al*: **Diversity and composition of gut microbiota in healthy individuals and patients at different stages of hepatitis B virus-related liver disease**. *Gut Pathog* 2023, **15**(1):24.
82. Naseri M, Houri H, Yadegar A, Asadzadeh Aghdaei H, Zahiri J: **Investigation of etiology-specific alterations in the gut microbiota in liver cirrhosis**. *Expert Review of Gastroenterology & Hepatology* 2021, **15**(12):1435-1441.
83. Luo M, Hu FR, Xin RJ, Yao L, Hu SJ, Bai FH: **Altered gut microbiota is associated with sleep disturbances in patients with minimal hepatic encephalopathy caused by hepatitis B-related liver cirrhosis**. *Expert Rev Gastroenterol Hepatol* 2022, **16**(8):797-807.
84. Maslennikov R, Ivashkin V, Efremova I, Poluektova E, Kudryavtseva A, Krasnov G: **Gut dysbiosis and small intestinal bacterial overgrowth as independent forms of gut microbiota disorders in cirrhosis**. *World Journal of Gastroenterology* 2022, **28**(10):1067-1077.
85. Naseri M, Palizban F, Yadegar A, Khodarahmi M, Asadzadeh Aghdaei H, Houri H, Zahiri J: **Investigation and characterization of human gut phageome in advanced liver cirrhosis of defined etiologies**. *Gut Pathog* 2022, **14**(1):9.
86. Bajaj JS, Peña-Rodriguez M, La Reau A, Phillips W, Fuchs M, Davis BC, Sterling RK, Sikaroodi M, Fagan A, Shamsaddini A *et al*: **Longitudinal transkingdom gut microbial approach towards decompensation in outpatients with cirrhosis**. *Gut* 2023, **72**(4):759-771.
87. Effenberger M, Waschina S, Bronowski C, Sturm G, Tassiello O, Sommer F, Zollner A, Watschinger C, Grabherr F, Gstir R *et al*: **A gut bacterial signature in blood and liver tissue characterizes cirrhosis and hepatocellular carcinoma**. *Hepatol Commun* 2023, **7**(7).
88. Huang XY, Zhang YH, Yi SY, Lei L, Ma T, Huang R, Yang L, Li ZM, Zhang D: **Potential contribution of the gut microbiota to the development of portal vein thrombosis in liver cirrhosis**. *Front Microbiol* 2023, **14**:1217338.
89. Jinato T, Sikaroodi M, Fagan A, Sterling RK, Lee H, Puri P, Davis BC, Fuchs M, Gavis E, Gillevet PM *et al*: **Alterations in gut virome are associated with cognitive function and minimal hepatic encephalopathy cross-sectionally and longitudinally in cirrhosis**. *Gut Microbes* 2023, **15**(2):2288168.
90. Chang KC, Chang MH, Wu JF, Hsu HY, Chen HL, Ni YH: **Alterations of gut microbiota in biliary atresia patients with liver cirrhosis**. *Journal of Pediatric Gastroenterology and Nutrition* 2019, **68**:731-732.
91. Wellhoner F, Doscher N, Woelfl F, Vital M, Plumeier I, Kahl S, Potthoff A, Manns MP, Pieper DH, Cornberg M *et al*: **Eradication of Chronic HCV Infection: Improvement of Dysbiosis Only in Patients Without Liver Cirrhosis**. *Hepatology* 2021, **74**(1):72-82.
